# Mapping the genetic landscape of immune-mediated disorders: potential implications for classification and therapeutic strategies

**DOI:** 10.3389/fimmu.2025.1543781

**Published:** 2025-05-08

**Authors:** Vera Fominykh, Alexey A. Shadrin, Piotr Jaholkowski, Julian Fuhrer, Nadine Parker, Erik D. Wiström, Oleksandr Frei, Olav B. Smeland, Helga Sanner, Srdjan Djurovic, Ole A. Andreassen

**Affiliations:** ^1^ Centre for Precision Psychiatry, Institute of Clinical Medicine, University of Oslo, Oslo, Norway; ^2^ K.G. Jebsen Centre for Neurodevelopmental Disorders, University of Oslo and Oslo University Hospital, Oslo, Norway; ^3^ Section for Precision Psychiatry, Oslo University Hospital, Oslo, Norway; ^4^ Department of Rheumatology, Oslo University Hospital, Oslo, Norway; ^5^ Institute for Health, Oslo New University College, Oslo, Norway; ^6^ Institute of Clinical Medicine, University of Oslo, Oslo, Norway; ^7^ Department of Medical Genetics, Oslo University Hospital and University of Oslo, Oslo, Norway

**Keywords:** autoimmunity, inflammation, autoimmune diseases, genome-wide association studies, polygenicity, classification

## Abstract

**Objectives:**

Based on clinical, biomarker, and genetic data, McGonagle and McDermott suggested that autoimmune and autoinflammatory disorders can be classified as a disease continuum from purely autoimmune to autoinflammatory with mixed diseases in between. However, the genetic architecture of this spectrum has not been systematically described. Here, we investigate the continuum of polygenic immune-mediated disorders using genome-wide association studies (GWAS) and statistical genetics methods.

**Methods:**

We mapped the genetic landscape of 15 immune-mediated disorders using GWAS summary statistics and methods including genomic structural equation modeling (genomic SEM), linkage disequilibrium score regression, Local Analysis of [co]Variant Association, and Gaussian causal mixture modeling (MiXeR). We performed enrichment analyses of tissues and biological gene sets using MAGMA.

**Results:**

Genomic SEM suggested a continuum structure with four underlying latent factors from autoimmune diseases at one end to autoinflammatory on the opposite end. Across disorders, we observed a balanced mixture of negative and positive local genetic correlations within the major histocompatibility complex, while outside this region, local genetic correlations were predominantly positive. MiXeR analysis showed large genetic overlap in accordance with the continuum landscape. MAGMA analysis implicated genes associated with known monogenic immune diseases for prominent autoimmune and autoinflammatory component.

**Conclusions:**

Our findings support a polygenic continuum across immune-mediated disorders, with four genetic clusters. The “polygenic autoimmune” and “polygenic autoinflammatory” clusters reside on margins of this continuum. These findings provide insights and lead us to hypothesize that the identified clusters could inform future therapeutical strategies, with patients in the same clusters potentially responding similarly to specific therapies.

## 1 Introduction

During the last 20 years, autoimmune and autoinflammatory disorders have garnered attention in the fields of neurology and rheumatology particularly regarding nosology and treatment. Various new diseases have been described, including autoimmune encephalitis (1), myelin oligodendrocyte glycoprotein-associated diseases, and monogenic autoinflammatory diseases (interferonopathies and adenosine deaminase 2 deficiency) (2–4). The global rise in the prevalence of immune-mediated diseases, as highlighted by epidemiological studies (5, 6), has sparked a need for urgent innovation in the development of classification, curation approaches, and treatment strategies.

The release of new therapeutic options for neuromyelitis optica (7), myasthenia gravis (8), and monogenic immune diseases (9) brings to the fore the importance of clinical conception and classification of immune diseases and their underlying genetics. The current understanding of immune-mediated diseases relies on immunological concepts, which define autoinflammation as a dysregulated activation of innate immune cells, driven by an imbalance of pro- and anti-inflammatory cytokines, which leads to damage of host tissues without a break in immune tolerance (10, 11). Conversely, autoimmunity is characterized by the loss of immune tolerance, the recognition of self-antigens, and the activation of T and B cells, followed by the production of specific autoantibodies and the damage of multiple organs owing to a dysregulated adaptive immune response (12–14).

**Figure 1:**
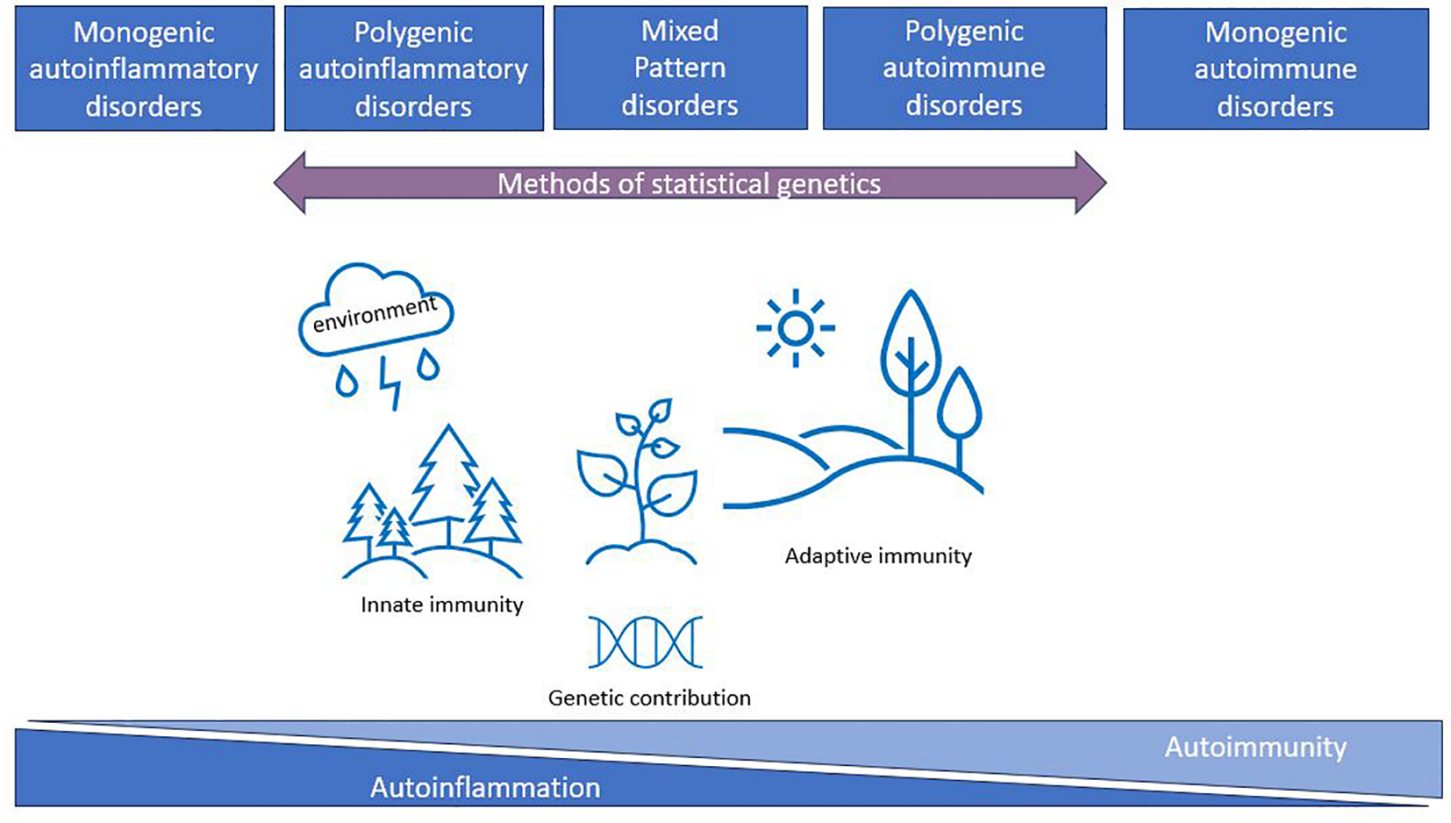
The immunological disease landscape (initial idea taken from (10, 12, 16)). Autoimmune and autoinflammatory diseases were described by McGonagle and McDermott as a continuum with five main classes (monogenic autoinflammatory, polygenic autoinflammatory, mixed pattern, polygenic autoimmune, and monogenic autoimmune disorders) highlighting the importance of genetic factors for the classification. Methods of statistical genetics can work with polygenic disorders and can help to characterize this continuum from genetics perspectives using available GWAS data.

The conceptualization of autoimmune and autoinflammatory diseases as a continuum began with proof-of-concept publications suggesting the classification of immunological diseases based on clinical and laboratory data [(12) updated by (15) and (10)]. This classification described a continuum with five main classes (monogenic autoinflammatory, polygenic autoinflammatory, mixed pattern, polygenic autoimmune, and monogenic autoimmune diseases) highlighting the importance of genetic factors for the classification (**Figure 1**). This classification has become standard practice in rheumatology, where systemic lupus erythematosus (SLE) represents the prototype of systemic autoimmunity with production of multiple autoantibodies. There is still no consensus on the precise classification of some diseases such as rheumatoid arthritis (RA), juvenile idiopathic arthritis (JIA), and ankylosing spondylitis (AS) (17), since they exhibit overlapping features of both autoimmunity and autoinflammation (11). In neurology, some immune-mediated diseases are easier to place on the continuum: for example, myasthenia gravis and anti-LGi1-positive autoimmune encephalitis are classical autoimmune diseases (18, 19). However, for conditions like central nervous system vasculitis and acute encephalomyelitis (20, 21), their placement remains unclear. Since autoimmune and autoinflammatory diseases are widely spread across different medical specialities (neurology, rheumatology, endocrinology, gastroenterology, etc.), current categorization efforts based on clinical features and qualitative markers lead to competing classifications with different hierarchical structures (22, 23). This highlights a need for new methods and data-driven approaches to improve classification.

Immune-mediated diseases have a high degree of comorbidity within families (24, 25), twins (26), and individuals, suggesting shared genetic risk factors across different immune pathologies (27, 28). The idea of genetic-based disease classification has been used previously in cross-disorder genetic analyses of immune diseases (29–32). Nevertheless, most of these studies have used only a limited number of disorders and focus on shared-genetic components as opposed to characterizing genetic-based clusters of disorders. Few studies have systematically evaluated the autoimmune-autoinflammatory continuum itself. A great effort was made to use the AutoCore network for the integration of a set of inborn errors of immunity (33) with predominant autoimmunity or autoinflammation into a comprehensive map of human immune dysregulation (34). The TransImmunome project revisits the nosology of autoimmune and autoinflammatory diseases by combining clinical and biomarkers information (35, 36). State-of-the-art methods of statistical genetics provide an additional opportunity to address this heterogeneity through diseases classification into more homogeneous subgroups based on the underlying genes and pathways that drive disease.

Here, we aim to a) evaluate the hypothesis of autoimmune-autoinflammatory continuum for polygenic disorders based on genome-wide association studies (GWAS) data and b) identify genetically driven clusters of closely related polygenic disorders and their shared genetic factors using publicly available large datasets and advanced methodology. Bridging genetically driven classification with existing clinical knowledge can deepen our understanding of disease-specific molecular mechanisms, guiding further research on classification and potential drug targets.

## 2 Materials and methods

### 2.1 Samples

We curated a collection of well-powered publicly available GWAS summary statistics of 15 polygenic immune-mediated diseases (**Table 1**), which belong to neurological, rheumatological, gastroenterological, and endocrine system domains. The selection procedure is described below. We emphasize that our study is focused exclusively on polygenic disorders within the immune-mediated continuum. Unfortunately, we are unable to include rare monogenic disorders in our analysis, as the statistical genetics methods that we employ are specifically designed to be effective for polygenic disorders.

**Table 1:** Overview of the GWAS used in the study.

Diseases	Abbr.	N cases	N controls	N effective	GWAS source, PMID
Autoimmune thyroiditis	AITD	30,234	725,172	114,296	32581359 (37)
Celiac disease	CeD	6,897	334,824	22,142	20190752 (38), 34278373 (39); for details, see **Supplementary Table S2**.
Crohn’s disease	CD	12,194	34,915	36,151	28067908 (40)
Juvenile idiopathic arthritis	JIA	4,799	294,231	15,670	FinnGen (41), 33106285 (42); for details, see **Supplementary Table S2**
Multiple sclerosis	MS	14,802	26,703	38,093	31604244 (43)
Myasthenia gravis	MG	1,873	36,370	7,125	35074870 (44)
Primary biliary cholangitis	PBC	8,021	16,489	21,584	34033851 (45)
Primary sclerosing cholangitis	PSC	2,871	12,019	9,270	27992413 (46)
Primary Sjogren's syndrome	SjS	3,232	17,481	10,911	35896530 (47)
Psoriasis (including Psoriatic arthritis)	PS (with PsA)	17,255	693,100	64,320	FinnGen (41), 34278373 (39), 24482804 (48); for details, see **Supplementary Table S2**
Rheumatoid arthritis	RA	22,350	74,823	68,838	36333501 (49)
Systemic lupus erythematosus	SLE	5,595	361,571	15,096	FinnGen (41), 26502338 (50), 29848360 (51); for details, see **Supplementary Table S2**
Systemic sclerosis	SS	9,095	17,584	23,978	31672989 (52)
Type 1 diabetes	T1D	18,942	501,638	73,011	34012112 (53)
Ulcerative colitis	UC	12,366	34,915	36,527	28067908 (40)

**Figure 2:**
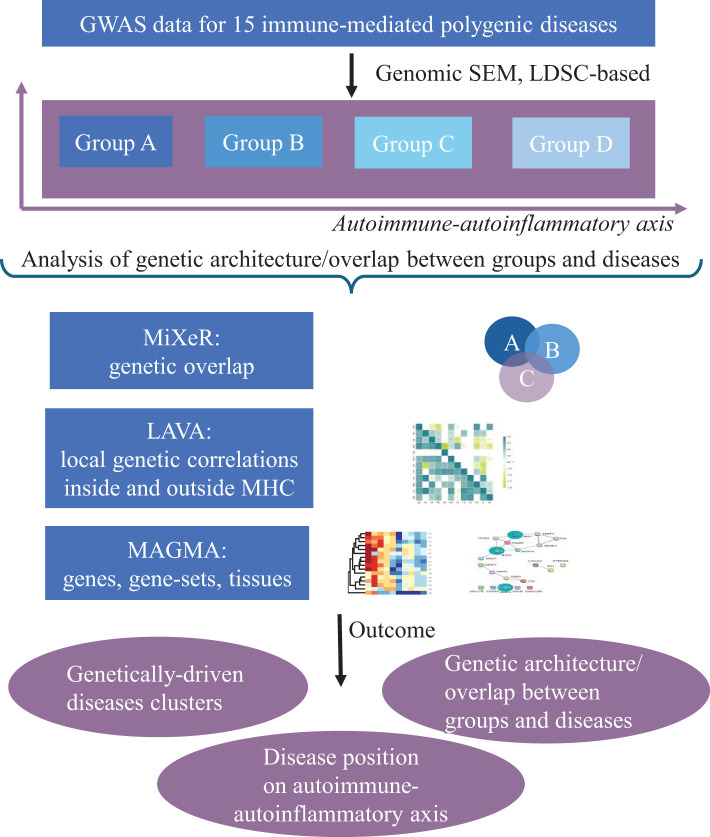
Schematic overview of the study design. The genomic structural equation model (SEM) was performed to cluster 15 immune-linked disorders on autoimmune–autoinflammatory axis. Linkage disequilibrium score regression (LDSR) was applied to study common genetic correlation between disorders. We did Local Analysis of [co]Variant Association (LAVA) to study local genetic correlations inside and outside MHC region. The causal mixture model (MiXeR), univariate, bivariate, and trivariate were applied to genetic overlap beyond global genetic correlation. Finally, MAGMA analysis was performed to reveal shared and unique gene, gene sets, and perform tissue analysis.

We started with 26 immune-linked disorders (**Supplementary Table S1**) with existing GWAS data. Those GWAS with an effective sample size >5,000 [based on recommendations (54, 55); N effective = 4/(1/n cases) + (1/n controls)] and >200,000 single-nucleotide polymorphisms (SNPs), which overlap with the linkage disequilibrium (LD) score regression reference panel (56) were included. We also excluded AS and narcolepsy because only ImmunoChip genetic data were available without genome-wide coverage. This resulted in the inclusion of 15 immune-mediated diseases for this study. To increase GWAS power for SLE, JIA, psoriasis (PS), and celiac disease (CeD), we performed in-house meta-analyses for each disorder across multiple publicly available GWAS datasets using METAL (**Table 1**; **Supplementary Table S2**). As well, we can highlight the need for more powerful GWAS datasets in immune-mediated neurological diseases because we can keep only two neurological diseases across whole dataset. All GWAS summary statistics were limited to participants of European ancestry. After data harmonization (conversion to GRCh37 genomic build) and pre-processing of the GWAS summary statistics with Python Convert (https://github.com/precimed/python_convert), we conducted cross-trait analyses using a variety of analytical tools as described below. The study design was shown at **Figure 2**

### 2.2 Analytical tools

#### 2.2.1 Genomic structural equation modeling

Genomic structural equation modeling (SEM) (57) can be used to model the multivariate genetic architecture across traits and reveal latent factors underlying genetic correlations and determine how those latent factors correlate with each other.

We conducted exploratory and confirmatory factor analyses (EFA and CFA, respectively) of the 15 immune-mediated diseases. First, the multivariable extension of LD score regression (LDSC) employed in genomic SEM was used to derive a genetic covariance matrix (S) and sampling covariance matrix (V). Population prevalence was taken from the literature (**Supplementary Table S3**). Next, EFA with promax rotation was conducted on the standardized S matrix using the R genomic SEM package (R version 4.3.2, https://github.com/GenomicSEM/, **Supplementary Table S4**). Results from the EFA were used to guide CFA for a one-, two-, three-, four-, and five-factor model. EFA was performed using genomic SEM, and correlated factors with standardized loadings >0.25 were retained for CFA. Model fit (**Table 2**; **Supplementary Table S5**) for each factor model was assessed using recommended fit indices: standardized root mean square residual (SRMR), model χ^2^ statistic, Akaike Information Criterion (AIC), and Comparative Fit Index (CFI). Model fit was considered acceptable for CFI values ≥ 0.90 and SRMR < 0.1 (57).

**Table 2:** Genomic SEM model performance criteria.

N factors	Df	χ^2^	*p-*value _χ2_	AIC	CFI	SRMR
1	90	1,321.83	4.56E−218	1,381.83	0.658	0.124
2	88	1,073.7	3.08E−169	1,137.69	0.727	0.111
3	87	779.35	9.09E−112	845.35	0.808	0.094
**4**	**56**	**215.59**	**1.42E−20**	**285.59**	**0.919**	**0.077**
5	54	238.37	5.25E−25	312.37	0.907	0.083

χ^2^, model chi-square, reflecting index of exact fit to observed data df; *p*-value _χ2_, degrees of freedom and p-value for the model χ^2^; AIC, Akaike Information Criterion; CFI, Comparative Fit Index; SRMR, standardized root mean square residual. Bold font was used for optimal 4-factor model.

#### 2.2.2 Linkage disequilibrium score regression and Gaussian causal mixture modeling (MiXeR)

For each phenotype, we estimated the SNP heritability and genome-wide genetic correlation using linkage disequilibrium score regression (LDSC) (54).

We applied MiXeR (58), (https://github.com/precimed/mixer) to pairs of phenotypes, to estimate the number of variants influencing both analyzed phenotypes (shared polygenicity) and the number of variants distinctively influencing each of the analyzed traits (trait-specific polygenicity). For MiXeR analyses, we excluded the MHC region 26–34 Mb, chromosome 6, as described (58) due to the intricate LD structure. For assessment of model robustness, delta AIC >0 and visual evaluation of log-likelihood plots were used. For the analysis of disease triplets, we used trivariate MiXeR (59), https://codeberg.org/intercm/mix3r with the possibility to access overlap between three phenotypes simultaneously.

#### 2.2.3 Local Analysis of [co]Variant Association

For local correlation analyses, we used Local Analysis of [co]Variant Association (LAVA) version 1.3.8, following the protocol with the LD reference panel based on 1000 Genomes phase 3 genotype data for European samples and the partition of the genome into 2,495 regions with an average size of 1 Mb as described elsewhere (60). Only regions revealing significant estimated SNP heritability (p<0.05/2,495) in both diseases were used to estimate local genetic correlations between the traits. We applied Bonferroni correction to account for multiple comparisons within each pairwise correlation analysis. The statistical tests conducted were all two-sided. Then, we identified 10 regions with the greatest number of significant local genetic correlations across immune traits and created network plots displaying these associations.

#### 2.2.4 Functional annotation

We applied MAGMA v1.08 with default parameters as implemented in FUMA v1.6.1 (61–63) to perform gene-level analysis for protein coding genes, gene-set analysis (17,023 gene sets from MsigDB v2023.1Hs), and tissue specificity analysis (54 different tissue types from GTEx eQTL v8) (61). We also analyzed genes revealed by MAGMA in each of the 15 diseases and common for all phenotypes and unique genes for diseases assigned to marginal latent factors obtained in genomic SEM analysis. Additionally, we analyzed unique gene sets from MAGMA results for genomic SEM factors. Bonferroni correction was used according to the number of comparisons. We used Cytoscape application Java 11.0.6 version 3.9.1 with default settings http://cytoscape.org, STRING app (64), and the STRING protein database (65) for protein–protein interaction visualization.

## 3 Results

### 3.1 Genomic SEM

**Figure 3:**
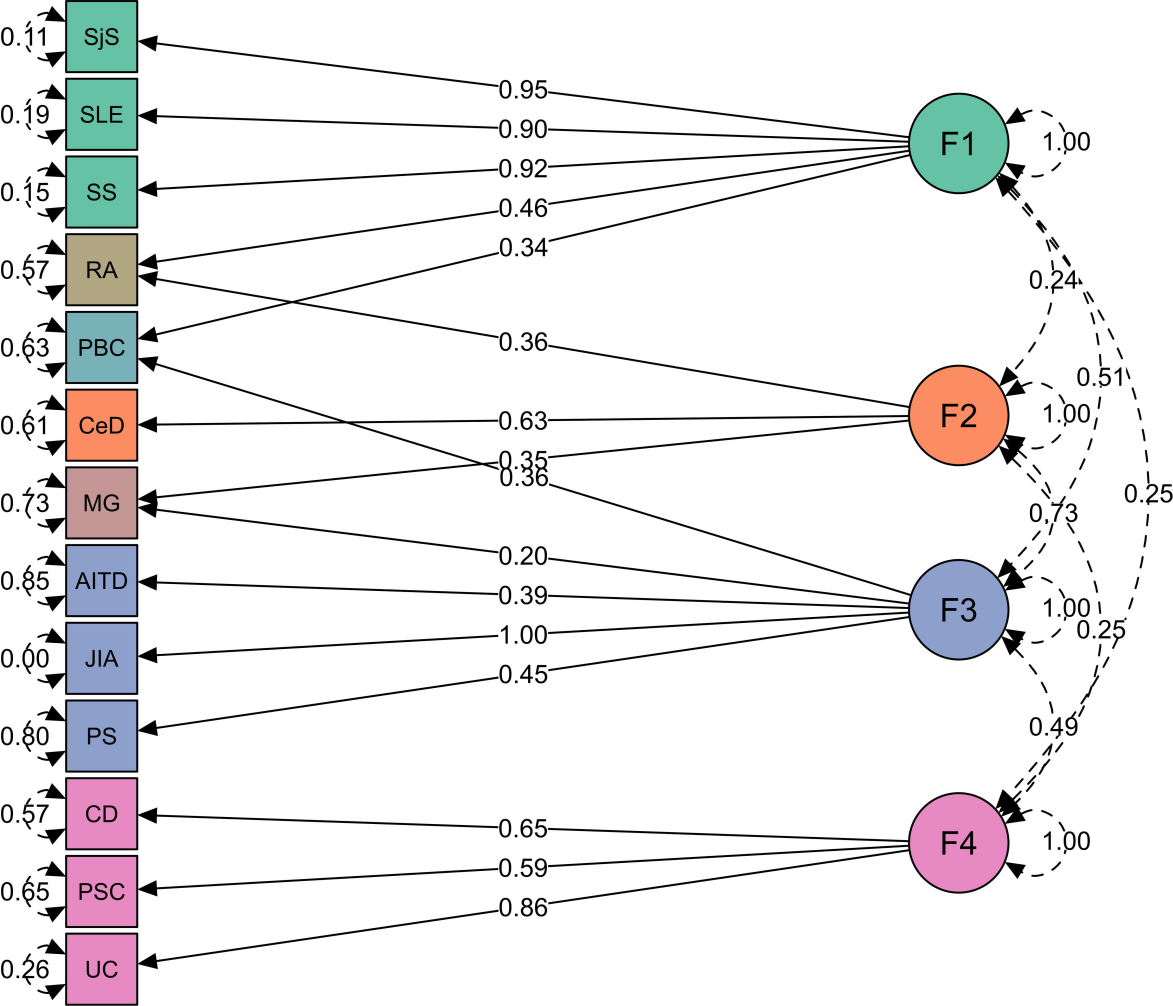
Four groups of immune-mediated diseases identified in genomic SEM analysis. Genomic SEM model with four latent factors representing four diseases clusters. Green represents factor 1 (F1, “autoimmune”), orange factor 2 (F2, “autoimmune-mixed”), blue factor 3 (F3, “mixed pattern”), purple factor 4 (F4, “autoinflammatory”), and diseases cross-load on two factors are shown in its own color. The arrows connecting the latent variables with diseases are shown as “factor loadings” obtained from confirmatory factor analysis. The rounded and dashed arrows on the indicators (traits) are residual variances in the genetic indicators not explained by the common factor. The dashed arrows connecting factors are covariances and give an idea about the factors’ mutual associations.

In our investigation of the genetic basis underlying the 15 immune-mediated diseases using genomic SEM, we sought to identify latent genetic factors shared across these traits. We employed multivariate LDSC as it implemented in genomic SEM to estimate genetic correlations, which informed our EFA. During CFA, we uncovered latent factors that represent shared variance components across diseases and modelled the genetic variance–covariance matrices across traits (**Figure 3**). This analysis identified a model with four latent factors as optimal among one-, two-, three-, four-, and five-factor model presented in **Table 2**, based on model performance criteria (see *Material and methods*). T1D and MS loaded on multiple factors using our predefined significance threshold (<0.25, see *Material and methods*), indicating diffuse association patterns that compromised the CFA model fit. Consequently, T1D and MS were excluded from the final model to maintain statistical robustness, as minor loadings led to reduced model performance. **Supplementary Table S4** showcases the EFA loadings of the four-factor model illustrating the non-specific loading patterns of T1D and MS.

Factor 1 consists of polygenic rheumatic diseases with autoimmune origin (SjS, SLE, and SS). Factor 2 consists of MG and CeD, but MG also loaded on factor 3. Factor 3 consists of AITD, JIA, and PS, which belong to a “mixed pattern” of pathologies between autoimmunity and autoinflammation. Factor 4 consists of diseases with gastrointestinal tract with inflammatory origin (CD, UC, and PSC). Several diseases cross-loaded on two factors, such as RA (equally belongs to factors 1 and 2), PBC (factors 1 and 3), and MG (factors 2 and 3; see **Figure 3**; **Supplementary Table S6**).

Here, we presented model performance criteria for the genomic SEM for a one-, two-, three-, four-, and five-factor model. The correlated factors with standardized loadings > 0.25 were retained from EFA for CFA. Model fit for each factor model was assessed using recommended fit indices: SRMR, model χ^2^ statistic, AIC, and CFI. Model fit was considered acceptable for CFI values ≥ 0.90 and SRMR < 0.1 (57).

### 3.2 LDSC and MiXeR

**Figure 4:**
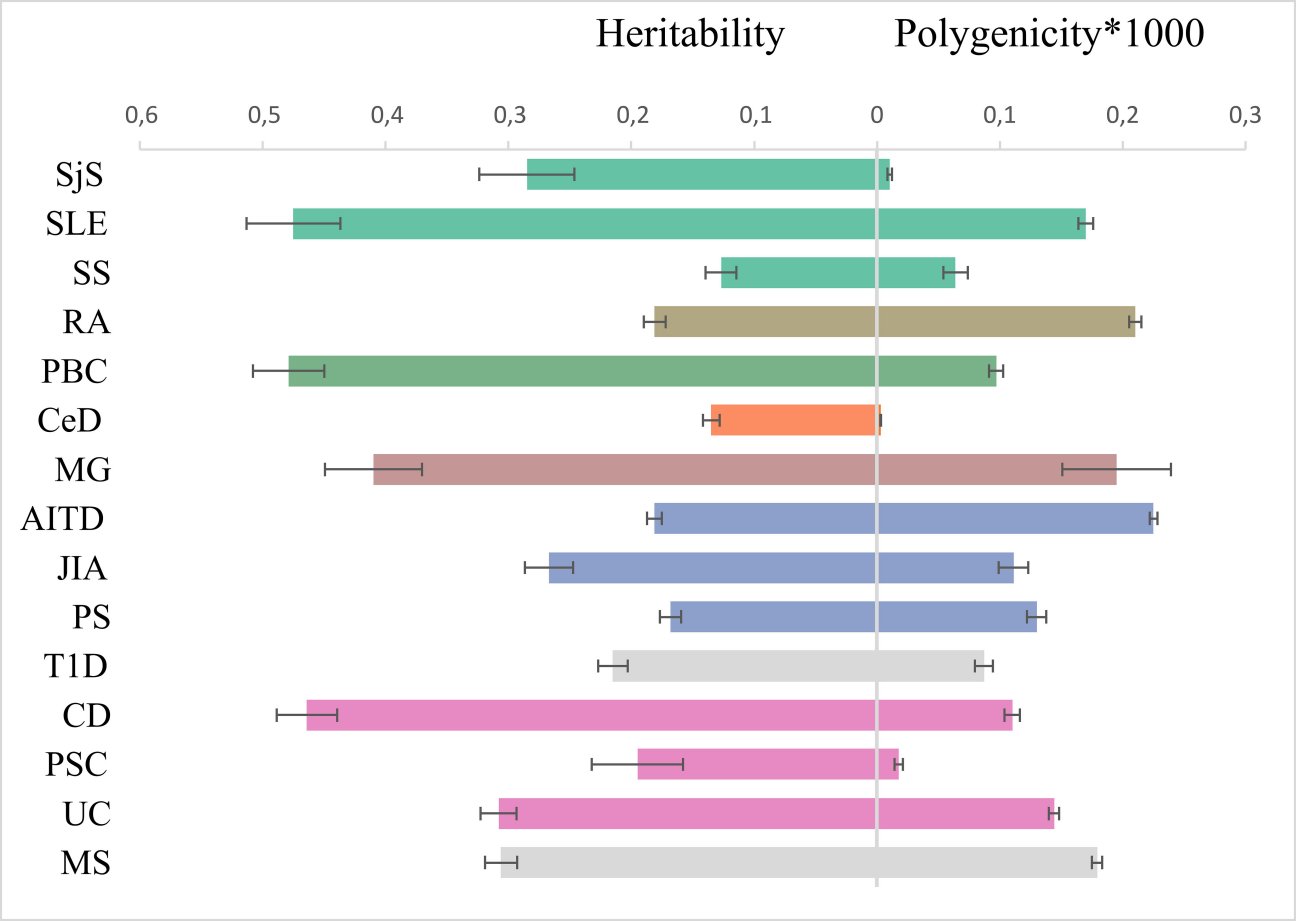
Genetic architecture characteristics. Left part: single-nucleotide polymorphism-based heritability, observed scale, for 15 immune-mediated diseases according to linkage disequilibrium score regression. Right part: the polygenicity value multiplied by 1000 according to Gaussian causal mixture modeling. The color of dots corresponds to color in genomic SEM figure (green factor 1, orange factor 2, blue factor 3, purple factor 4, and own color for mixed diseases).

We ran LDSC analysis (**Supplementary Figure S1**) to quantify SNP heritability, observed scale (**Figure 4**), and univariate and bivariate MiXeR for all phenotypes to characterize polygenicity and overlap between diseases.

We quantified SNP heritability using LDSC (**Supplementary Figure S1**) with the observed scale values depicted in **Figure 4**. CD, SLE, and PBC have a higher SNP heritability compared to others (>0.45).

**Figure 5:**
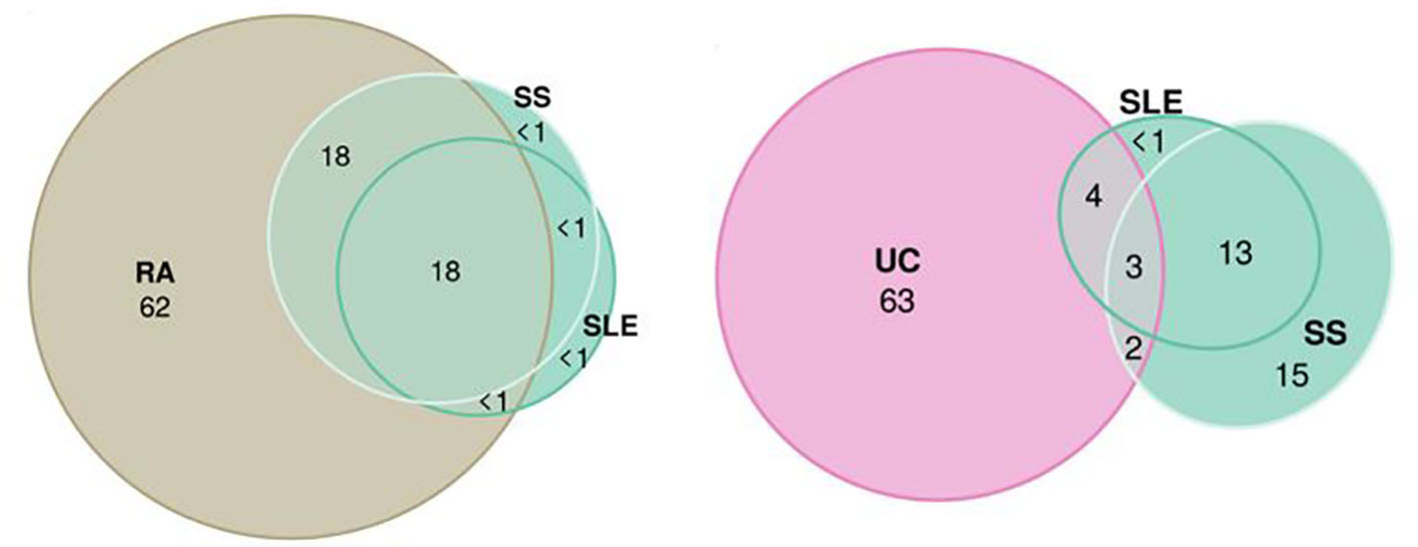
Trivariate Gaussian causal mixture modeling shows the overlap between factors. The illustrations of the overlap inside “autoimmune” factor 1 (systemic sclerosis, systemic lupus erythematosus, and rheumatoid arthritis) and between “autoimmune” factor 1 (systemic sclerosis and systemic lupus erythematosus) and “inflammatory” factor 4 (Ulcerative Colitis). For each triad of phenotypes, for every area of the diagram, its percentage is shown with respect to the combined total area of three phenotypes (rounded to the closest integer). The color of circles corresponds to the color in genomic SEM figure (green, F1; purple, F4; and beige, rheumatoid arthritis).

MiXeR results are presented in tables (**Supplementary Tables S7, S8**) and as a heatmap (**Supplementary Figure S2**) showing the proportion of trait-specific and shared trait-influencing SNPs followed by the standard deviation across 20 independent runs. Univariate MiXeR models exhibited good model fit (**Supplementary Table S7**). As shown in **Figure 4**, CD, SLE, and PBC have a higher observed scale SNP heritability compared to others (>0.45). CeD and SjS have the lowest polygenicity, and RA and AITD have the highest polygenicity. Bivariate results, for those diseases with acceptable model fit as it was evaluated by delta AIC criteria and log-likelihood profiles (58), see description in *Methods*) are presented in **Supplementary Figure S2**, **Supplementary Table S8**. Bivariate analysis for CeD, SjS, and PSC and as pairs of diseases including SLE-SS and RA-SS did not fulfil model robustness criteria and therefore are not presented. We run trivariate MiXeR for factor 1 and factor 4 clusters to show that diseases from the same factor have more overlap when compared to another factor. Trivariate analyses revealed that SLE/SS has less overlap with UC compared with RA (**Figure 5**).

### 3.3 LAVA across immune-mediated diseases

Local genetic correlation analyses complemented global genetic correlation between the immune-related phenotypes. This step allowed us to show which immune disorders genetic correlations are restricted to specific genomic regions and to identify the shared genetic factors located within these genomic regions. SLE, JIA, and MG were excluded, as their models failed to converge.

We visualized local genetic correlation across all loci (**Supplementary Figure S3**). During assessment of the pattern of intercorrelations (**Supplementary Figure S3**), we revealed that most of all diseases display a high degree of locus correlations, except CeD, which can be linked to small power of initial GWAS.

**Figure 6:**
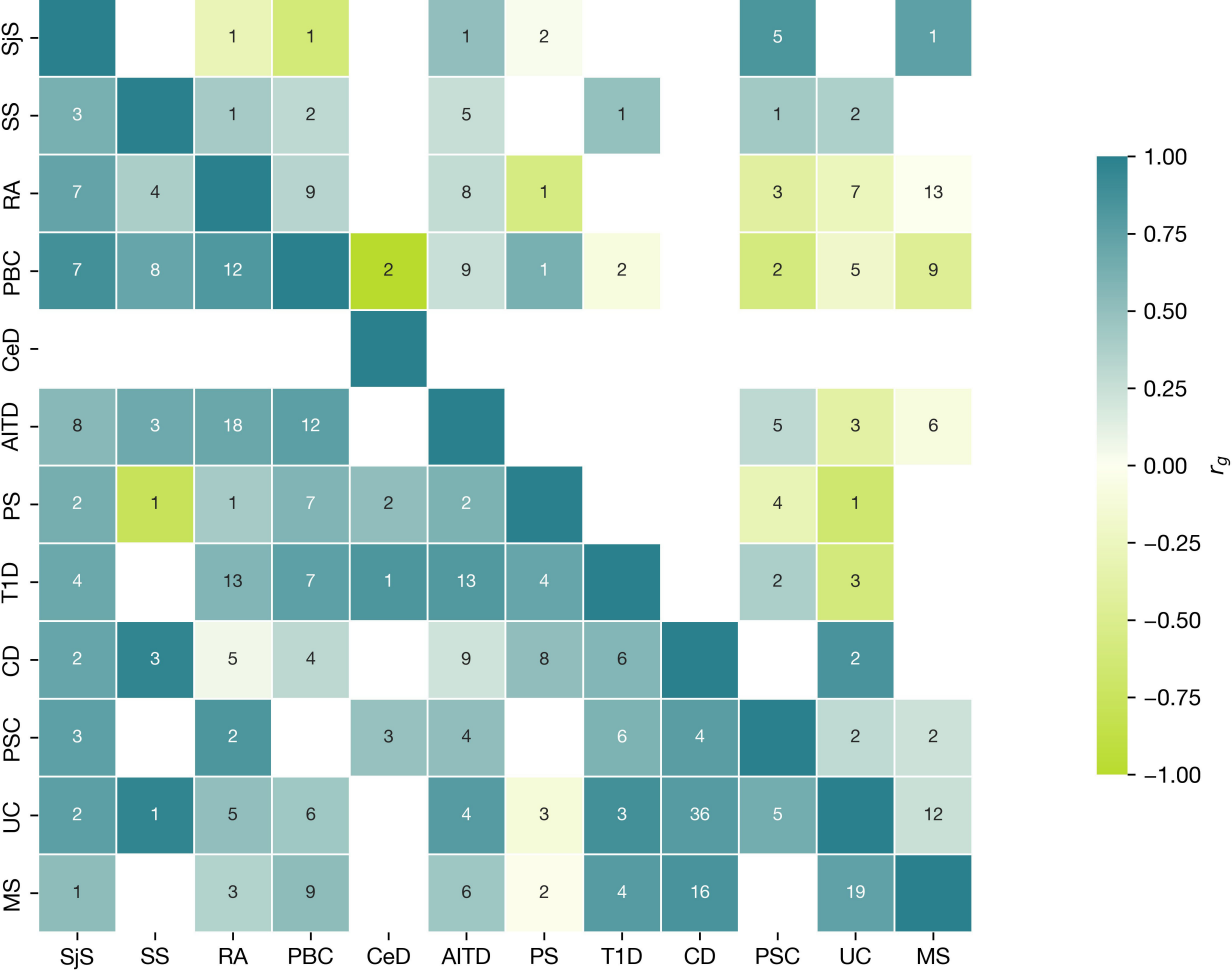
Local Analysis of [co]Variant Association (LAVA) characteristics for MHC locus. Correlation matrix of positive and negative significant correlation and number of loci within MHC (upper triangular part), with loci outside MHC (bottom triangular part). Numbers in cells show the number of regions with significant heritability in both traits (Bonferroni corrected), where the correlation was assessed. Colour indicates the direction of effect (aquamarine, positive; green, negative) for mean across all correlation values (r_g_s) within the regions with significant h2 for each phenotype. The correlation range is represented on the scale with a color gradient changing from −1 to 1.

In addition, we separately visualized the significant local genetic correlation inside MHC region (**Figure 6**) versus non-MHC loci. We confirmed that the local correlation and heritability were prominent in the MHC region, where strong genetic risk was shown. Among 20 regions with top significant local genetic correlation, 10 were in the MHC region with the top 1 being in HLA DRB1 gene, top 2 in HLA DQB1 (see **Supplementary Table S12** with figures). The pattern of local genetic correlation in the MHC region (**Figure 6**) involved a mixture of concordant and discordant effects across the diseases. For example, in loci on chromosome 6 (32539568–32586784), UC was negatively correlated with PBC, PSC, PS, AITD, and RA but positively correlated with MS. In comparison, fewer diseases displayed significant correlations in loci outside the MHC, but the correlations were generally strongly positive between the diseases (for example, chromosome 2: 191051955–193033982).

### 3.4 Functional annotation

#### 3.4.1 MAGMA shared results

MAGMA enrichment analyses for all 15 summary statistics revealed whole blood as the most enriched tissues for all diseases except PSC, SjS, and MG, and the spleen for all except PSC, SjS, and CeD. In addition, the small intestine terminal ileum, Cells_EBV-transformed_lymphocytes, and lung participated in 11, 9, and 8 diseases, respectively (**Supplementary Table S11**). We did not detect grouping of enriched tissues by factor.

We assessed the most frequently associated genes across traits: for 11 diseases follows (**Supplementary Figure S4**), *GABBR1* (gamma-aminobutyric acid type B receptor subunit 1), *ZKSCAN3* (Zinc Finger With KRAB And SCAN Domains 3), and *PGBD1* (PiggyBac Transposable Element-Derived 1), and for 10 diseases, *ZSCAN31* (Zinc Finger And SCAN Domain Containing 31), *SCAND3* (SCAN Domain Containing 3), and *ZSCAN23* (Zinc Finger And SCAN Domain Containing 23).

#### 3.4.2 Factor-specific MAGMA results for non-overlapping genes and gene sets

We also revealed genes only included in factor 1 or factor 4 (the most margin groups of autoimmune and autoinflammatory phenotypes, **Supplementary Table S9**). In factor 1, 22 genes were unique, and in factor 4, 159 genes were unique with some of them involved in monogenic immune diseases (see **Supplementary Figure S6** and *Discussion*).

Subsequently, we assessed gene sets and revealed the uniqueness of each factor. Nine unique gene sets were revealed for factor 1 (N02-mediated IL12 pathways, PID_TCR_pathway, and leukocytes and lymphocytes signaling), 16 unique gene sets for factor 3, 41 unique gene sets for factor 4, and 19 gene sets for MS and T1D (**Supplementary Table S10** and *Discussion*). There were no gene sets specific for factor 2 likely because factor 2 diseases shared many common genes with other factors.

## 4 Discussion

In this study of the common variant genetic architecture of diseases on the autoimmune and autoinflammatory continuum, we observed a strong convergence of shared genetic signal across different statistical genetics methods, which indicate clustering of immune-mediated disorders along the autoimmune-autoinflammatory continuum. Thus, we provide new evidence that supports recent call to action (33–36) on redefining the classification of immune-mediated diseases by integrating the genetics of polygenic immune disorders. We used state-of-the-art statistical methods to identify four clusters across the continuum from GWAS data, with strong autoimmune component in factor 1 and autoinflammatory component in factor 4. The extreme positions for factor 1 and factor 4 diseases were further supported by trivariate MiXeR. A significant overlap was observed within autoimmune diseases (SS, SLE, and RA), while there was a smaller overlap between factor 1 diseases (SLE and SS) and the factor 4 diseases (UC). In addition, we revealed a “mixed pattern” in factor 3, which includes JIA, PS, and AITD inside cluster, while factor 2 consists of MG and CeD. MG and CeD diseases are confirmed to be autoimmune disorders (12), but genetically, MG also shares components with factor 3, which belongs to mixed pathology. Therefore, we proposed naming this cluster “autoimmune-mixed”.

The classification of UC, CD, and PSC in the same cluster (factor 4) is consistent with a previous study (30) that utilized genomic SEM to demonstrate pathway convergence in immune disorders. While authors (30) suggest that this factor corresponds to gastric disorders, we show that other gastroenterological phenotypes (PBC and CeD) cluster with different diseases. Therefore, this grouping may not solely be due to the involvement of the gastroenterological tract but may suggest grouping based on inflammatory pathogenesis. Additionally, it is noteworthy that disorders affecting the same organ can be classified into different groups, highlighting the complexity of their immune mechanisms. Topaloudi et al. (32) also revealed four factors structure in line with a recent preprint (16). The study found that MS was grouped with PSC (32), and our study confirmed this connection using LDSC and LAVA methods. Similarly, MG was grouped with RA (32), and we were able to show that they belong partly to the same factor 2. In the Transimmunome project (36, 66), five factors were identified based on molecular immunophenotyping but with inclusion of additional diseases with monogenic inheritance or diseases lacking robust GWAS data. Furthermore, our factor 3 consists of diseases that were not studied in their project. Tchitchek et al. grouped CD and UC into the same factor (factor 4) supporting the findings on biomarkers level. In addition, RA and T1D were grouped together, which aligns with our large genetic overlap observed in MiXeR (rg 0.33, shared fraction 38%) and strong local positive correlations in some loci as indicated by LAVA.

In addition, in our study, SLE belongs to the “autoimmune” factor 1, while RA is split between factors 1 and 2, indicating distinct genetic backgrounds for RA and SLE.

SLE is regarded as a classical autoimmune disease with autoantibody production, whereas RA has both autoimmune (classical seropositive RA) and mixed/autoinflammatory features (seronegative subtypes) (11). A notable observation in our study is the grouping of MG with CeD (in factor 2), which may explain their co-occurrence in case reports, despite lacking population-level evidence (67). While MS was excluded from our genomic SEM model, it shows high correlation and overlap with factor 4 diseases and PBC using other methods. This was shown in previous studies (67, 68), which support the observation that MS has common genetic background not only with autoimmune but also with autoinflammatory clusters or, as it was suggested recently, for functionally integrated immunopathology (69).

Based on our findings and previous literature (30, 32, 70), it appears that the factor structure in the genomic SEM model depends on 1) the initial set of disorders and 2) GWAS power. By including more diseases in our analyses compared to the previous studies, we were able to differentiate more factors within the autoimmune spectrum. In summary, our results align with and expand previous findings by including a broader range of immune-mediated diseases, which enabled us to define more refined clusters on the “autoimmune-autoinflammatory axis”.

LAVA analysis showed a mixed pattern of correlations (positive and negative) inside the MHC region, but in loci outside, the MHC region correlations were mostly positive. The same pattern for mixed correlation in the main MHC hotspot (chromosome 6: 32539568–32586784) was shown for immune-mediated diseases in the original LAVA paper (60). Most MHC loci with significant local correlation belong to the MHC II subgroup. In contrast, PS categorized as an MHC-1-opathy according to the European Alliance of Associations for Rheumatology classification (71, 72) generally shows a negative correlation with other diseases in top-MHC loci. Considering the development of demyelinating disorders in some MHC-I-opathies during biological treatment (28), this insight can guide research on more informed treatment choices and help prevent complications arising from impacts on various pathways.

The most frequently identified overlapping genes (**Supplementary Figure S4**) among the immune-mediated disorders were involved in cell signaling and interaction, which is common for all types of immune disorders and consistent with findings from a previous study (31). We also assessed the unique genes in marginal “autoimmune” and “autoinflammatory” factors. In “autoimmune” factor (factor 1), 22 genes were unique, among them neutrophil cytosolic factor 2, which is associated with autosomal recessive chronic granulomatous disease 2 (73). Mutation in Interferon Regulatory Factor 7 was described as a cause of immunodeficiency-39 (73) and as a gene, which is linked to innate immunity according to the classification of inborn errors of immunity (74). Mutation in *IKZF3* is linked to immunodeficiency-84, which is an autosomal dominant primary immunologic disease associated with low levels of B cells and impaired early B-cell development (75).

For “autoinflammatory” factor 4, 159 genes were unique, but few participated in immunological processes (**Supplementary Figure S6**). One immune-related example is *NOD2*, a gene involved in Blau syndrome, which is a rare autosomal dominant autoinflammatory syndrome classified as an autoinflammatory phenotype (74). These findings are in line with (34), which reports that monogenic immune-mediated diseases may represent genetically determined, more severe forms of more common polygenic autoimmune and inflammatory diseases. That also enables combining results from rare and complex diseases, which can potentially inform new strategies for more precise treatment selection. Based on these findings, we can explore certain potential agents in greater depth. If these agents prove effective for Blau syndrome, there is a possibility that they could also benefit other disorders within the group (76, 77).

In the assessment of gene sets, we identified the involvement of interleukin 12 (IL12) and IL23 gene sets in factor 4 diseases. A monoclonal antibody that targets IL12/IL23 (ustekinumab) is approved for the treatment of UC and CD, and PS (78–80). In addition, IL3, IL4, IL18, IL27, T-helper, and IFN gene sets involvement in “inflammatory” factor 4 can inform the potential application of drugs that targets these interleukins and related pathways.

For “autoimmune” factor 1, both N02-mediated IL12 pathways and the IL12 pathway were involved as previously linked to SLE risk (81). In addition, TCR signaling in naive CD4+ T cells and leukocyte-associated pathways participated in factor 1 diseases. This can be important for SjS and SS where fewer IL pathways-linked studies exist due to the lack of powerful data (82).

In factor 3 diseases, a lot of pathways were enriched that were linked to T cells, B cells, and JAK signaling, and IL17. Recent studies have shown the involvement of IL17 in JIA pathogenesis (83). This has led to the approval of secukinumab, an IL 17 blocker, for treating two JIA subtypes (juvenile psoriatic arthritis and enthesitis-related arthritis) and for PS treatment. In addition, for MS and T1D, different IL2, IL6, and T-cell pathways were important.

Our approach offers significant advancements in understanding the intricate genetics of immune-mediated disorders, and its translational potential could be significant. The accumulated evidence from this study and previous findings can help inform further research on immunotherapy in immune-mediated disorders. Already designed drugs that suppress major pro-inflammatory signaling pathways as IL-17 and JAK inhibitors have success compared to traditional systemic therapies (84). However, there are still unaddressed medical requirements in terms of both long-term safety and overall effectiveness, as a considerable number of patients do not attain disease remission. Enhanced understanding of the genetically informed mechanisms and diversity within immune-mediated diseases would create opportunities to address these challenges, resulting in a more personalized and efficient treatment approach (85).

A limitation of our study is that it focused only on GWAS performed on populations of European ancestry. This is because genomic SEM and LD score regression require the same ancestry samples, and powerful GWAS data are lacking for non-European ancestries in most diseases. The second limitation was that only powerful GWAS datasets of polygenic diseases were included, a requirement of the employed statistical methods. In addition, cohort sample size and SNP sets are drivers of the limited resolution of genetic mapping and the ability to detect robust disease associations (86). The MHC findings could be limited by non-specialized genotyping chips in GWAS and resolution of the MHC region, and limitations of the LAVA method due to LD structure and GWAS power. An additional limitation was to focus only on genetics but not on the deep immunophenotyping due to the lack of powerful data for these phenotypes.

To summarize, the revelation of four-factor clustering can help to define the disease groups for which it is possible to use the same therapies. The shared genetic landscape of this continuum can potentially be used to update the nosological classification. Our findings should be viewed as a foundation for further investigation into personalized treatment strategies based on genetic clustering. We believe that these insights could eventually contribute to optimizing therapeutic approaches, but rigorous clinical validation is necessary before any changes to existing treatments can be recommended. Furthermore, our results can be instrumental in assembling cluster-guided multi-parametric analyses that include genetics and omics data and enable deep phenotyping of patients leading to personalized drug selection. The current study sheds light on the autoimmune–autoinflammatory continuum from a genetic perspective and can inform future studies in this field.

## Data Availability

GWAS data are publicly available at GWAS catalogue or available on request (International Multiple Sclerosis Genetic Consortium, deCODE, FinnGen). Statistical analyses were performed with Python and R using existing tools available in FUMA (https://fuma.ctglab.nl/), GitHub (https://github.com/precimed/). Genomic SEM (https://github.com/GenomicSEM/), METAL (https://github.com/statgen/METAL), MiXeR bivariate (https://github.com/precimed/mixer), MiXeR trivariate (https://codeberg.org/intercm/mix3r), LAVA (https://github.com/josefin-werme/LAVA), LDSC (https://github.com/bulik/ldsc), MAGMA (https://cncr.nl/research/magma/), and Python Convert (https://github.com/precimed/python_convert).

## References

[1] Dalmau J Graus F. Antibody-mediated encephalitis. N Engl J Med. (2018) 378:840–51. doi: 10.1056/NEJMra1708712 29490181

[2] Canna SW Goldbach-Mansky R. New monogenic autoinflammatory diseases–a clinical overview. Semin Immunopathol. (2015) 37:387–94. doi: 10.1007/s00281-015-0493-5 PMC4556430 25963521

[3] Di Donato G d’Angelo DM Breda L Chiarelli F. Monogenic autoinflammatory diseases: state of the art and future perspectives. Int J Mol Sci. (2021) 22:6360. doi: 10.3390/ijms22126360 34198614 PMC8232320

[4] de Jesus AA Chen G Yang D Brdicka T Ruth NM Bennin D. Constitutively active Lyn kinase causes a cutaneous small vessel vasculitis and liver fibrosis syndrome. Nat Commun. (2023) 14(1):1502. doi: 10.1038/s41467-023-36941-y 36932076 PMC10022554

[5] Conrad N Misra S Verbakel JY Verbeke G Molenberghs G Taylor PN. Incidence, prevalence, and co-occurrence of autoimmune disorders over time and by age, sex, and socioeconomic status: a population-based cohort study of 22 million individuals in the UK. Lancet. (2023) 401:1878–90. doi: 10.1016/S0140-6736(23)00457-9 37156255

[6] Miller FW. The increasing prevalence of autoimmunity and autoimmune diseases: an urgent call to action for improved understanding, diagnosis, treatment, and prevention. Curr Opin Immunol. (2023) 80:102266. doi: 10.1016/j.coi.2022.102266 36446151 PMC9918670

[7] Held F Klein AK Berthele A. Drug treatment of neuromyelitis optica spectrum disorders: out with the old, in with the new? Immunotargets Ther. (2021) 10:87–101. doi: 10.2147/ITT.S287652 33777853 PMC7989551

[8] DeHart-McCoyle M Patel S Du X. New and emerging treatments for myasthenia gravis. BMJ Med. (2023) 2:e000241. doi: 10.1136/bmjmed-2022-000241 PMC10407383 37560511

[9] Perez E. Future of therapy for inborn errors of immunity. Clin Rev Allergy Immunol. (2022) 63:75–89. doi: 10.1007/s12016-021-08916-8 35020169 PMC8753954

[10] Savic S Caseley EA McDermott MF. Moving towards a systems-based classification of innate immune-mediated diseases. Nat Rev Rheumatol. (2020) 16:222–37. doi: 10.1038/s41584-020-0377-5 32107482

[11] Szekanecz Z McInnes IB Schett G Szamosi S Benkő S Szűcs G. Autoinflammation and autoimmunity across rheumatic and musculoskeletal diseases. Nat Rev Rheumatol. (2021) 17:585–95. doi: 10.1038/s41584-021-00652-9 34341562

[12] McGonagle D McDermott MF. A proposed classification of the immunological diseases. PloS Med. (2006) 3:e297. doi: 10.1371/journal.pmed.0030297 16942393 PMC1564298

[13] Matzinger P. Autoimmunity: Are we asking the right question? Front Immunol. (2022) 13:864633. doi: 10.3389/fimmu.2022.864633 36405714 PMC9671104

[14] Shirafkan F Hensel L Rattay K. Immune tolerance and the prevention of autoimmune diseases essentially depend on thymic tissue homeostasis. Front Immunol. (2024) 15:1339714. doi: 10.3389/fimmu.2024.1339714 38571951 PMC10987875

[15] Ben-Chetrit E Gattorno M Gul A Kastner DL Lachmann HJ Touitou I. Paediatric Rheumatology International Trials Organisation (PRINTO) and the AIDs Delphi study participants. Consensus proposal for taxonomy and definition of the autoinflammatory diseases (AIDs): a Delphi study. Ann Rheum Dis. (2018) 77:1558–65. doi: 10.1136/annrheumdis-2017-212515 30100561

[16] Breunig S Lee YH Karlson EW Krishnan A Lawrence JM Schaffer LS. Examining the genetic links between clusters of immune-mediated diseases and psychiatric disorders. medRxiv. (2024), 24310651. doi: 10.1101/2024.07.18.24310651 PMC12279975 40691433

[17] Mauro D Thomas R Guggino G Lories R Brown MA Ciccia F. Ankylosing spondylitis: an autoimmune or autoinflammatory disease? Nat Rev Rheumatol. (2021) 17:387–404. doi: 10.1038/s41584-021-00625-y 34113018

[18] Fichtner ML Jiang R Bourke A Nowak RJ O’Connor KC. Autoimmune pathology in myasthenia gravis disease subtypes is governed by divergent mechanisms of immunopathology. Front Immunol. (2020) 11:776. doi: 10.3389/fimmu.2020.00776 32547535 PMC7274207

[19] Binks SNM Klein CJ Waters P Pittock SJ Irani SR. LGI1, CASPR2 and related antibodies: a molecular evolution of the phenotypes. J Neurol Neurosurg Psychiatry. (2018) 89:526–34. doi: 10.1136/jnnp-2017-315720 PMC5909759 29055902

[20] de Moraes MPM do Nascimento RRNR Abrantes FF Pedroso JL Perazzio SF Barsottini OGP. What general neurologists should know about autoinflammatory syndromes? Brain Sci. (2023) 13:1351. doi: 10.3390/brainsci13091351 37759952 PMC10526530

[21] Höftberger R Lassmann H. Inflammatory demyelinating diseases of the central nervous system. Handb Clin Neurol. (2017) 145:263–83. doi: 10.1016/B978-0-12-802395-2.00019-5 PMC7149979 28987175

[22] Grateau G Hentgen V Stojanovic KS Jéru I Amselem S Steichen O. How should we approach classification of autoinflammatory diseases? Nat Rev Rheumatol. (2013) 9:624–9. doi: 10.1038/nrrheum.2013.101 23838615

[23] Pathak S McDermott MF Savic S. Autoinflammatory diseases: update on classification diagnosis and management. J Clin Pathol. (2017) 70:1–8. doi: 10.1136/jclinpath-2016-203810 27646526

[24] Harroud A Hafler DA. Common genetic factors among autoimmune diseases. Science. (2023) 380:485–90. doi: 10.1126/science.adg2992 PMC13069990 37141355

[25] Barkhane Z Elmadi J Satish Kumar L Pugalenthi LS Ahmad M Reddy S. Multiple sclerosis and autoimmunity: A veiled relationship. Cureus. (2022) 14:e24294. doi: 10.7759/cureus.24294 35607574 PMC9123335

[26] Gerussi A Soskic B Asselta R Invernizzi P Gershwin ME. GWAS and autoimmunity: What have we learned and what next. J Autoimmun. (2022) 133:102922. doi: 10.1016/j.jaut.2022.102922 36209690

[27] Remalante-Rayco P Espiritu AI Daghistani Y Chim T Atenafu E Keshavarzi S. Incidence and predictors of demyelinating disease in spondyloarthritis: data from a longitudinal cohort study. Rheumatol (Oxford). (2023) 4:kead527. doi: 10.1093/rheumatology/kead527 37792508

[28] Fominykh V Shevtsova T Arzumanian N Brylev L. Coexistence of multiple sclerosis and ankylosing spondylitis: Report of four cases from Russia and review of the literature. J Clin Neurosci. (2017) 44:230–3. doi: 10.1016/j.jocn.2017.06.031 28684154

[29] Sakaue S Kanai M Tanigawa Y Karjalainen J Kurki M Koshiba S. A cross-population atlas of genetic associations for 220 human phenotypes. Nat Genet. (2021) 53:1415–24. doi: 10.1038/s41588-021-00931-x PMC12208603 34594039

[30] Demela P Pirastu N Soskic B. Cross-disorder genetic analysis of immune diseases reveals distinct gene associations that converge on common pathways. Nat Commun. (2023) 14:2743. doi: 10.1038/s41467-023-38389-6 37173304 PMC10182075

[31] Lincoln MR Connally N Axisa PP Gasperi C Mitrovic M van Heel D. Genetic mapping across autoimmune diseases reveals shared associations and mechanisms. Nat Genet. (2024) 56(5):838–45. doi: 10.1038/s41588-024-01732-8 PMC13095137 38741015

[32] Topaloudi A Jain P Martinez MB Bryant JK Reynolds G Zagoriti Z. PheWAS and cross-disorder analysis reveal genetic architecture, pleiotropic loci and phenotypic correlations across 11 autoimmune disorders. Front Immunol. (2023) 14:1147573. doi: 10.3389/fimmu.2023.1147573 37809097 PMC10552152

[33] Bousfiha A Jeddane L Picard C Al-Herz W Ailal F Chatila T. Human inborn errors of immunity: 2019 update of the IUIS phenotypical classification. J Clin Immunol. (2020) 40:66–81. doi: 10.1007/s10875-020-00758-x 32048120 PMC7082388

[34] Guthrie J Köstel Bal S Lombardo SD Müller F Sin C Hütter CVR. AutoCore: A network-based definition of the core module of human autoimmunity and autoinflammation. Sci Adv. (2023) 9:eadg6375. doi: 10.1126/sciadv.adg6375 37656781 PMC10848965

[35] Lorenzon R Mariotti-Ferrandiz E Aheng C Ribet C Toumi F Pitoiset F. Clinical and multi-omics cross-phenotyping of patients with autoimmune and autoinflammatory diseases: the observational TRANSIMMUNOM protocol. BMJ Open. (2018) 8:e021037. doi: 10.1136/bmjopen-2017-021037 PMC6119447 30166293

[36] Tchitchek N Binvignat M Roux A Pitoiset F Dubois J Marguerit G. Deep immunophenotyping reveals that autoimmune and autoinflammatory disorders are spread along two immunological axes capturing disease inflammation levels and types. Ann Rheum Dis. (2024) 83(5):638–50. doi: 10.1136/ard-2023-225179 PMC11041612 38182406

[37] Saevarsdottir S Olafsdottir TA Ivarsdottir EV Halldorsson GH Gunnarsdottir K Sigurdsson A. FLT3 stop mutation increases FLT3 ligand level and risk of autoimmune thyroid disease. Nature. (2020) 584:619–23. doi: 10.1038/s41586-020-2436-0 32581359

[38] Dubois PC Trynka G Franke L Hunt KA Romanos J Curtotti A. Multiple common variants for celiac disease influencing immune gene expression. Nat Genet. (2010) 42:295–302. doi: 10.1038/ng.543 20190752 PMC2847618

[39] Glanville KP Coleman JRI O’Reilly PF Galloway J Lewis CM. Investigating pleiotropy between depression and autoimmune diseases using the UK biobank. Biol Psychiatry Glob Open Sci. (2021) 1:48–58. doi: 10.1016/j.bpsgos.2021.03.002 34278373 PMC8262258

[40] de Lange KM Moutsianas L Lee JC Lamb CA Luo Y Kennedy NA. Genome-wide association study implicates immune activation of multiple integrin genes in inflammatory bowel disease. Nat Genet. (2017) 49:256–61. doi: 10.1038/ng.3760 PMC5289481 28067908

[41] Kurki MI Karjalainen J Palta P Sipilä TP Kristiansson K Donner KM. FinnGen provides genetic insights from a well-phenotyped isolated population. Nature. (2023) 613:508–18. doi: 10.1038/s41586-022-05473-8 PMC9849126 36653562

[42] López-Isac E Smith SL Marion MC Wood A Sudman M Yarwood A. Combined genetic analysis of juvenile idiopathic arthritis clinical subtypes identifies novel risk loci, target genes and key regulatory mechanisms. Ann Rheum Dis. (2021) 80:321–8. doi: 10.1136/annrheumdis-2020-218481 PMC7892389 33106285

[43] International Multiple Sclerosis Genetics Consortium. Multiple sclerosis genomic map implicates peripheral immune cells and microglia in susceptibility. Science. (2019) 365:eaav7188. doi: 10.1126/science.aav7188 31604244 PMC7241648

[44] Chia R Saez-Atienzar S Murphy N Chiò A Blauwendraat C International Myasthenia Gravis Genomics Consortium. Identification of genetic risk loci and prioritization of genes and pathways for myasthenia gravis: a genome-wide association study. Proc Natl Acad Sci U S A. (2022) 119:e2108672119. doi: 10.1073/pnas.2108672119 35074870 PMC8812681

[45] Cordell HJ Fryett JJ Ueno K Darlay R Aiba Y Hitomi Y. An international genome-wide meta-analysis of primary biliary cholangitis: Novel risk loci and candidate drugs. J Hepatol. (2021) 75:572–81. doi: 10.1016/j.jhep.2021.04.055 PMC8811537 34033851

[46] Ji SG Juran BD Mucha S Folseraas T Jostins L Melum E. Genome-wide association study of primary sclerosing cholangitis identifies new risk loci and quantifies the genetic relationship with inflammatory bowel disease. Nat Genet. (2017) 49:269–73. doi: 10.1038/ng.3745 PMC5540332 27992413

[47] Khatri B Tessneer KL Rasmussen A Aghakhanian F Reksten TR Adler A. Genome-wide association study identifies Sjögren’s risk loci with functional implications in immune and glandular cells. Nat Commun. (2022) 13:4287. doi: 10.1038/s41467-022-30773-y 35896530 PMC9329286

[48] Ellinghaus D Ellinghaus E Nair RP Stuart PE Esko T Metspalu A. Combined analysis of genome-wide association studies for Crohn disease and psoriasis identifies seven shared susceptibility loci. Am J Hum Genet. (2012) 90:636–47. doi: 10.1016/j.ajhg.2012.02.020 PMC3322238 22482804

[49] Ishigaki K Sakaue S Terao C Luo Y Sonehara K Yamaguchi K. Multi-ancestry genome-wide association analyses identify novel genetic mechanisms in rheumatoid arthritis. Nat Genet. (2022) 54:1640–51. doi: 10.1038/s41588-022-01213-w PMC10165422 36333501

[50] Bentham J Morris DL Graham DSC Pinder CL Tombleson P Behrens TW. Genetic association analyses implicate aberrant regulation of innate and adaptive immunity genes in the pathogenesis of systemic lupus erythematosus. Nat Genet. (2015) 47:1457–64. doi: 10.1038/ng.3434 PMC4668589 26502338

[51] Julià A López-Longo FJ Pérez Venegas JJ Bonàs-Guarch S Olivé À Andreu JL. Genome-wide association study meta-analysis identifies five new loci for systemic lupus erythematosus. Arthritis Res Ther. (2018) 20:100. doi: 10.1186/s13075-018-1604-1 29848360 PMC5977506

[52] López-Isac E Acosta-Herrera M Kerick M Assassi S Satpathy AT Granja J. GWAS for systemic sclerosis identifies multiple risk loci and highlights fibrotic and vasculopathy pathways. Nat Commun. (2019) 10:4955. doi: 10.1038/s41467-019-12760-y 31672989 PMC6823490

[53] Chiou J Geusz RJ Okino ML Han JY Miller M Melton R. Interpreting type 1 diabetes risk with genetics and single-cell epigenomics. Nature. (2021) 594:398–402. doi: 10.1038/s41586-021-03552-w 34012112 PMC10560508

[54] Bulik-Sullivan BK Loh PR Finucane HK Ripke S Yang J. LD Score regression distinguishes confounding from polygenicity in genome-wide association studies. Nat Genet. (2015) 47:291–5. doi: 10.1038/ng.3211 PMC4495769 25642630

[55] Tylee DS Sun J Hess JL Tahir MA Sharma E Malik R Worrall BB Research Team; Inflammation Working Group of the CHARGE Consortium METASTROKE Consortium of the International Stroke Genetics Consortium Netherlands Twin Registry neuroCHARGE Working Group Obsessive Compulsive and Tourette Syndrome Working Group of the Psychiatric Genomics Consortium Faraone SV Glatt SJ. Genetic correlations among psychiatric and immune-related phenotypes based on genome-wide association data. Am J Med Genet B Neuropsychiatr Genet. (2018) 177(7):641–57. doi: 10.1002/ajmg.b.32652 PMC6230304 30325587

[56] Zheng J Erzurumluoglu AM Elsworth BL Kemp JP Howe L Haycock PC. LD Hub: a centralized database and web interface to perform LD score regression that maximizes the potential of summary level GWAS data for SNP heritability and genetic correlation analysis. Bioinformatics. (2017) 33:272–9. doi: 10.1093/bioinformatics/btw613 PMC5542030 27663502

[57] Grotzinger AD Rhemtulla M de Vlaming R Ritchie SJ Mallard TT Hill WD. Genomic structural equation modelling provides insights into the multivariate genetic architecture of complex traits. Nat Hum Behav. (2019) 3:513–25. doi: 10.1038/s41562-019-0566-x PMC6520146 30962613

[58] Frei O Holland D Smeland OB Shadrin AA Fan CC Maeland S. Bivariate causal mixture model quantifies polygenic overlap between complex traits beyond genetic correlation. Nat Commun. (2019) 10:2417. doi: 10.1038/s41467-019-10310-0 31160569 PMC6547727

[59] Shadrin AA Hindley G Hagen E Parker N Tesfaye M Jaholkowski P. Dissecting the genetic overlap between three complex phenotypes with trivariate MiXeR. medRxiv. (2024), 24303236. doi: 10.1101/2024.02.23.24303236

[60] Werme J van der Sluis S Posthuma D de Leeuw CA. An integrated framework for local genetic correlation analysis. Nat Genet. (2022) 54:274–82. doi: 10.1038/s41588-022-01017-y 35288712

[61] Watanabe K Taskesen E van Bochoven A Posthuma D. Functional mapping and annotation of genetic associations with FUMA. Nat Commun. (2017) 8:1826. doi: 10.1038/s41467-017-01261-5 29184056 PMC5705698

[62] de Leeuw CA Mooij JM Heskes T Posthuma D. MAGMA: generalized gene-set analysis of GWAS data. PloS Comput Biol. (2015) 11:e1004219. doi: 10.1371/journal.pcbi.1004219 25885710 PMC4401657

[63] 1000 Genomes Project Consortium Auton A Brooks LD Durbin RM Garrison EP Kang HM. A global reference for human genetic variation. Nature. (2015) 526:68–74. doi: 10.1038/nature15393 26432245 PMC4750478

[64] Shannon P Markiel A Ozier O Baliga NS Wang JT Ramage D. Cytoscape: a software environment for integrated models of biomolecular interaction networks. Genome Res. (2003) 13:2498–504. doi: 10.1101/gr.1239303 PMC403769 14597658

[65] Szklarczyk D Morris JH Cook H Kuhn M Wyder S Simonovic M. The STRING database in 2017: quality-controlled protein-protein association networks, made broadly accessible. Nucleic Acids Res. (2017) 45:D362–8. doi: 10.1093/nar/gkw937 PMC5210637 27924014

[66] Hässler S Lorenzon R Binvignat M Ribet C Roux A Johanet C. Clinical correlates of lifetime and current comorbidity patterns in autoimmune and inflammatory diseases. J Autoimmun. (2024) 149:103318. doi: 10.1016/j.jaut.2024.103318 39357469

[67] Thawani SP Brannagan TH Lebwohl B Green PHR Ludvigsson JF. Celiac disease and risk of myasthenia gravis - nationwide population-based study. BMC Neurol. (2018) 18:28. doi: 10.1186/s12883-018-1035-2 29529996 PMC5848580

[68] Kim W Patsopoulos NA. Genetics and functional genomics of multiple sclerosis. Semin Immunopathol. (2022) 44:63–79. doi: 10.1007/s00281-021-00907-3 35022889

[69] Abacar K Macleod T Direskeneli H McGonagle D. How underappreciated autoinflammatory (innate immunity) mechanisms dominate disparate autoimmune disorders. Front Immunol. (2024) 15:1439371. doi: 10.3389/fimmu.2024.1439371 39372419 PMC11449752

[70] Williams CM Poore H Tanksley PT Kweon H Courchesne-Krak NS Londono-Correa D. Guidelines for evaluating the comparability of down-sampled GWAS summary statistics. Behav Genet. (2023) 53(5-6):404–15. doi: 10.1007/s10519-023-10152-z PMC10584908 37713023

[71] Kuiper JJ Prinz JC Stratikos E Kuśnierczyk P Arakawa A Springer S. EULAR study group on ‘MHC-I-opathy’: identifying disease-overarching mechanisms across disciplines and borders. Ann Rheum Dis. (2023) 82:887–96. doi: 10.1136/ard-2022-222852 PMC10313995 36987655

[72] Matzaraki V Kumar V Wijmenga C Zhernakova A. The MHC locus and genetic susceptibility to autoimmune and infectious diseases. Genome Biol. (2017) 18:76. doi: 10.1186/s13059-017-1207-1 28449694 PMC5406920

[73] Nunoi H Iwata M Tatsuzawa S Onoe Y Shimizu S Kanegasaki S. AG dinucleotide insertion in a patient with chronic granulomatous disease lacking cytosolic 67-kD protein. Blood. (1995) 86:329–33. doi: 10.1182/blood.V86.1.329.bloodjournal861329 7795241

[74] Tangye SG Al-Herz W Bousfiha A Cunningham-Rundles C Franco JL Holland SM. Human inborn errors of immunity: 2022 update on the classification from the international union of immunological societies expert committee. J Clin Immunol. (2022) 42:1473–507. doi: 10.1007/s10875-022-01289-3 PMC9244088 35748970

[75] Yamashita M Kuehn HS Okuyama K Okada S Inoue Y Mitsuiki N. A variant in human AIOLOS impairs adaptive immunity by interfering with IKAROS. Nat Immunol. (2021) 22:893–903. doi: 10.1038/s41590-021-00951-z 34155405 PMC8958960

[76] Ueki Y Takimoto-Ito R Saito MK Tanizaki H Kambe N. Tofacitinib, a suppressor of NOD2 expression, is a potential treatment for Blau syndrome. Front Immunol. (2023) 14:1211240. doi: 10.3389/fimmu.2023.1211240 37415984 PMC10321295

[77] Kayali S Fantasia S Gaiani F Cavallaro LG de’Angelis GL Laghi L. *NOD2* and crohn’s disease clinical practice: from epidemiology to diagnosis and therapy, rewired. Inflamm Bowel Dis. (2025) 31:552–62. doi: 10.1093/ibd/izae075 PMC11808579 38582044

[78] Aggeletopoulou I Assimakopoulos SF Konstantakis C Triantos C. Interleukin 12/interleukin 23 pathway: Biological basis and therapeutic effect in patients with Crohn’s disease. World J Gastroenterol. (2018) 24:4093–103. doi: 10.3748/wjg.v24.i36.4093 PMC6158482 30271076

[79] Koutruba N Emer J Lebwohl M. Review of ustekinumab, an interleukin-12 and interleukin-23 inhibitor used for the treatment of plaque psoriasis. Ther Clin Risk Manage. (2010) 6:123–41. doi: 10.2147/tcrm.s5599 PMC2857612 20421912

[80] van Munster KN Bergquist A Ponsioen CY. Inflammatory bowel disease and primary sclerosing cholangitis: One disease or two? J Hepatol. (2024) 80:155–68. doi: 10.1016/j.jhep.2023.09.031 37940453

[81] Larosa M Zen M Gatto M Jesus D Zanatta E Iaccarino L. IL-12 and IL-23/Th17 axis in systemic lupus erythematosus. Exp Biol Med (Maywood). (2019) 244:42–51. doi: 10.1177/1535370218824547 30664357 PMC6362534

[82] Shi W Xu Y Zhang A Jia X Liu S Hu Z. Inflammatory cytokines and their potential role in Sjogren’s syndrome risk: insights from a Mendelian randomization study. Adv Rheumatol. (2024) 64:14. doi: 10.1186/s42358-024-00354-2 38365917 PMC13488640

[83] Paroli M Spadea L Caccavale R Spadea L Paroli MP Nante N. The role of interleukin-17 in juvenile idiopathic arthritis: from pathogenesis to treatment. Medicina (Kaunas). (2022) 58:1552. doi: 10.3390/medicina58111552 36363508 PMC9696590

[84] Hammitzsch A Lorenz G Moog P. Impact of janus kinase inhibition on the treatment of axial spondyloarthropathies. Front Immunol. (2020) 11:591176. doi: 10.3389/fimmu.2020.591176 33193430 PMC7609840

[85] Mukai T Ida H Ueki Y Nishikomori R. Editorial: A new frontier in translational research on autoinflammatory diseases - various aspects of innate immunity on human diseases. Front Immunol. (2023) 14:1147202. doi: 10.3389/fimmu.2023.1147202 36798122 PMC9927384

[86] Uffelmann E Huang QQ Munung NS De Vries J Okada Y Martin AR. Genome-wide association studies. Nat Rev Methods Primers. (2021) 1:59. doi: 10.1038/s43586-021-00056-9

