# Supplementary material for: Mapping the genetic landscape of immune-mediated disorders: potential implications for classification and therapeutic strategies
Source: Front Immunol. 2025 May 8;16:1543781. doi: 10.3389/fimmu.2025.1543781 (PMC12094916; doi:10.3389/fimmu.2025.1543781)

Supplementary table 1. GWAS availability in immune disorders.

N	GWAS available*	N cases	N controls	N effective	Inclusion	Cause of exclusion	N SNPs	Source of GWAS (GWAS catalog/ PMID)
1	Addison’s disease	1223	4097	3767	0	N eff < 5000	Not included	33574239
2	Ankylosing spondylitis	9069	13578	21749	0	Only Immunochip	Not included	23749187
3	Autoimmune hepatitis	< 1000	< 1000	< 1000	0	N eff < 5000	Not included	Publication and GWAS catalog search
4	Autoimmune thyroiditis	30234	725172	114296	1	No	33663609	32581359
5	Bechet disease	< 1000	< 1000	< 1000	0	N eff < 5000	Not included	Publication and GWAS catalog search
6	Celiac disease	6897	334824	22142	1	No	9391264	20190752. 34278373
7	Crohn’s disease	12194	34915	36151	1	No	7829632	28067908
8	Dermatomyositis	< 1000	< 1000	< 1000	0	N eff < 5000	Not included	Publication search
9	Giant cell arteritis	1651	15306	5961	0	Not publicly avail.	Not included	25817017
10	Juvenile idiopathic arthritis	4799	294231	15670	1	No	18981388	R9 FinnGen. 33106285
11	Immune-mediated encephalitis	< 1000	< 1000	< 1000	0	N eff < 5000	Not included	Publication and GWAS catalog search
12	Inflammatory polyneuropathy	1261	823700	5036	0	Not publicly avail.	Not included	38851890
13	Multiple sclerosis	14802	26703	38093	1	No	8813102	31604244
14	Myasthenia gravis	1873	36370	7125	1	No	21992780	35074870
15	Narcolepsy	1886	10421	6388	0	Only Immunochip	Not included	23459209
16	Primary biliary cholangitis	8021	16489	21584	1	No	5054573	34033851
17	Primary sclerosing cholangitis	2 871	12019	9270	1	No	7616373	27992413
18	Primary Sjogren's syndrome	3232	17481	10911	1	No	6257360	35896530
19	Psoriasis (incl. Psoriatic Arthritis)	17255	693100	64320	1	No	20035071	R9 FinnGen. 34278373. 22482804
20	Rheumatoid arthritis	22350	74823	68838	1	No	13297691	36333501
21	Sarcoidosis	< 1000	< 1000	< 1000	0	N eff < 5000	Not included	Publication and GWAS catalog search
22	Systemic lupus erythematosus	5595	361571	15096	1	No	21079953	R9 FinnGen. 26502338. 29848360
23	Systemic sclerosis	9095	17584	23978	1	No	4419647	31672989
24	Type 1 diabetes	18 942	501 638	73011	1	No	62115238	34012112
25	Ulcerative colitis	12366	34915	36527	1	No	7844109	28067908
26	Vasculitis	< 1000	< 1000	< 1000	0	N eff < 5000	Not included	Publication and GWAS catalog search

**The cut-off for GWAS inclusion was August 2023.*

Supplementary table 2. In-house meta-analysis for SLE, CeD, PS, JIA.

Trait	PMID	N case	N control	N eff	Meta-analysis characteristics
**SLE** Metanalysis: Sample Size weighted	26502338	4036	6959	10217.93	Lambda GC = 1.165, Intercept =1.09 (SE=0.01)
	29848360	907	1524	2274.4	
	FinnGen R9 (L12_LUPUS)	652	353088	2603.2	
	**Sum. N eff**			15096	N SNPs = 21079953
**CeD** Metanalysis: Standard error weighted	20190752	4533	10750	12753.975	Lambda GC = 1.04, Intercept =1.02 (SE=0.01)
	34278373	2364	324074	9387.521502	
	**Sum. N eff**			22142	N SNPs = 9391264
**PS (with PsA)** Metanalysis: Sample Size weighted	34278373	5459	324074	21474.26772	Lambda GC =1.191, Intercept =1.08 (SE=0.01)
	22482804	2529	4955	6697.592197	
	FinnGen R9 (L12_PSORIASIS)	9267	364071	36147.89769	
	**Sum. N eff**			64320	N SNPs = 20035071
**JIA** Metanalysis: Sample Size weighted	FinnGen R9	1494	285035	5944.840348	Lambda GC =1.071, Intercept =1.02 (SE=0.01)
	Lopez_Isac	3305	9196	9724.911607	
	**Sum. N eff**			15670	N SNPs = 18981388

Supplementary Table 3. Population Prevalence.

Trait	Population prevalence	Source
Autoimmune thyroiditis	0.05	https://www.nature.com/articles/s41586-020-2436-0
Celiac disease	0.01	https://www.ncbi.nlm.nih.gov/pmc/articles/PMC2847618/
Crohn’s disease	0.00074	https://www.sciencedirect.com/science/article/abs/pii/S0025619617303130
Juvenile idiopathic arthritis	0.0003	https://www.sciencedirect.com/science/article/pii/S1297319X1300211X?via%3Dihub
Multiple sclerosis	0.002	https://bmcneurol.biomedcentral.com/articles/10.1186/1471-2377-13-128
Myasthenia gravis	0.0002	https://bmcneurol.biomedcentral.com/articles/10.1186/1471-2377-13-128
Primary biliary cholangitis	0.00015	https://www.ncbi.nlm.nih.gov/pmc/articles/PMC8812681/
Primary sclerosing cholangitis	0.00014	https://www.ncbi.nlm.nih.gov/pmc/articles/PMC10241503/
Myasthenia gravis	0.0002	https://www.ncbi.nlm.nih.gov/pmc/articles/PMC8812681/
Psoriasis	0.019	https://www.ncbi.nlm.nih.gov/pmc/articles/PMC7254147
Primary sclerosing cholangitis	0.00014	https://academic.oup.com/ibdjournal/advance-article-abstract/doi/10.1093/ibd/izad276/7459309
Primary biliary cholangitis	0.00015	https://www.ncbi.nlm.nih.gov/pmc/articles/PMC10241503/
Rheumatoid arthritis	0.004	https://www.nature.com/articles/s41584-022-00827-y
Sjogren's syndrome	0.00022	https://www.ncbi.nlm.nih.gov/pmc/articles/PMC4122257/
Systemic lupus erythematous	0.0003	https://academic.oup.com/rheumatology/article/56/11/1945/4079913
Systemic sclerosis	0.0002	https://www.ncbi.nlm.nih.gov/pmc/articles/PMC6497473/
Type 1 diabetes	0.003	https://www.ncbi.nlm.nih.gov/pmc/articles/PMC8563635/
Ulcerative colitis	0.0011	https://dmr.amegroups.org/article/view/6855/html

Supplementary Table 4. Genomic SEM EFA results.

Disease	Factor1
AITD	0.27059188
CD	0.15998654
CeD	0.13861951
JIA	0.6470404
MG	0.17584866
MS	0.3141415
PBC	0.58096884
PS	0.21073397
PSC	0.28334137
RA	0.55019317
SjS	0.96197984
SLE	0.91905468
SS	0.8525909
T1D	0.36263911
UC	0.21146054

Disease	Factor1	Factor2
AITD	0.28783435	-0.08258583
CD	-0.13457556	0.6697888
CeD	0.02564132	0.14190586
JIA	0.50417002	0.34503329
MG	0.07016606	0.18312524
MS	0.19387758	0.25947907
PBC	0.5516105	0.03524752
PS	0.13182161	0.19433816
PSC	0.00752186	0.63007694
RA	0.49601735	0.03317615
SjS	1.08512272	-0.2151765
SLE	0.96200473	-0.10576247
SS	0.8797846	-0.06418088
T1D	0.3102926	0.09581708
UC	-0.27718535	1.11399734

Disease	Factor1	Factor2	Factor3
AITD	0.08791468	0.36433673	-0.16169707
CD	-0.15077722	0.26750607	0.54685057
CeD	-0.14945825	0.3864664	0.02555064
JIA	0.13272402	0.88691645	0.10376513
MG	-0.10163244	0.40458699	0.06001185
MS	0.23444099	-0.01325956	0.27054927
PBC	0.3886049	0.3176435	-0.01137599
PS	-0.04429866	0.43196437	0.06653355
PSC	0.22796174	-0.25355632	0.68029839
RA	0.36167842	0.24247886	0.00356261
SjS	0.98333912	0.10029471	-0.1451469
SLE	0.9232548	-0.00749695	-0.01572183
SS	0.83555894	0.01897351	0.00610109
T1D	0.16813926	0.30886752	0.02591544
UC	-0.18657255	0.2028607	0.9755606

Disease	Factor1	Factor2	Factor3	Factor4
AITD	-0.03638129	-0.21128647	0.43693882	0.28948946
CD	-0.13775789	0.57362291	0.24724551	-0.04161846
CeD	-0.18121579	-0.00535936	0.02877843	0.74653794
JIA	0.2362397	0.20785905	0.60671264	0.05328938
MG	-0.12816237	0.0630787	0.28871635	0.27112278
MS	0.18633146	0.24393014	0.13275026	-0.09154913
PBC	0.36021417	-0.01224356	0.36732866	-0.01095033
PS	-0.16510636	0.04487205	0.64886726	-0.00781558
PSC	0.19374381	0.62265107	-0.21750285	0.10637735
RA	0.34212917	-0.02368462	0.06962574	0.39408256
SjS	1.01219111	-0.14293118	0.17880955	-0.19677795
SLE	1.02467378	-0.01776349	-0.27765604	0.2134382
SS	0.80754423	-0.01714603	0.18757434	-0.16971437
T1D	0.14260505	0.01562715	0.22686255	0.23105867
UC	-0.15440216	1.00690148	0.13214595	-0.02716267

Disease	Factor1	Factor2	Factor3	Factor4	Factor5
AITD	-0.07490425	0.61768881	-0.21714291	0.06062913	0.06034958
CD	0.01567545	0.00777259	0.6032393	-0.01350538	0.14517501
CeD	-0.22571146	0.58069284	-0.00591571	0.01884667	-0.00526142
JIA	0.12350392	0.67260231	0.24952198	-0.21170808	0.60927432
MG	-0.09859527	0.42055854	0.1056326	-0.09512572	0.11987014
MS	0.16024617	0.1403877	0.13927246	0.27910088	-0.08754383
PBC	0.24020756	0.65162455	-0.09977716	0.14345887	-0.02614483
PS	-0.09044864	0.48687341	0.06751587	0.00423384	0.1428573
PSC	-0.08232946	-0.01488055	0.23841646	0.96111237	-0.08572021
RA	0.3826432	0.33305413	0.05702208	-0.09519434	0.02362408
SjS	0.85086624	0.06359461	-0.13761829	0.0594167	0.3726598
SLE	0.7662784	0.01972289	-0.08051918	0.20183935	0.26153572
SS	1.00371898	0.04421795	0.1491732	-0.23576121	-0.07315518
T1D	0.01231399	0.3265617	-0.0316494	0.13698745	0.28492639
UC	-0.01571186	-0.02723995	0.91591964	0.27470926	0.03016954

Supplementary Table 5. Genomic SEM models performance.

Number of factors	chisq	df	p_chisq	AIC	CFI	SRMR
**1**	1321.83	90	4.561E-218	1381.83	0.658	0.124
**2**	1073.7	88	3.076E-169	1137.7	0.727	0.111
**3**	779.35	87	9.092E-112	845.35	0.808	0.094
**4**	215.59	56	1.420E-20	285.59	0.919	0.077
**5**	238.37	54	5.254E-25	312.37	0.907	0.083

4-factor model:

F1 =~ NA*PBC + SjS + SLE + SS + RA

F2 =~ NA*CeD + RA + MG

F3 =~ NA*AITD + JIA + PBC + PS + MG

F4 =~ NA*CD + PSC + UC

F1~~F2
F1~~F3
F2~~F3
F1~~F4
F2~~F4
F3~~F4
F1~~1*F1
F2~~1*F2
F3~~1*F3
F4~~1*F4
JIA~~a*JIA
a>0.001

Supplementary Table 6. Genomic SEM CFA performance for 4-factors model.

	Op	rhs	Unstand_Est	Unstand_SE	STD_Genotype	STD_Genotype_SE	STD_All	p_value
F1	=~	PBC	0.1226357	0.0375697	0.3408133	0.1040994	0.3408133	0.0010977
F1	=~	SjS	0.2552986	0.0249955	0.9459967	0.0926193	0.9459969	0.0000000
F1	=~	SLE	0.3578760	0.0270762	0.8988951	0.0679994	0.8988951	0.0000000
F1	=~	SS	0.1721013	0.0173406	0.9208427	0.0927831	0.9208427	0.0000000
F1	=~	RA	0.1291846	0.0215772	0.4633557	0.0774199	0.4633556	0.0000000
F4	=~	CD	0.2427367	0.0226510	0.6528448	0.0609161	0.6528448	0.0000000
F4	=~	PSC	0.1833089	0.0285418	0.5886449	0.0916620	0.5886451	0.0000000
F4	=~	UC	0.2810718	0.0226160	0.8613701	0.0692999	0.8613699	0.0000000
F3	=~	AITD	0.1461157	0.0197334	0.3878799	0.0523697	0.3878799	0.0000000
F3	=~	JIA	0.2874839	0.0228175	1.0026142	0.0793863	1.0026142	0.0000000
F3	=~	PBC	0.1296491	0.0475326	0.3595053	0.1317368	0.3595053	0.0063800
F3	=~	PS	0.1458465	0.0152912	0.4485205	0.0469995	0.4485205	0.0000000
F3	=~	MG	0.0684034	0.0862176	0.2046345	0.2574026	0.2046348	0.4275561
F2	=~	CeD	0.1843755	0.0480744	0.6266083	0.1634714	0.6266067	0.0001255
F2	=~	RA	0.1016653	0.0218467	0.3649611	0.0784513	0.3649611	0.0000033
F2	=~	MG	0.1172441	0.1108009	0.3498509	0.3309443	0.3498514	0.2899867
F1	~~	F4	0.2539975	0.0481916	0.2540040	0.0481855	0.2540040	0.0000001
F1	~~	F3	0.5117707	0.0580181	0.5108531	0.0578724	0.5108531	0.0000000
F4	~~	F3	0.4952397	0.0651556	0.4945141	0.0650505	0.4945141	0.0000000
F1	~~	F2	0.2385413	0.1499757	0.2386248	0.1499899	0.2386248	0.1117146
F4	~~	F2	0.2467450	0.0993132	0.2467058	0.0992512	0.2467058	0.0129729
F3	~~	F2	0.7349839	0.1582248	0.7331663	0.1580213	0.7331663	0.0000034
JIA	~~	JIA	0.0010004	0.0126979	0.0009999	0.1539318	0.0009999	0.9372053
AITD	~~	AITD	0.1206018	0.0111606	0.8495492	0.0786287	0.8495492	0.0000000
CD	~~	CD	0.0793119	0.0112790	0.5737937	0.0815844	0.5737937	0.0000000
CeD	~~	CeD	0.0526028	0.0244244	0.6073673	0.2820475	0.6073641	0.0312639
MG	~~	MG	0.0818759	0.0240061	0.7307492	0.2142094	0.7307513	0.0006481
PBC	~~	PBC	0.0817271	0.0104666	0.6294184	0.0805891	0.6294185	0.0000000
PS	~~	PS	0.0845464	0.0096939	0.7988294	0.0916036	0.7988294	0.0000000
PSC	~~	PSC	0.0633476	0.0168757	0.6534967	0.1740638	0.6534970	0.0001742
RA	~~	RA	0.0444138	0.0066244	0.5713991	0.0853464	0.5713989	0.0000000
SjS	~~	SjS	0.0076524	0.0177657	0.1050898	0.2439210	0.1050898	0.6666558
SLE	~~	SLE	0.0304375	0.0214499	0.1919875	0.1352996	0.1919875	0.1558990
SS	~~	SS	0.0053113	0.0055132	0.1520487	0.1578418	0.1520487	0.3353578
UC	~~	UC	0.0275058	0.0102837	0.2580420	0.0965684	0.2580419	0.0074795
F1	~~	F1	1.0000000		1.0000000		1.0000000	
F2	~~	F2	1.0000000		1.0000000		1.0000000	
F3	~~	F3	1.0000000		1.0000000		1.0000000	
F4	~~	F4	1.0000000		1.0000000		1.0000000	

Supplementary Table 7. Univariate MiXeR results.

**Disease**	**pi (mean)**	**pi (std)**	**sig2_beta (mean)**	**sig2_beta (std)**	**sig2_zero (mean)**	**sig2_zero (std)**
**AITD**	0.0002258754572637227	6.296571757322334e-06	0.0004142065235941271	1.248894195115614e-05	1.051499917296796	0.0018908080073300952
**MG**	0.00019486019562114525	8.835604578509106e-05	0.0007519291939711977	0.00018718837242542657	1.033487088977847	0.0027045010525844045
**SjS**	1.0217085217058164e-05	3.6671559570522776e-06	0.010829486806931176	0.002710162720844314	1.1119317236708093	0.0028048715634420695
**PBC**	9.69675311420012e-05	1.1452685128750887e-05	0.0024089928994669345	0.00020471083678606151	1.0052756035837265	0.0052325665914501876
**RA**	0.0002149697190220001	9.929569814840124e-06	0.0003805620574363664	1.3535296113223775e-05	1.0555950270024552	0.000355607144355682
**PSC**	1.764254024562759e-05	6.583724322182204e-06	0.006190536969480835	0.002877398970171814	1.0413287023816493	0.0007629720826936351
**T1D**	8.699948598471239e-05	1.4482061848903853e-05	0.0011210966010805604	0.00019419188616911403	1.0571588595444388	0.001179859547551263
**SS**	6.379028817305981e-05	1.9988291771423187e-05	0.0007931071118662508	0.0001905892473110814	1.0512327369305416	0.00421332282942373
**CeD**	2.878324373393513e-06	6.323769501320039e-07	0.048997556878031395	0.01537697029380353	1.0169906130288815	0.00022665013281779586
**JIA**	0.00011134699857697004	2.3930706720584143e-05	0.0008840044004000036	0.00012978083892303496	1.0125021548596145	0.00045583102487824917
**PS**	0.00012676671765309418	1.5705466441467305e-05	0.0006881934474355373	8.131076384519171e-05	1.0618428501374646	0.0006434700164874836
**MS**	0.00017864020375033893	8.392099171208585e-06	0.0008868828762638792	4.261630832345097e-05	1.0419837958063511	0.0005293571942966783
**SLE**	0.00017159185758468387	1.2028345713991701e-05	0.001723797290146849	9.053890878450303e-05	1.0168236667387052	0.00040171088751266416
**CD**	0.00010892220475896405	1.2892204256803917e-05	0.002019038776116613	0.00022384934817038562	1.1469453171186277	0.0008946811086211929
**UC**	0.0001444976551940322	8.196037696691804e-06	0.0009636894966156063	3.478434835722532e-05	1.1240906587388355	0.0004965040616468337
	**h2 (mean)**	**h2 (std)**	**nc@p9 (mean)**	**nc@p9 (std)**	**AIC**	**BIC**
**AITD**	0.1939353604200073	0.0021764266547036524	720.3431903952444	20.08050207476273	2596.732604992873	2587.109851229674
**MG**	0.2720042247118634	0.028194672757721227	621.431901877199	281.77774018728206	6.783257736187079	-2.79194215394964
**SjS**	0.21096820030385607	0.01590400894794699	32.58347697865538	11.694988263801186	269.3023085411434	259.9882034119015
**PBC**	0.4799011794476897	0.01902387081826567	309.24077185608337	36.52394927796157	1325.9422597549885	1317.1502458237142
**RA**	0.1694559061503081	0.003242820567555825	685.5635185627011	31.666556811097273	876.31211893562	887.2249960184447
**PSC**	0.19502196621683673	0.018753316780380506	56.264119533685104	20.996265111646476	150.04651349306806	160.3752507519792
**T1D**	0.19661823714568713	0.004275773255816583	277.4515126882679	46.184985133469475	2394.5393661450244	2405.4404176663256
**SS**	0.09736267913269993	0.011389807268509553	203.43467260882434	63.74499487284707	94.12511542791762	85.09764574522414
**CeD**	0.27457290257275524	0.03536368741681363	9.179312295544722	2.0167238854048946	630.8544523319346	641.6217596134287
**JIA**	0.1978498733735608	0.014427234133018238	355.09857143187446	76.31781618217936	77.83019802758936	88.71841404626612
**PS**	0.1783973753596328	0.004194930425076043	404.27385487723353	50.086565137012826	1016.4200968658319	1027.3633665954462
**MS**	0.32800541670149586	0.007674647524250213	569.7044551065662	26.76338352264436	1046.214430807327	1056.75314044783
**SLE**	0.6115201986695855	0.01730890300906205	547.2264567198265	38.35979805755256	576.617299238185	587.501049200038
**CD**	0.4503228034705343	0.008464449464897356	347.36562099948753	41.11474375337021	2529.2796581848174	2540.117963658145
**UC**	0.28836545802954294	0.008917969428940744	460.81988370065545	26.138120602192966	1053.5622501847452	1064.4021559235757

Supplementary Table 8. Bivariate MiXeR results.

trait1	trait2	dice (mean)	dice (std)	pi1 (mean)	pi1 (std)	pi2 (mean)	pi2 (std)	pi12 (mean)	pi12 (std)	nc1@p9 (mean)	nc1@p9 (std)	nc2@p9 (mean)	nc2@p9 (std)	nc12@p9 (mean)	nc12@p9 (std)
**AITD**	**JIA**	6.13E-01	8.47E-02	1.22E-04	2.02E-05	7.02E-06	8.72E-06	1.04E-04	2.18E-05	3.88E+02	6.45E+01	2.24E+01	2.78E+01	3.33E+02	6.96E+01
**AITD**	**MS**	7.16E-01	5.22E-02	8.10E-05	1.26E-05	3.38E-05	1.03E-05	1.45E-04	1.25E-05	2.58E+02	4.02E+01	1.08E+02	3.29E+01	4.62E+02	3.99E+01
**AITD**	**PBC**	5.77E-01	5.01E-02	1.33E-04	1.25E-05	3.60E-06	3.58E-06	9.34E-05	1.13E-05	4.23E+02	3.98E+01	1.15E+01	1.14E+01	2.98E+02	3.60E+01
**AITD**	**PS**	5.70E-01	5.22E-02	1.25E-04	1.33E-05	2.60E-05	9.80E-06	1.01E-04	1.24E-05	3.99E+02	4.26E+01	8.29E+01	3.13E+01	3.21E+02	3.97E+01
**AITD**	**RA**	8.23E-01	7.11E-02	4.43E-05	1.69E-05	3.34E-05	1.58E-05	1.82E-04	1.81E-05	1.41E+02	5.37E+01	1.07E+02	5.05E+01	5.79E+02	5.78E+01
**AITD**	**SLE**	6.30E-01	5.51E-02	1.01E-04	1.14E-05	4.63E-05	1.25E-05	1.25E-04	1.24E-05	3.21E+02	3.63E+01	1.48E+02	3.99E+01	4.00E+02	3.96E+01
**AITD**	**SS**	3.95E-01	1.10E-01	1.68E-04	2.10E-05	5.66E-06	7.55E-06	5.81E-05	1.95E-05	5.35E+02	6.69E+01	1.81E+01	2.41E+01	1.85E+02	6.21E+01
**AITD**	**TD1**	5.08E-01	4.43E-02	1.46E-04	1.09E-05	7.33E-06	7.45E-06	7.97E-05	1.00E-05	4.66E+02	3.47E+01	2.34E+01	2.38E+01	2.54E+02	3.20E+01
**CD**	**AITD**	5.03E-01	5.38E-02	2.47E-05	1.36E-05	1.42E-04	9.85E-06	8.42E-05	9.66E-06	7.89E+01	4.35E+01	4.52E+02	3.14E+01	2.68E+02	3.08E+01
**CD**	**JIA**	8.20E-01	9.66E-02	1.84E-05	1.46E-05	2.08E-05	1.84E-05	9.06E-05	1.73E-05	5.86E+01	4.66E+01	6.63E+01	5.88E+01	2.89E+02	5.52E+01
**CD**	**MG**	5.23E-01	1.27E-01	3.04E-05	2.30E-05	1.16E-04	7.40E-05	7.85E-05	2.63E-05	9.70E+01	7.34E+01	3.71E+02	2.36E+02	2.50E+02	8.40E+01
**CD**	**MS**	6.78E-01	6.25E-02	1.13E-05	9.47E-06	8.10E-05	1.29E-05	9.76E-05	1.17E-05	3.60E+01	3.02E+01	2.58E+02	4.12E+01	3.11E+02	3.73E+01
**CD**	**PBC**	6.35E-01	6.87E-02	4.34E-05	1.07E-05	3.14E-05	1.07E-05	6.56E-05	1.06E-05	1.38E+02	3.40E+01	1.00E+02	3.40E+01	2.09E+02	3.37E+01
**CD**	**PS**	6.97E-01	5.00E-02	2.68E-05	9.97E-06	4.47E-05	1.21E-05	8.21E-05	9.72E-06	8.56E+01	3.18E+01	1.43E+02	3.87E+01	2.62E+02	3.10E+01
**CD**	**RA**	6.28E-01	4.70E-02	7.05E-06	6.50E-06	1.13E-04	1.26E-05	1.02E-04	1.09E-05	2.25E+01	2.07E+01	3.61E+02	4.02E+01	3.25E+02	3.48E+01
**CD**	**SLE**	4.26E-01	4.66E-02	4.92E-05	1.06E-05	1.12E-04	1.44E-05	5.97E-05	7.26E-06	1.57E+02	3.39E+01	3.57E+02	4.61E+01	1.90E+02	2.31E+01
**CD**	**SS**	2.54E-01	7.26E-02	8.71E-05	1.40E-05	4.20E-05	1.93E-05	2.18E-05	6.45E-06	2.78E+02	4.47E+01	1.34E+02	6.16E+01	6.96E+01	2.06E+01
**CD**	**TD1**	6.02E-01	6.93E-02	5.00E-05	1.41E-05	2.81E-05	1.12E-05	5.89E-05	8.44E-06	1.60E+02	4.50E+01	8.96E+01	3.58E+01	1.88E+02	2.69E+01
**JIA**	**MS**	6.79E-01	7.76E-02	1.22E-05	1.08E-05	7.95E-05	1.93E-05	9.92E-05	1.79E-05	3.89E+01	3.44E+01	2.53E+02	6.16E+01	3.16E+02	5.71E+01
**JIA**	**SS**	6.56E-01	1.20E-01	5.38E-05	2.69E-05	6.25E-06	8.95E-06	5.75E-05	1.48E-05	1.72E+02	8.57E+01	1.99E+01	2.85E+01	1.83E+02	4.73E+01
**MG**	**AITD**	6.65E-01	1.27E-01	5.71E-05	7.93E-05	8.81E-05	3.00E-05	1.38E-04	2.97E-05	1.82E+02	2.53E+02	2.81E+02	9.56E+01	4.39E+02	9.48E+01
**MG**	**JIA**	5.72E-01	1.43E-01	1.08E-04	6.92E-05	2.45E-05	2.03E-05	8.69E-05	3.21E-05	3.44E+02	2.21E+02	7.80E+01	6.47E+01	2.77E+02	1.02E+02
**MG**	**MS**	5.27E-01	8.06E-02	9.62E-05	6.68E-05	8.00E-05	2.94E-05	9.87E-05	2.76E-05	3.07E+02	2.13E+02	2.55E+02	9.39E+01	3.15E+02	8.79E+01
**MG**	**PBC**	4.15E-01	1.45E-01	1.35E-04	8.26E-05	3.76E-05	2.54E-05	5.94E-05	2.23E-05	4.32E+02	2.63E+02	1.20E+02	8.10E+01	1.89E+02	7.11E+01
**MG**	**PS**	2.83E-01	8.53E-02	1.48E-04	7.05E-05	8.04E-05	3.22E-05	4.64E-05	2.29E-05	4.74E+02	2.25E+02	2.56E+02	1.03E+02	1.48E+02	7.29E+01
**MG**	**RA**	7.85E-01	1.09E-01	3.40E-05	6.35E-05	5.41E-05	3.78E-05	1.61E-04	3.91E-05	1.08E+02	2.03E+02	1.73E+02	1.20E+02	5.13E+02	1.25E+02
**MG**	**SLE**	5.82E-01	1.26E-01	8.78E-05	6.50E-05	6.46E-05	3.46E-05	1.07E-04	3.51E-05	2.80E+02	2.07E+02	2.06E+02	1.10E+02	3.41E+02	1.12E+02
**MG**	**SS**	3.50E-01	1.45E-01	1.51E-04	9.02E-05	2.01E-05	1.24E-05	4.37E-05	1.97E-05	4.82E+02	2.88E+02	6.40E+01	3.97E+01	1.39E+02	6.29E+01
**MG**	**TD1**	5.36E-01	1.30E-01	1.23E-04	8.18E-05	1.53E-05	1.62E-05	7.17E-05	1.43E-05	3.93E+02	2.61E+02	4.89E+01	5.17E+01	2.29E+02	4.57E+01
**MS**	**SS**	4.63E-01	1.04E-01	1.22E-04	1.94E-05	6.99E-06	7.35E-06	5.68E-05	1.61E-05	3.89E+02	6.20E+01	2.23E+01	2.34E+01	1.81E+02	5.15E+01
**PBC**	**JIA**	8.33E-01	7.40E-02	1.04E-05	1.16E-05	2.48E-05	2.17E-05	8.66E-05	1.06E-05	3.31E+01	3.70E+01	7.89E+01	6.94E+01	2.76E+02	3.37E+01
**PBC**	**MS**	6.94E-01	5.65E-02	1.17E-06	1.03E-06	8.28E-05	1.39E-05	9.58E-05	1.13E-05	3.73E+00	3.27E+00	2.64E+02	4.45E+01	3.06E+02	3.62E+01
**PBC**	**PS**	6.19E-01	5.47E-02	2.77E-05	9.08E-06	5.75E-05	1.30E-05	6.93E-05	9.55E-06	8.83E+01	2.90E+01	1.83E+02	4.16E+01	2.21E+02	3.04E+01
**PBC**	**RA**	6.09E-01	5.03E-02	1.73E-06	2.21E-06	1.20E-04	1.35E-05	9.52E-05	1.13E-05	5.50E+00	7.05E+00	3.82E+02	4.30E+01	3.04E+02	3.61E+01
**PBC**	**SLE**	6.71E-01	5.80E-02	6.92E-06	6.91E-06	8.15E-05	1.51E-05	9.00E-05	8.79E-06	2.21E+01	2.20E+01	2.60E+02	4.82E+01	2.87E+02	2.80E+01
**PBC**	**SS**	7.33E-01	1.25E-01	3.72E-05	1.97E-05	4.02E-06	5.11E-06	5.98E-05	1.61E-05	1.19E+02	6.28E+01	1.28E+01	1.63E+01	1.91E+02	5.13E+01
**PBC**	**TD1**	7.40E-01	7.32E-02	2.90E-05	1.18E-05	1.90E-05	1.30E-05	6.80E-05	8.89E-06	9.24E+01	3.75E+01	6.06E+01	4.16E+01	2.17E+02	2.84E+01
**PS**	**JIA**	7.41E-01	8.80E-02	3.82E-05	1.91E-05	2.28E-05	1.61E-05	8.86E-05	1.77E-05	1.22E+02	6.08E+01	7.26E+01	5.15E+01	2.83E+02	5.64E+01
**PS**	**MS**	6.76E-01	5.42E-02	2.34E-05	1.25E-05	7.53E-05	1.19E-05	1.03E-04	1.16E-05	7.47E+01	3.99E+01	2.40E+02	3.80E+01	3.30E+02	3.70E+01
**PS**	**RA**	6.24E-01	6.23E-02	2.01E-05	1.38E-05	1.08E-04	1.44E-05	1.07E-04	1.29E-05	6.41E+01	4.40E+01	3.45E+02	4.60E+01	3.40E+02	4.11E+01
**PS**	**SLE**	4.45E-01	6.33E-02	6.05E-05	1.62E-05	1.05E-04	1.34E-05	6.63E-05	9.89E-06	1.93E+02	5.17E+01	3.36E+02	4.28E+01	2.11E+02	3.15E+01
**PS**	**SS**	4.26E-01	6.92E-02	8.56E-05	1.23E-05	2.27E-05	1.51E-05	4.11E-05	1.10E-05	2.73E+02	3.91E+01	7.23E+01	4.81E+01	1.31E+02	3.51E+01
**RA**	**JIA**	6.59E-01	8.62E-02	1.06E-04	2.35E-05	2.85E-06	3.38E-06	1.08E-04	2.20E-05	3.40E+02	7.51E+01	9.10E+00	1.08E+01	3.46E+02	7.02E+01
**RA**	**MS**	7.71E-01	7.23E-02	6.33E-05	1.50E-05	2.70E-05	1.63E-05	1.52E-04	1.44E-05	2.02E+02	4.78E+01	8.60E+01	5.20E+01	4.84E+02	4.59E+01
**RA**	**SLE**	8.14E-01	3.86E-02	5.78E-05	1.27E-05	1.44E-05	8.25E-06	1.57E-04	8.58E-06	1.84E+02	4.06E+01	4.59E+01	2.63E+01	5.01E+02	2.74E+01
**RA**	**SS**	4.46E-01	1.06E-01	1.52E-04	1.96E-05	5.13E-07	9.61E-07	6.33E-05	1.94E-05	4.84E+02	6.26E+01	1.64E+00	3.06E+00	2.02E+02	6.18E+01

**SLE**	**JIA**	7.16E-01	7.83E-02	6.98E-05	2.10E-05	9.52E-06	1.09E-05	1.02E-04	1.78E-05	2.22E+02	6.68E+01	3.03E+01	3.49E+01	3.25E+02	5.68E+01
**SLE**	**MS**	6.96E-01	7.00E-02	4.97E-05	1.62E-05	5.68E-05	1.44E-05	1.22E-04	1.24E-05	1.59E+02	5.17E+01	1.81E+02	4.59E+01	3.89E+02	3.97E+01
**SLE**	**SS**	5.32E-01	1.28E-01	1.08E-04	2.32E-05	1.52E-08	3.80E-08	6.38E-05	2.00E-05	3.44E+02	7.41E+01	4.85E-02	1.21E-01	2.03E+02	6.37E+01
**TD1**	**JIA**	7.62E-01	1.12E-01	1.17E-05	1.18E-05	3.61E-05	2.16E-05	7.53E-05	1.50E-05	3.74E+01	3.75E+01	1.15E+02	6.88E+01	2.40E+02	4.77E+01
**TD1**	**MS**	5.70E-01	4.92E-02	1.12E-05	8.77E-06	1.03E-04	1.05E-05	7.58E-05	9.84E-06	3.56E+01	2.80E+01	3.28E+02	3.35E+01	2.42E+02	3.14E+01
**TD1**	**PS**	4.69E-01	6.17E-02	3.67E-05	9.18E-06	7.64E-05	1.59E-05	5.03E-05	9.53E-06	1.17E+02	2.93E+01	2.44E+02	5.09E+01	1.61E+02	3.04E+01
**TD1**	**RA**	5.44E-01	6.97E-02	4.46E-06	4.87E-06	1.32E-04	1.59E-05	8.25E-05	1.43E-05	1.42E+01	1.55E+01	4.22E+02	5.06E+01	2.63E+02	4.57E+01
**TD1**	**SLE**	5.69E-01	6.51E-02	1.33E-05	9.48E-06	9.79E-05	1.62E-05	7.37E-05	1.05E-05	4.24E+01	3.02E+01	3.12E+02	5.18E+01	2.35E+02	3.36E+01
**TD1**	**SS**	2.90E-01	9.30E-02	6.51E-05	1.61E-05	4.19E-05	1.61E-05	2.19E-05	8.04E-06	2.08E+02	5.12E+01	1.34E+02	5.15E+01	6.98E+01	2.56E+01
**UC**	**AITD**	4.76E-01	7.14E-02	5.64E-05	1.56E-05	1.38E-04	1.36E-05	8.81E-05	1.33E-05	1.80E+02	4.98E+01	4.39E+02	4.34E+01	2.81E+02	4.25E+01
**UC**	**CD**	7.80E-01	4.68E-02	4.53E-05	1.08E-05	9.73E-06	3.60E-06	9.92E-05	1.21E-05	1.44E+02	3.45E+01	3.10E+01	1.15E+01	3.16E+02	3.86E+01
**UC**	**JIA**	7.44E-01	6.87E-02	4.90E-05	1.65E-05	1.58E-05	1.32E-05	9.55E-05	1.54E-05	1.56E+02	5.25E+01	5.04E+01	4.20E+01	3.05E+02	4.91E+01
**UC**	**MG**	3.36E-01	1.42E-01	8.76E-05	2.80E-05	1.38E-04	8.16E-05	5.69E-05	2.92E-05	2.79E+02	8.92E+01	4.40E+02	2.60E+02	1.81E+02	9.32E+01
**UC**	**MS**	7.28E-01	4.40E-02	2.70E-05	9.19E-06	6.11E-05	1.05E-05	1.18E-04	7.68E-06	8.61E+01	2.93E+01	1.95E+02	3.34E+01	3.75E+02	2.45E+01
**UC**	**PBC**	6.88E-01	5.39E-02	6.15E-05	1.13E-05	1.40E-05	1.03E-05	8.30E-05	6.97E-06	1.96E+02	3.62E+01	4.46E+01	3.29E+01	2.65E+02	2.22E+01
**UC**	**PS**	6.49E-01	5.32E-02	5.65E-05	8.75E-06	3.87E-05	1.23E-05	8.80E-05	9.63E-06	1.80E+02	2.79E+01	1.24E+02	3.93E+01	2.81E+02	3.07E+01
**UC**	**RA**	6.41E-01	7.21E-02	2.92E-05	1.41E-05	9.96E-05	1.46E-05	1.15E-04	1.37E-05	9.30E+01	4.50E+01	3.18E+02	4.67E+01	3.68E+02	4.38E+01
**UC**	**SLE**	4.69E-01	5.03E-02	7.04E-05	8.93E-06	9.75E-05	1.37E-05	7.41E-05	8.40E-06	2.24E+02	2.85E+01	3.11E+02	4.38E+01	2.36E+02	2.68E+01
**UC**	**SS**	2.97E-01	9.17E-02	1.14E-04	1.47E-05	3.29E-05	1.83E-05	3.09E-05	1.03E-05	3.62E+02	4.69E+01	1.05E+02	5.82E+01	9.85E+01	3.29E+01
**UC**	**TD1**	5.55E-01	6.28E-02	8.02E-05	1.18E-05	2.27E-05	1.08E-05	6.43E-05	9.08E-06	2.56E+02	3.76E+01	7.25E+01	3.46E+01	2.05E+02	2.90E+01

trait1	trait2	rho_zero (mean)	rho_zero (std)	rho_beta (mean)	rho_beta (std)	rg (mean)	rg (std)	frac_conc. (mean)	frac_conc (std)	best_vs_min AIC	best_vs_min BIC	best_vs_max AIC	best_vs_max BIC
**AITD**	**JIA**	2.09E-02	2.78E-03	5.02E-01	6.28E-02	3.26E-01	2.45E-02	6.68E-01	2.35E-02	2.47E+01	1.51E+01	1.96E+00	-7.62E+00
**AITD**	**MS**	7.27E-03	2.16E-03	1.53E-01	2.33E-02	1.09E-01	1.12E-02	5.49E-01	7.51E-03	1.49E+02	1.39E+02	1.26E+01	3.05E+00
**AITD**	**PBC**	2.54E-02	3.19E-03	3.30E-01	3.84E-02	2.07E-01	1.69E-02	6.07E-01	1.29E-02	9.99E+01	9.12E+01	2.51E+00	-6.28E+00
**AITD**	**PS**	2.29E-02	2.42E-03	4.53E-01	3.50E-02	2.69E-01	1.33E-02	6.50E-01	1.24E-02	5.54E+01	4.58E+01	1.16E+01	2.04E+00
**AITD**	**RA**	3.25E-02	1.76E-03	3.56E-01	3.59E-02	2.91E-01	1.11E-02	6.16E-01	1.23E-02	8.13E+01	7.17E+01	1.11E+01	1.51E+00
**AITD**	**SLE**	1.75E-02	2.18E-03	2.37E-01	2.37E-02	1.50E-01	1.29E-02	5.76E-01	7.78E-03	7.03E+01	6.07E+01	1.17E+01	2.09E+00
**AITD**	**SS**	1.07E-02	3.29E-03	3.60E-01	1.12E-01	1.64E-01	3.58E-02	6.18E-01	3.88E-02	2.01E+01	1.11E+01	6.14E-01	-8.41E+00
**AITD**	**TD1**	5.83E-02	2.53E-03	5.96E-01	4.08E-02	3.38E-01	1.30E-02	7.04E-01	1.61E-02	1.03E+02	9.34E+01	5.35E+00	-4.22E+00
**CD**	**AITD**	3.37E-03	1.69E-03	2.12E-02	1.95E-02	1.14E-02	1.00E-02	5.07E-01	6.22E-03	1.95E+02	1.85E+02	1.84E+01	8.84E+00
**CD**	**JIA**	1.17E-01	2.11E-03	5.46E-01	9.42E-02	4.43E-01	2.50E-02	6.86E-01	3.85E-02	2.63E+01	1.67E+01	3.97E+00	-5.57E+00
**CD**	**MG**	4.14E-03	1.83E-03	1.12E-01	5.91E-02	6.06E-02	3.39E-02	5.36E-01	1.90E-02	1.65E+01	6.97E+00	7.37E+00	-2.16E+00
**CD**	**MS**	4.20E-02	2.21E-03	2.87E-01	2.96E-02	1.99E-01	1.23E-02	5.93E-01	9.89E-03	1.12E+02	1.02E+02	4.75E+00	-4.77E+00
**CD**	**PBC**	1.47E-01	4.22E-03	3.78E-01	5.69E-02	2.38E-01	2.69E-02	6.24E-01	1.95E-02	6.60E+01	5.72E+01	2.45E+01	1.57E+01
**CD**	**PS**	1.67E-02	2.42E-03	3.94E-01	3.20E-02	2.75E-01	1.69E-02	6.29E-01	1.11E-02	8.98E+01	8.02E+01	1.84E+01	8.82E+00
**CD**	**RA**	6.53E-03	2.71E-03	3.20E-01	3.45E-02	2.12E-01	2.05E-02	6.04E-01	1.16E-02	8.15E+01	7.20E+01	5.72E+00	-3.82E+00
**CD**	**SLE**	1.57E-03	1.70E-03	2.90E-01	4.33E-02	1.26E-01	1.62E-02	5.94E-01	1.44E-02	3.44E+01	2.49E+01	2.59E+01	1.64E+01
**CD**	**SS**	1.70E-02	3.13E-03	7.84E-01	2.07E-01	1.97E-01	2.78E-02	8.13E-01	1.11E-01	1.66E+00	-7.34E+00	2.07E+01	1.17E+01
**CD**	**TD1**	3.60E-02	2.28E-03	3.06E-01	3.84E-02	1.84E-01	1.51E-02	5.99E-01	1.29E-02	1.18E+02	1.08E+02	3.71E+01	2.76E+01
**JIA**	**MS**	7.47E-03	1.77E-03	3.87E-01	6.58E-02	2.69E-01	3.00E-02	6.27E-01	2.32E-02	1.74E+01	7.83E+00	4.57E-01	-9.09E+00
**JIA**	**SS**	1.07E-02	2.25E-03	7.59E-01	1.34E-01	5.15E-01	5.48E-02	7.91E-01	8.89E-02	3.64E+00	-5.37E+00	-4.89E-01	-9.51E+00
**MG**	**AITD**	1.07E-02	1.98E-03	6.24E-01	1.13E-01	4.11E-01	2.94E-02	7.18E-01	4.83E-02	8.95E+00	-6.26E-01	3.89E+00	-5.68E+00
**MG**	**JIA**	5.91E-03	1.74E-03	4.85E-01	1.60E-01	2.74E-01	5.78E-02	6.66E-01	6.49E-02	9.31E-01	-8.62E+00	-2.98E-01	-9.85E+00
**MG**	**MS**	-1.07E-03	1.74E-03	4.40E-01	9.37E-02	2.31E-01	3.60E-02	6.46E-01	3.39E-02	3.28E+00	-6.26E+00	3.89E+00	-5.64E+00
**MG**	**PBC**	2.61E-03	2.84E-03	3.93E-01	1.80E-01	1.53E-01	4.31E-02	6.34E-01	7.64E-02	3.46E+00	-5.31E+00	2.32E+00	-6.44E+00
**MG**	**PS**	-4.92E-04	2.13E-03	7.39E-01	1.82E-01	2.04E-01	3.23E-02	7.81E-01	9.25E-02	1.84E+00	-7.72E+00	9.57E+00	6.91E-03
**MG**	**RA**	1.42E-02	1.60E-03	5.62E-01	9.05E-02	4.41E-01	2.54E-02	6.92E-01	3.73E-02	1.01E+01	5.43E-01	2.04E+00	-7.53E+00

**MG**	**SLE**	9.70E-03	1.50E-03	2.50E-01	8.36E-02	1.39E-01	2.90E-02	5.81E-01	2.77E-02	5.81E+00	-3.74E+00	1.59E+00	-7.95E+00
**MG**	**SS**	5.48E-03	2.82E-03	-1.05E-01	2.77E-01	-5.12E-02	8.46E-02	4.67E-01	9.45E-02	1.44E+00	-7.56E+00	-9.62E-02	-9.10E+00
**MG**	**TD1**	9.57E-03	2.32E-03	5.09E-01	7.61E-02	2.87E-01	3.95E-02	6.71E-01	2.78E-02	1.01E+01	5.53E-01	2.98E+00	-6.57E+00
**MS**	**SS**	8.79E-02	3.07E-03	4.25E-01	8.23E-02	2.22E-01	4.13E-02	6.40E-01	2.94E-02	1.31E+01	4.11E+00	-3.03E-01	-9.32E+00
**PBC**	**JIA**	1.58E-01	3.12E-03	6.10E-01	6.12E-02	5.11E-01	3.58E-02	7.10E-01	2.42E-02	1.78E+01	9.00E+00	4.02E-01	-8.37E+00
**PBC**	**MS**	7.74E-02	3.58E-03	6.26E-01	6.16E-02	4.53E-01	2.54E-02	7.16E-01	2.60E-02	5.66E+01	4.78E+01	-5.63E-01	-9.34E+00
**PBC**	**PS**	7.45E-03	2.89E-03	3.52E-01	4.78E-02	2.18E-01	1.95E-02	6.15E-01	1.64E-02	4.70E+01	3.82E+01	1.46E+01	5.81E+00
**PBC**	**RA**	1.39E-02	2.61E-03	5.35E-01	6.41E-02	3.50E-01	2.54E-02	6.80E-01	2.46E-02	7.09E+01	6.22E+01	1.59E+00	-7.20E+00
**PBC**	**SLE**	3.26E-02	2.47E-03	4.86E-01	4.55E-02	3.38E-01	2.18E-02	6.62E-01	1.65E-02	8.88E+01	8.00E+01	1.96E+00	-6.81E+00
**PBC**	**SS**	6.92E-02	4.26E-03	8.61E-01	1.08E-01	6.44E-01	4.39E-02	8.56E-01	9.37E-02	1.11E+01	2.48E+00	5.25E-01	-8.05E+00
**PBC**	**TD1**	9.40E-02	3.84E-03	4.03E-01	4.42E-02	2.98E-01	2.85E-02	6.32E-01	1.54E-02	8.98E+01	8.10E+01	1.05E+01	1.70E+00
**PS**	**JIA**	2.94E-02	1.93E-03	6.52E-01	8.61E-02	4.82E-01	3.26E-02	7.28E-01	3.71E-02	1.12E+01	1.62E+00	3.45E+00	-6.13E+00
**PS**	**MS**	2.67E-03	1.81E-03	1.90E-01	1.97E-02	1.30E-01	1.49E-02	5.61E-01	6.38E-03	9.05E+01	8.10E+01	7.45E+00	-2.11E+00
**PS**	**RA**	-2.12E-04	1.85E-03	2.50E-01	3.44E-02	1.61E-01	1.68E-02	5.81E-01	1.13E-02	5.08E+01	4.12E+01	1.03E+01	6.87E-01
**PS**	**SLE**	1.36E-02	1.87E-03	1.06E-02	4.12E-02	3.86E-03	1.72E-02	5.03E-01	1.31E-02	5.36E+01	4.41E+01	2.16E+01	1.20E+01
**PS**	**SS**	3.51E-03	2.86E-03	3.64E-01	9.67E-02	1.66E-01	4.50E-02	6.19E-01	3.37E-02	1.06E+01	1.58E+00	5.92E+00	-3.10E+00
**RA**	**JIA**	1.37E-02	2.53E-03	8.84E-01	1.07E-01	6.11E-01	3.83E-02	8.71E-01	8.80E-02	8.83E+00	-7.37E-01	1.40E+00	-8.16E+00
**RA**	**MS**	3.53E-02	1.65E-03	2.94E-01	3.40E-02	2.25E-01	1.14E-02	5.95E-01	1.14E-02	8.26E+01	7.31E+01	6.67E+00	-2.90E+00
**RA**	**SLE**	2.56E-02	2.03E-03	6.82E-01	3.68E-02	5.58E-01	1.63E-02	7.39E-01	1.63E-02	3.89E+01	2.94E+01	5.40E+00	-4.16E+00
**RA**	**SS**	5.23E-02	3.40E-03	9.54E-01	7.12E-02	5.06E-01	6.08E-02	9.39E-01	7.56E-02	1.43E+00	-7.60E+00	-1.20E+00	-1.02E+01
**SLE**	**JIA**	1.20E-02	2.09E-03	7.27E-01	8.21E-02	5.32E-01	3.41E-02	7.61E-01	3.83E-02	1.54E+01	5.83E+00	6.79E-01	-8.89E+00
**SLE**	**MS**	7.03E-03	2.08E-03	3.78E-01	4.32E-02	2.61E-01	1.43E-02	6.24E-01	1.49E-02	3.38E+01	2.43E+01	1.31E+01	3.55E+00
**SLE**	**SS**	2.79E-02	5.07E-03	9.95E-01	1.18E-02	5.99E-01	9.51E-02	9.88E-01	2.88E-02	-1.28E+00	-1.03E+01	-1.92E+00	-1.09E+01
**TD1**	**JIA**	2.99E-02	1.71E-03	5.95E-01	9.22E-02	4.50E-01	2.37E-02	7.05E-01	3.93E-02	2.25E+01	1.30E+01	2.76E+00	-6.80E+00
**TD1**	**MS**	6.49E-02	2.06E-03	2.63E-01	3.11E-02	1.60E-01	1.61E-02	5.85E-01	1.03E-02	8.64E+01	7.68E+01	3.86E+00	-5.67E+00
**TD1**	**PS**	1.12E-02	2.48E-03	3.01E-01	4.69E-02	1.42E-01	1.74E-02	5.97E-01	1.56E-02	5.29E+01	4.34E+01	3.57E+01	2.61E+01
**TD1**	**RA**	5.09E-02	2.27E-03	5.55E-01	6.22E-02	3.31E-01	1.23E-02	6.88E-01	2.40E-02	6.56E+01	5.60E+01	5.37E+00	-4.19E+00
**TD1**	**SLE**	1.48E-02	2.24E-03	6.33E-01	6.75E-02	3.80E-01	1.86E-02	7.19E-01	2.85E-02	3.18E+01	2.22E+01	8.08E+00	-1.47E+00
**TD1**	**SS**	4.83E-02	3.10E-03	3.82E-01	1.88E-01	1.05E-01	4.26E-02	6.28E-01	6.86E-02	7.73E+00	-1.30E+00	2.25E+01	1.34E+01
**UC**	**AITD**	-3.88E-03	2.47E-03	-1.42E-03	3.46E-02	-1.24E-03	1.47E-02	5.00E-01	1.10E-02	1.19E+02	1.10E+02	3.00E+01	2.05E+01
**UC**	**CD**	2.86E-01	1.85E-03	8.44E-01	4.87E-02	6.65E-01	1.20E-02	8.22E-01	2.80E-02	5.41E+01	4.46E+01	2.92E+01	1.97E+01
**UC**	**JIA**	1.09E-01	1.40E-03	6.99E-01	6.83E-02	5.24E-01	2.87E-02	7.48E-01	3.27E-02	9.02E+00	-5.20E-01	2.70E+00	-6.85E+00
**UC**	**MG**	2.36E-03	2.39E-03	4.37E-01	1.70E-01	1.35E-01	4.26E-02	6.47E-01	6.21E-02	2.75E+00	-6.77E+00	1.03E+01	8.24E-01
**UC**	**MS**	5.55E-02	2.11E-03	5.15E-01	4.26E-02	3.76E-01	1.56E-02	6.73E-01	1.60E-02	3.09E+01	2.14E+01	1.00E+01	5.20E-01
**UC**	**PBC**	1.61E-01	3.82E-03	4.63E-01	6.50E-02	3.24E-01	3.54E-02	6.54E-01	2.46E-02	4.60E+01	3.72E+01	6.12E+00	-2.63E+00
**UC**	**PS**	8.03E-03	1.88E-03	3.55E-01	4.43E-02	2.30E-01	1.96E-02	6.16E-01	1.54E-02	6.29E+01	5.33E+01	1.70E+01	7.44E+00
**UC**	**RA**	2.35E-02	2.26E-03	3.18E-01	4.18E-02	2.06E-01	1.69E-02	6.03E-01	1.41E-02	3.49E+01	2.54E+01	1.16E+01	2.03E+00
**UC**	**SLE**	1.93E-03	2.09E-03	2.85E-01	4.83E-02	1.33E-01	1.94E-02	5.92E-01	1.61E-02	2.70E+01	1.74E+01	3.27E+01	2.31E+01
**UC**	**SS**	4.87E-02	4.14E-03	7.41E-01	2.17E-01	2.30E-01	6.09E-02	7.92E-01	1.16E-01	8.40E-01	-8.16E+00	7.41E+00	-1.59E+00
**UC**	**TD1**	6.78E-02	2.60E-03	4.18E-01	4.31E-02	2.39E-01	1.97E-02	6.38E-01	1.50E-02	6.10E+01	5.14E+01	1.51E+01	5.56E+00

Supplementary Table 9. Specific genes in factor 1 and factor 4 disorders. Genes that are also involved in monogenic immune disorders are highlighted in bold and asterix*.

**Factor 1**	**Factor 4**							
AF131215.5	AC010441.1	CCDC36	GMPPB	KIAA1841	NR5A2	RP11-201K10.3	SMIM3	ZBTB38
ARL17B	ADCY7	CCL2	GPR35	**KLHDC8B***	NXPE4	RP11-293M10.1	SNAPC4	ZFP36L2
BANK1	ADO	CDC37	GPR65	LAMB2	OTUD3	RP11-342M21.2	SNX20	ZFP90
CDHR5	AHSA2	CDHR4	GPSM1	LIME1	P4HA2	RP11-3B7.1	SOX6	ZGPAT
CSK	AL161450.1	CELSR3	GPX1	LNPEP	PAPD5	RP4-583P15.14	STAT5A	ZNF365
CYP1A2	AMIGO3	CENPO	GPX4	LRRK2	PARK7	RP4-583P15.15	**STAT5B***	
DEAF1	AMT	CREM	HCN3	LTBR	PDLIM4	RPL37	STMN3	
DRD4	APEH	CSF2	HEATR3	MAP3K8	PKLR	RSPO3	SULT1A1	
FAM219B	ARFRP1	CTD-2260A17.2	HSPA6	MIER1	PLA2G2E	**RTEL1***	SYNGR1	
GRB2	ASH1L	CUL2	IL17REL	MMP9	PLAU	RTEL1-TNFRSF6B	TCTA	
**IKZF3***	ATG16L1	CYLD	IL18R1	MON1A	PLCL1	SAG	THADA	
**IRF7***	ATP6V0A1	DAG1	IL18RAP	MST1	PMPCA	SBNO2	TMCO4	
ITGAM	BSN	DALRD3	IL1RL1	MST1R	PRKAA1	SCAMP3	TNFRSF6B	
ITGAX	C11orf30	DGKD	IMPDH2	MSTO1	PTGER4	SDCCAG3	TNFSF15	
MPI	C20orf62	DNLZ	INPP5E	MUC19	PTRF	SEC16A	TRAIP	
**NCF2***	C3orf62	EGR2	INSL4	NCKIPSD	QRICH1	SH2B1	TSPAN14	
PDHB	C3orf84	ERAP2	INSL6	NDFIP1	RABEP2	SLC22A4	TTC33	
PHRF1	C5orf56	ERRFI1	IP6K1	NICN1	RBM5	SLC22A5	UBA7	
PXK	C9orf163	FDPS	IP6K2	NKD1	RBM6	SLC26A6	USP34	
SMG7	CACNA1S	FOSL2	**IRF1***	**NOD2***	RFT1	SLC2A4RG	USP4	
ST8SIA4	CAMKV	GCKR	IRGM	NOTCH1	RHOA	SLC9A4	WDR6	
WDFY4	**CARD9***	GMEB2	**JAK2***	NPIPB8	RNF123	SMAD3	YY1AP1	

Supplementary Table 10. Specific Gene-sets in different factors.

**Factor 1**	**Factor 3**
BIOCARTA_NO2IL12_PATHWAY	GOBP_INNATE_IMMUNE_RESPONSE
REACTOME_SIGNALING_BY_CSF1_M_CSF_IN_MYELOID_CELLS	WP_CANCER_IMMUNOTHERAPY_BY_PD1_BLOCKADE
WP_GENES_ASSOCIATED_WITH_THE_DEVELOPMENT_OF_RHEUMATOID_ARTHRITIS	ZHENG_FOXP3_TARGETS_UP
PID_TCR_PATHWAY	REACTOME_CYTOKINE_SIGNALING_IN_IMMUNE_SYSTEM
GOBP_LEUKOCYTE_MEDIATED_IMMUNITY	GOBP_POSITIVE_REGULATION_OF_RNA_METABOLIC_PROCESS
GOBP_IMMUNE_EFFECTOR_PROCESS	OSMAN_BLADDER_CANCER_DN
GOBP_POSITIVE_REGULATION_OF_LEUKOCYTE_MEDIATED_IMMUNITY	GOBP_B_CELL_PROLIFERATION
BIOCARTA_IL12_PATHWAY	GOBP_T_CELL_ACTIVATION_INVOLVED_IN_IMMUNE_RESPONSE
GOMF_TRANSFERRIN_RECEPTOR_BINDING	REACTOME_INTERFERON_GAMMA_SIGNALING
	GOBP_TOLERANCE_INDUCTION
	GOBP_T_CELL_PROLIFERATION
	GOBP_RESPONSE_TO_INTERLEUKIN_17
	GOBP_T_HELPER_17_TYPE_IMMUNE_RESPONSE
	GOBP_CD4_POSITIVE_ALPHA_BETA_T_CELL_ACTIVATION
	GOBP_POSITIVE_REGULATION_OF_ACTIVATION_OF_JANUS_KINASE_ACTIVITY
	WP_TCELL_ANTIGEN_RECEPTOR_TCR_PATHWAY_DURING_STAPH_AUR_INF
**Out of factors**	
GOBP_IMMUNE_RESPONSE_REGULATING_CELL_SURFACE_RECEPTOR_SIGNALING_PATHWAY	
GOBP_CYTOKINE_PRODUCTION	
WORSCHECH_TUMOR_EVASION_AND_TOLEROGENICITY_DN	
GOBP_POSITIVE_REGULATION_OF_FC_RECEPTOR_MEDIATED_STIMULATORY_SIGNALING_PATHWAY	
GOBP_T_CELL_DIFFERENTIATION_IN_THYMUS	
GOBP_POSITIVE_REGULATION_OF_LYMPHOCYTE_ACTIVATION	
GOBP_POSITIVE_REGULATION_OF_INTERLEUKIN_2_PRODUCTION	
GOBP_POSITIVE_REGULATION_OF_HEMOPOIESIS	
GOBP_POSITIVE_REGULATION_OF_LYMPHOCYTE_DIFFERENTIATION	
GOBP_ADAPTIVE_IMMUNE_RESPONSE_BASED_ON_SOMATIC_RECOMBINATION_OF_IMMUNE_RECEPTORS_BUILT_FROM_IMMUNOGLOBULIN_SUPERFAMILY_DOMAINS	
GOBP_NEGATIVE_REGULATION_OF_T_CELL_DIFFERENTIATION	
GOBP_CD8_POSITIVE_ALPHA_BETA_T_CELL_DIFFERENTIATION	
KEGG_T_CELL_RECEPTOR_SIGNALING_PATHWAY	
GOBP_REGULATION_OF_CD4_POSITIVE_ALPHA_BETA_T_CELL_ACTIVATION	
BASSO_CD40_SIGNALING_UP	
WP_EXTRAFOLLICULAR_AND_FOLLICULAR_B_CELL_ACTIVATION_BY_SARSCOV2	
GOBP_REGULATION_OF_IMMUNE_SYSTEM_PROCESS	
BOSCO_ALLERGEN_INDUCED_TH2_ASSOCIATED_MODULE	
GOBP_INTERLEUKIN_6_MEDIATED_SIGNALING_PATHWAY	

**Factor 4**
GOBP_TYPE_II_INTERFERON_PRODUCTION
WP_IL1_AND_MEGAKARYOCYTES_IN_OBESITY
KEGG_LEISHMANIA_INFECTION
LI_INDUCED_T_TO_NATURAL_KILLER_UP
PID_IL5_PATHWAY. GOBP_B_CELL_ACTIVATION
GOBP_POSITIVE_REGULATION_OF_INTERLEUKIN_12_PRODUCTION
REACTOME_SIGNALING_BY_CSF3_G_CSF
PID_IL12_STAT4_PATHWAY

GOBP_POSITIVE_REGULATION_OF_T_HELPER_1_TYPE_IMMUNE_RESPONSE
GOBP_REGULATION_OF_T_HELPER_1_TYPE_IMMUNE_RESPONSE
GOBP_REGULATION_OF_RESPONSE_TO_EXTERNAL_STIMULUS
GOBP_POSITIVE_REGULATION_OF_CELL_CELL_ADHESION
GOBP_REGULATION_OF_CATALYTIC_ACTIVITY
GOBP_REGULATION_OF_CELL_POPULATION_PROLIFERATION
GOBP_REGULATION_OF_DNA_BINDING_TRANSCRIPTION_FACTOR_ACTIVITY
WP_TCELL_ACTIVATION_SARSCOV2
GOBP_ADAPTIVE_IMMUNE_RESPONSE
WP_IL18_SIGNALING_PATHWAY. WP_IL13_SIGNALING_PATHWAY
WP_SELECTIVE_EXPRESSION_OF_CHEMOKINE_RECEPTORS_DURING_TCELL_POLARIZATION
GOBP_POSITIVE_REGULATION_OF_PHOSPHORYLATION
SHIN_B_CELL_LYMPHOMA_CLUSTER_3
WP_COVID19_ADVERSE_OUTCOME_PATHWAY
GOBP_POSITIVE_REGULATION_OF_TUMOR_NECROSIS_FACTOR_SUPERFAMILY_CYTOKINE_PRODUCTION
GOBP_NEGATIVE_REGULATION_OF_INFLAMMATORY_RESPONSE
UZONYI_RESPONSE_TO_LEUKOTRIENE_AND_THROMBIN
GOBP_REGULATION_OF_ADAPTIVE_IMMUNE_RESPONSE
PID_IL27_PATHWAY. PID_IL23_PATHWAY
GOBP_POSITIVE_REGULATION_OF_PHOSPHORUS_METABOLIC_PROCESS
BRUNEAU_HEART_GREAT_VESSELS_AND_VALVULOGENESIS
PID_REG_GR_PATHWAY. GOPB_HEMOPOESIS
REACTOME_INTERLEUKIN_4_AND_INTERLEUKIN_13_SIGNALING
GOBP_T_HELPER_1_TYPE_IMMUNE_RESPONSE
GOBP_POSITIVE_REGULATION_OF_CELL_COMMUNICATION
GOBP_POSITIVE_REGULATION_OF_ADAPTIVE_IMMUNE_RESPONSE
GOBP_ALPHA_BETA_T_CELL_PROLIFERATION
GOBP_MATURE_B_CELL_DIFFERENTIATION
GOBP_MATURE_B_CELL_DIFFERENTIATION_INVOLVED_IN_IMMUNE_RESPONSE

Supplementary Table 11. Tissue specificity analysis by MAGMA. For each disease, genes identified in MAGMA analysis were tested for tissue specific expression using gene expression data from 54 tissue types from GTEx v8.

Diseases	Tissue	P-value
Autoimmune thyroiditis	Spleen	2.6e-19
	Whole blood	4.2e-16
	Cells EBV-transformed lymphocytes	1.8e-15
	Small intestine terminal ileum	2.7e-13
	Lung	6.63e-09
Celiac disease	Whole blood	1.7e-04
	Small intestine terminal ileum	5.5e-04
Crohn's disease	Whole blood	2.5e-13
	Spleen	2.0e-12
	Small intestine terminal ileum	1.4e-10
	Lung	1.1e-08
	Cells EBV-transformed lymphocytes	6.6e-06
	Colon transverse	1.7e-04
Juvenile idiopathic arthritis	Whole blood	6.0e-05
	Spleen	1.2e-04
Multiple sclerosis	Spleen	5.9e-16
	Cells EBV-transformed lymphocytes	2.1e-11
	Whole blood	2.3e-11
	Small intestine terminal ileum	1.5e-07
	Lung	2.6e-06
Myasthenia gravis	Lung	7.1e-04
Primary biliary cholangitis	Spleen	1.0e-09
	Whole blood	2.8e-08
	Small intestine terminal ileum	4.5e-08
	Cells EBV-transformed lymphocytes	1.7e-07
	Lung	5.8e-05
Primary sclerosing cholangitis	No significant results	
Primary Sjogren's syndrome	No significant results	
Psoriasis (including Psoriatic arthritis)	Spleen	1.7e-08
	Whole blood	2.2e-08
	Small intestine terminal ileum	3.7e-06
	Cells EBV-transformed lymphocytes	1.6e-05
	Lung	5.7e-04
Rheumatoid arthritis	Whole blood	1.6e-10
	Spleen	5.0e-10
	Cells EBV-transformed lymphocytes	2.1e-09
	Lung	4.2e-07
	Small intestine terminal ileum	1.3e-05
Systemic lupus erythematosus	Spleen	2.3e-09
	Cells EBV-transformed lymphocytes	6.6e-08
	Whole blood	2.1e-06
	Small intestine terminal ileum	1.8e-04
Systemic sclerosis	Small intestine terminal ileum	1.6e-06
	Whole blood	7.3e-06
	Cells EBV-transformed lymphocytes	2.7e-04
	Spleen	4.4e-04
Type 1 diabetes	Spleen	1.0e-15
	Whole blood	6.7e-11
	Lung	7.0e-11
	Small intestine terminal ileum	2.0e-08
	Cells EBV-transformed lymphocytes	6.2e-08
Ulcerative colitis	Spleen	1.5e-04
	Whole blood	3.1e-04
	Small intestine terminal ileum	8.7e-04

Supplementary Fig. 1. Heatmap of genome-wide genetic correlations across 15 immune-mediated diseases. Numbers in the cells show genetic correlations estimated using linkage disequilibrium score regression (LDSC). Blue color indicates positive correlation, and red color indicates negative correlations. Significant correlations surviving Bonferroni correction are marked with asterisk (*).

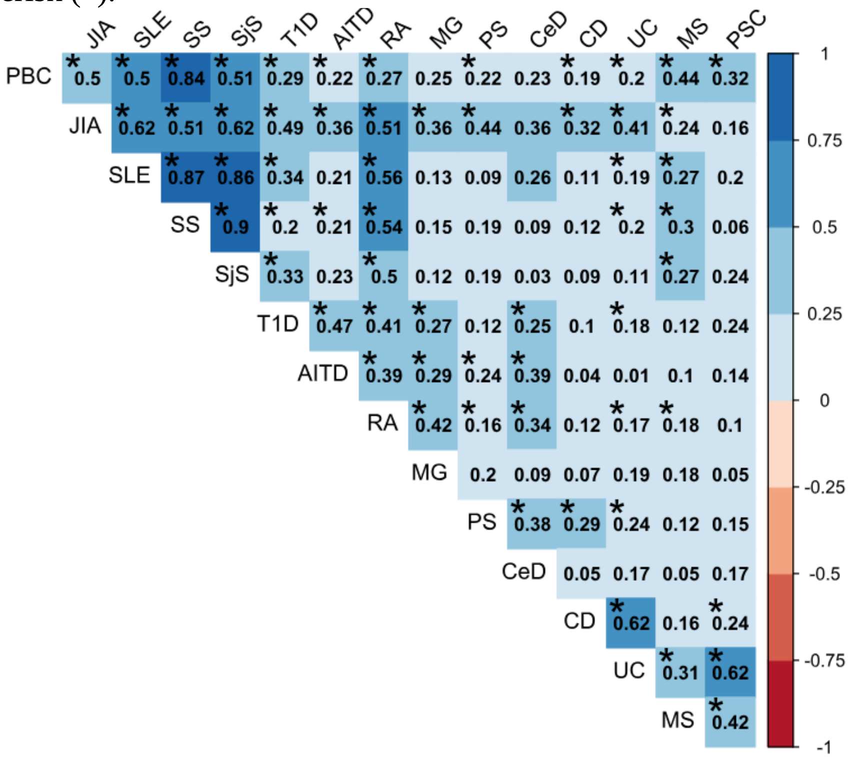

Supplementary Fig. 2. Genetic overlap beyond genetic correlations. Fraction of disease-influencing genetic variants shared between two diseases with respect to the less polygenic disease in the pair estimated with Bivariate Gaussian causal mixture model (MiXeR) for 15 immune-mediated diseases.

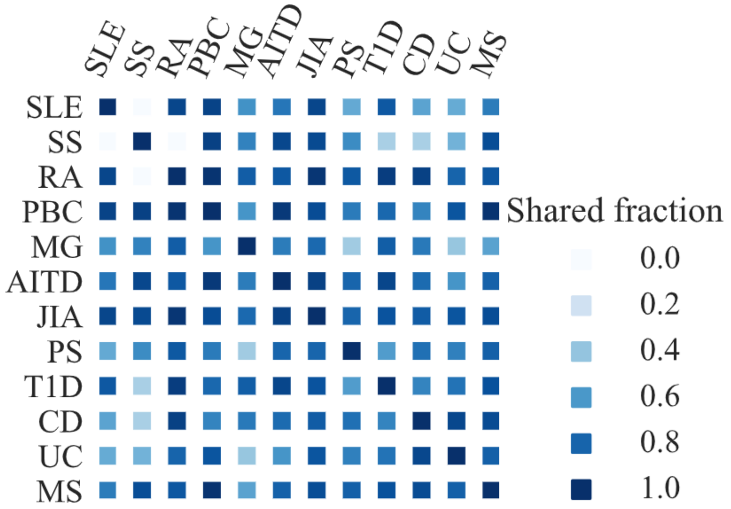

Supplementary Fig. 3. Local genetic correlations across immune-mediated diseases. Heatmap of local genetic correlations estimated with LAVA across 2495 LD-unlinked genetic regions. The color indicates average correlation across all positively (top right) and negatively (bottom left) correlated regions. Numbers in cells indicate the number of regions with significant local generic correlation. The absence of number indicates that there were no regions with significant correlation. The correlation range is represented on the scale with a color gradient changing from -1 to 1.

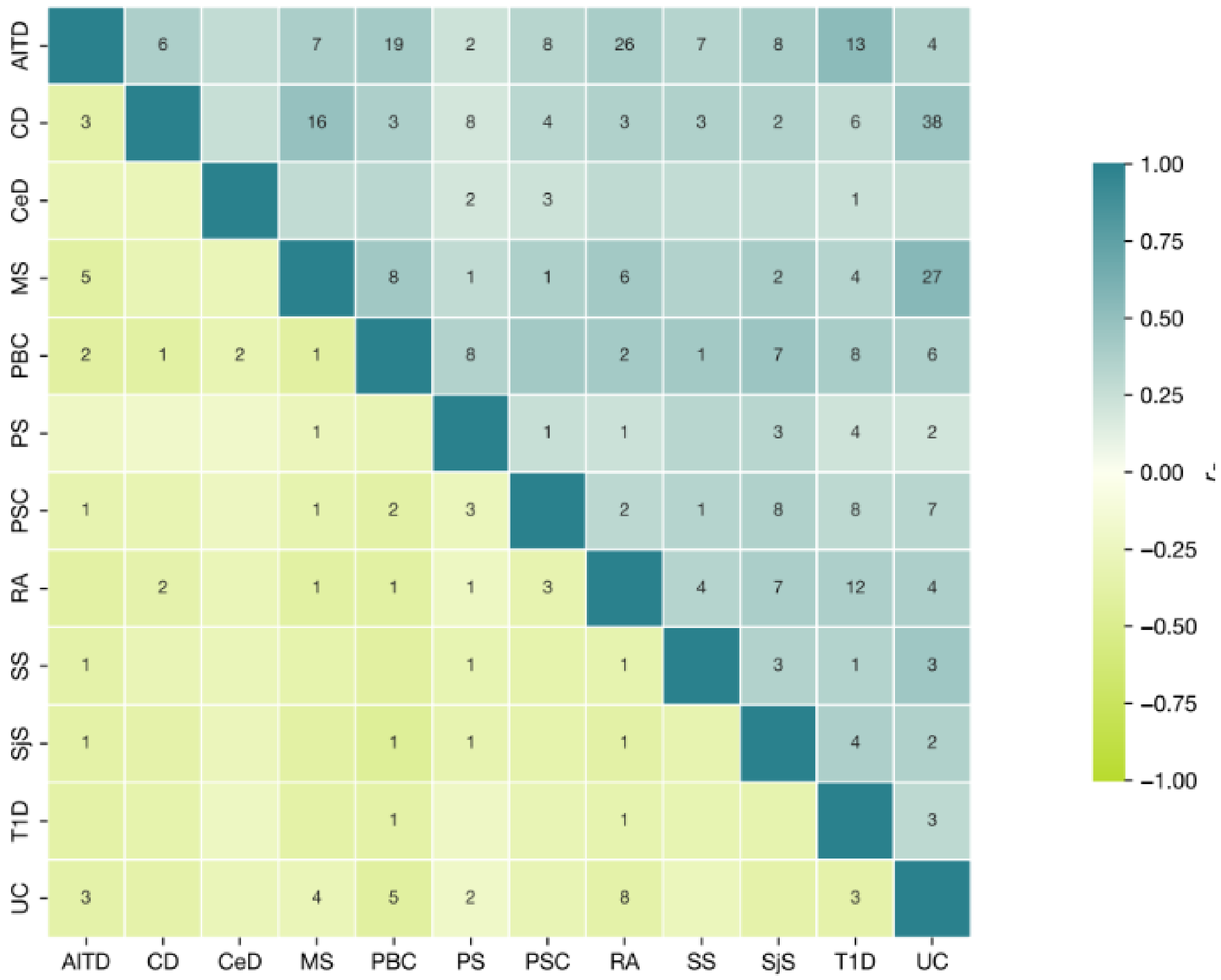

Supplementary Fig. 4. Genes shared between five or more immune-mediated diseases. For each disease, associated genes were identified using MAGMA.

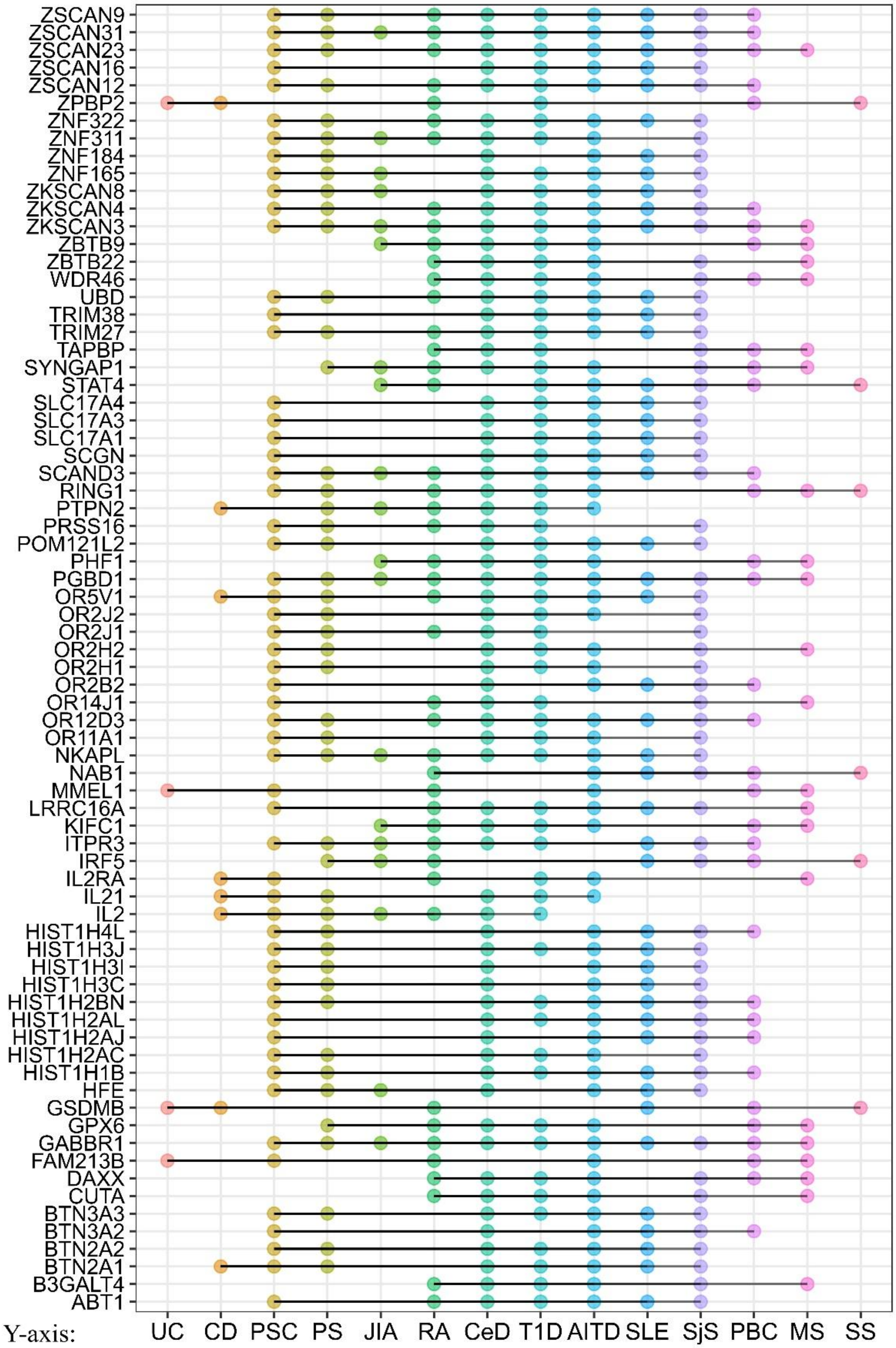

Y-axis:

Gene names

Supplementary Fig. 6. MAGMA-identified genes uniquely associated with diseases involved in "autoimmune" factor 1 (A) and "autoinflammatory" factor 4 (B) and linked to monogenic immune disorders and blood syndrome. A. For factor 1 we marked genes linked to monogenic immune diseases with light blue circles. B. For factor 4 we showed genes only linked to monogenic diseases. Lines between genes show the involvement in molecular networks (the StringApp for Cytoscape, String Protein Database).

A. Factor 1.

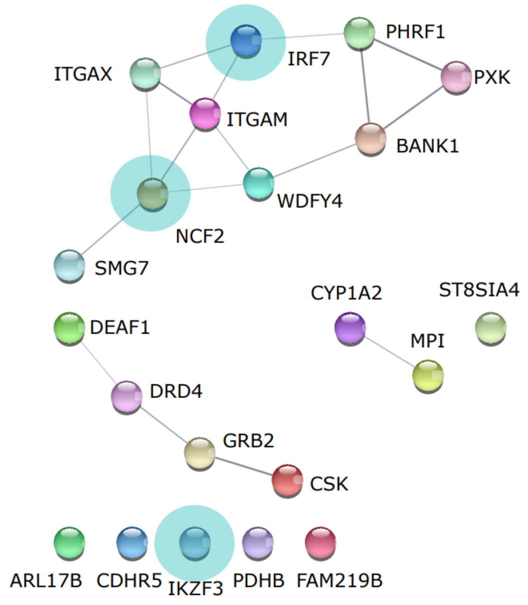

B. Factor 4.

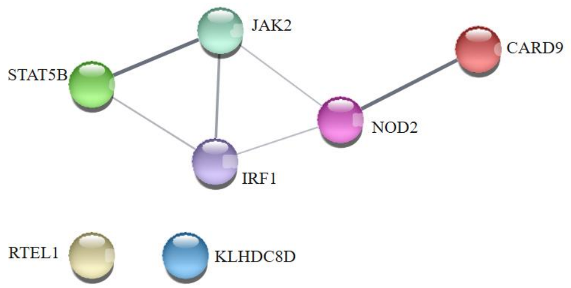

Janus kinase 2 (*JAK2*) is involved in different blood disorders. *KLHDC8B* is involved in Hodgkin lymphoma. *RTEL1* in inborn error failure. *IRF1* and *CARD9* engage in different types of immunodeficiencies [Rosain et al. 2023, Wang et al. 2019, Salipante et al. 2009]. The *NOD2* gene plays a role in Blau syndrome, a rare autosomal dominant autoinflammatory syndrome classified as an autoinflammatory phenotype [Tangye et al. 2022]. S*TAT5B* gene is the monogenic cause of autosomal dominant growth hormone insensitivity syndrome with immune dysregulation-2.

Supplementary Table 12 with figures. Top twenty regions from Local Analysis of [co]Variant Association (LAVA analysis with top significant local genetic correlations. We provided the locus number, position on the chromosome and genes mapped to this region.

In the table, Phenotype1 and Phenotype2 denote two traits for which local genetic correlations were analyzed at the genomic locus. Rho parameter represents the correlation coefficient measuring the strength and direction of the genetic relationship between the two phenotypes within the specific region. CI.lower and CI.upper indicate the lower and upper bounds of the confidence interval for the rho value. R2: R2 represents the proportion of variance in one phenotype that is explained by the variance in the other phenotype, attributed to shared genetic factors in the specific genomic region being studied. P: p-value of the observed correlation (rho) between the phenotypes.

The figure visualized the significant positive and negative correlation within this locus. We used blue color for negative correlations and red color for positive correlations. Abbreviations for the disorders are the same as in main publication.

**Top 1. Locus 964**<br>chr6: 32539568 - 32586784<br>Genes: *HLA-DRB1*						
**Phenotype1**	**Phenotype2**	**Rho**	**CI.lower**	**CI.upper**	**R2**	**P**
MS	UC	0.56	0.49	0.64	0.32	1.54E-36
SS	AITD	0.44	0.29	0.58	0.20	1.87E-07
CeD	PBC	-1.00	-1.00	-0.97	1.00	4.21E-12
T1D	PBC	0.76	0.68	0.83	0.58	1.17E-33
SjS	PBC	-0.61	-0.72	-0.49	0.38	4.81E-16
PS	PSC	-0.38	-0.47	-0.30	0.15	1.57E-15
PBC	PSC	-0.70	-0.81	-0.58	0.50	5.09E-17
UC	RA	-0.50	-0.57	-0.42	0.25	1.32E-31
SS	PSC	0.43	0.26	0.59	0.18	3.33E-06
RA	PSC	-0.41	-0.48	-0.34	0.17	1.99E-24
AITD	PBC	0.55	0.43	0.67	0.31	6.90E-13
UC	PSC	0.27	0.18	0.37	0.07	3.87E-08
AITD	UC	-0.41	-0.49	-0.33	0.17	2.81E-19
RA	PBC	0.81	0.74	0.88	0.66	2.48E-39
UC	PS	-0.66	-0.78	-0.52	0.43	3.82E-14
MS	PBC	-0.84	-0.92	-0.77	0.71	5.11E-31
AITD	PSC	0.53	0.45	0.61	0.28	9.98E-30
UC	PBC	-0.87	-0.95	-0.78	0.75	1.16E-26

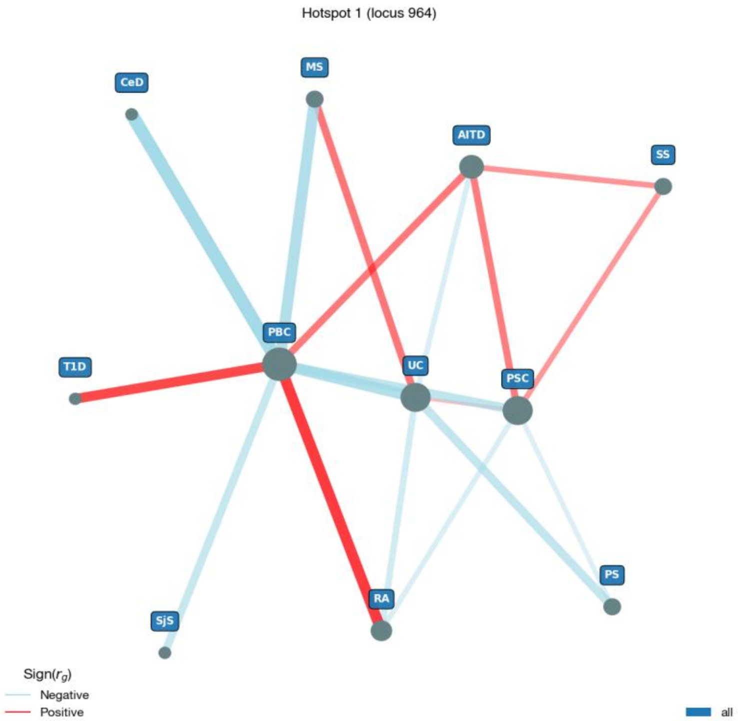
Hotspot 1 (locus 964)
CeD
MS
AITD
SS
T1D
PBC
UC
PSC
SjS
RA
PS
Sign(r_g)
Negative
Positive
all

**Top 2. Locus 966**<br>chr6: 32629240 - 32682213<br>Genes: *HLA-DQB1*						
**Phenotype1**	**Phenotype2**	**Rho**	**CI.lower**	**CI.upper**	**R2**	**P**
PS	PBC	0.63	0.51	0.74	0.39	3.34E-16
MS	UC	0.44	0.37	0.52	0.20	9.38E-26
SS	AITD	0.43	0.31	0.54	0.18	1.87E-11
SS	PBC	0.33	0.22	0.44	0.11	6.12E-08
PBC	PSC	-0.46	-0.54	-0.38	0.21	1.16E-25
UC	RA	-0.23	-0.31	-0.15	0.05	6.19E-09
SjS	PSC	0.87	0.83	0.91	0.76	4.58E-128
RA	PSC	-0.53	-0.59	-0.48	0.28	7.04E-65
SS	RA	0.41	0.32	0.50	0.17	2.25E-15
MS	PSC	0.80	0.76	0.84	0.64	1.56E-155
UC	PSC	0.31	0.22	0.40	0.09	7.71E-09
SS	UC	0.33	0.22	0.45	0.11	1.02E-07
RA	PBC	0.27	0.20	0.34	0.07	7.57E-14
MS	PBC	-0.37	-0.43	-0.30	0.13	9.29E-23
AITD	PSC	-0.23	-0.32	-0.15	0.05	1.86E-07

Hotspot 2 (locus 966)

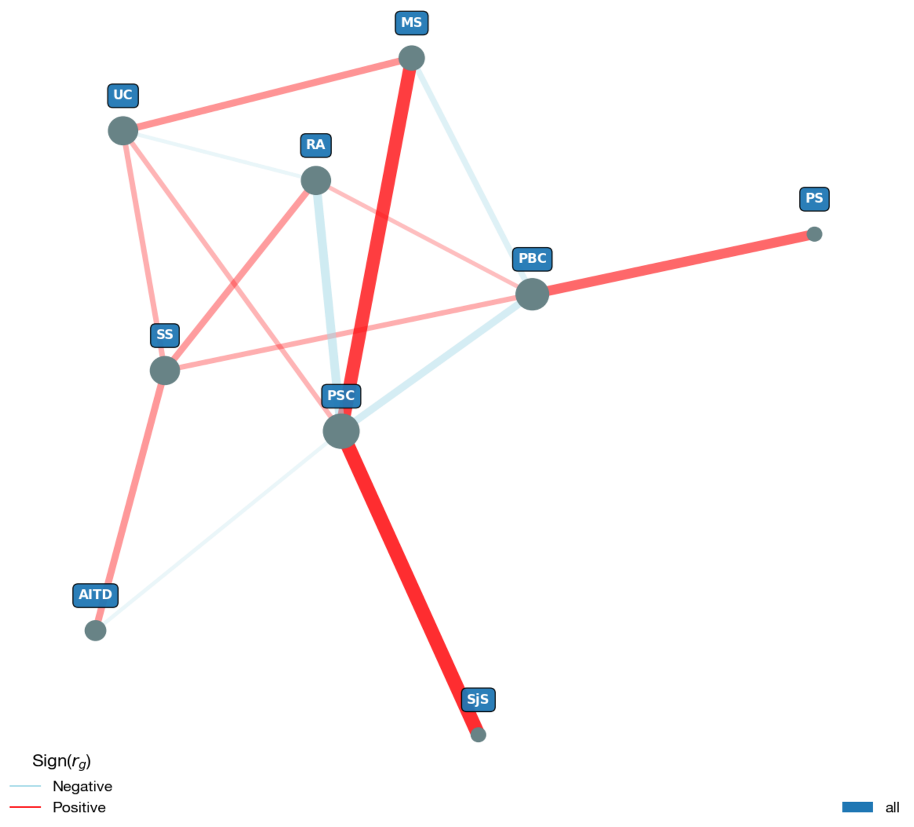

**Top3. Locus 374**<br>chr2: 191051955 – 193033982<br>Genes: *C2orf88; HIBCH; INPP1; MFSD6; TMEM194B; NAB1; GLS; STAT1; STAT4; MYO1B; NABP1; SDPR; TMEFF2*						
**Phenotype1**	**Phenotype2**	**Rho**	**CI.lower**	**CI.upper**	**R2**	**P**
SS	AITD	0.59	0.43	0.76	0.35	3.37E-10
T1D	SjS	0.46	0.29	0.64	0.22	1.38E-06
T1D	PBC	0.57	0.41	0.74	0.33	4.04E-09
SS	PBC	0.68	0.52	0.83	0.46	7.62E-11
SjS	PBC	0.55	0.40	0.70	0.30	4.14E-10
SS	SjS	0.53	0.34	0.72	0.28	5.91E-07
T1D	RA	0.54	0.34	0.77	0.29	4.37E-07
SS	RA	0.86	0.68	1.00	0.74	5.05E-12
AITD	RA	0.76	0.59	0.98	0.58	8.75E-16
AITD	PBC	0.58	0.46	0.70	0.34	1.19E-16
AITD	SjS	0.69	0.58	0.81	0.48	1.33E-21
RA	PBC	0.76	0.59	0.95	0.59	3.27E-13
RA	SjS	0.63	0.46	0.81	0.40	2.23E-10

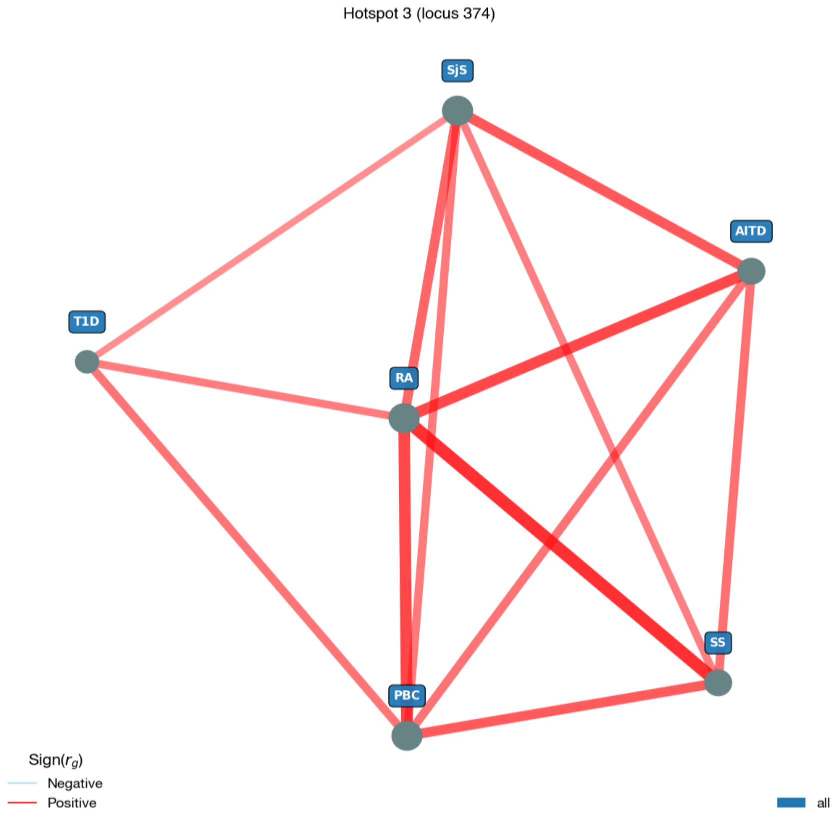

**Top 4. Locus 968**<br>chr6: 32897999 - 33194975<br>Genes: *HLA-DMB; HLA-DMA; BRD2; HLA-DOA; HLA-DPA1; HLA-DPB1; COL11A2; RXRB; SLC39A7; HSD17B8; RING1*						
**Phenotype1**	**Phenotype2**	**Rho**	**CI.lower**	**CI.upper**	**R2**	**P**
MS	UC	0.29	0.18	0.40	0.08	1.89E-07
SS	AITD	-0.25	-0.34	-0.15	0.06	7.60E-07
SS	PBC	0.27	0.17	0.37	0.07	4.25E-07
PS	PSC	-0.31	-0.39	-0.22	0.09	2.95E-11
MS	RA	-0.32	-0.36	-0.27	0.10	1.93E-48
SjS	PSC	0.79	0.73	0.84	0.62	1.09E-66
MS	AITD	-0.22	-0.28	-0.16	0.05	7.03E-12
RA	PSC	-0.29	-0.36	-0.22	0.08	5.54E-15
AITD	RA	0.40	0.36	0.46	0.16	3.56E-48
AITD	PBC	-0.31	-0.40	-0.23	0.10	6.18E-12
UC	PBC	-0.32	-0.45	-0.19	0.10	3.51E-06

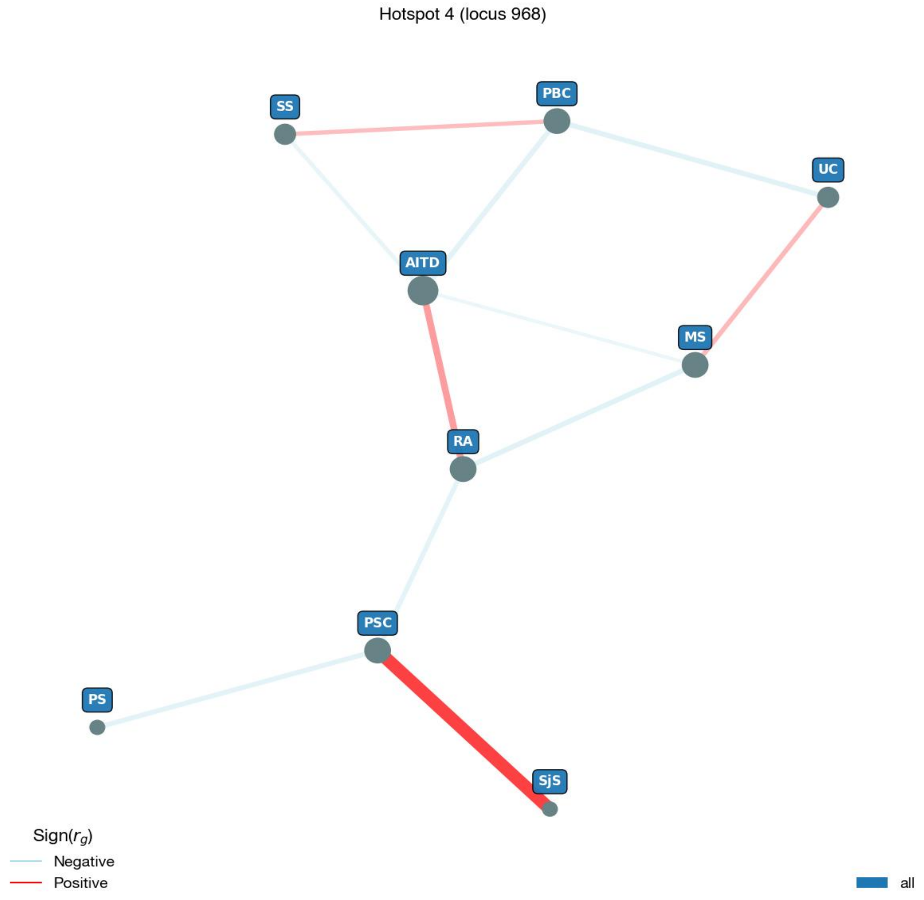

**Top 5. Locus 962**<br>chr6: 32208902 - 32454577<br>Genes: *C6orf10; BTNL2; LOC101929163; HLA-DRA*						
**Phenotype1**	**Phenotype2**	**Rho**	**CI.lower**	**CI.upper**	**R2**	**P**
MS	UC	0.18	0.12	0.24	0.03	5.09E-08
UC	RA	-0.21	-0.26	-0.16	0.04	3.52E-15
MS	RA	0.15	0.12	0.18	0.02	1.03E-31
MS	AITD	-0.14	-0.20	-0.08	0.02	6.95E-06
AITD	RA	0.36	0.31	0.41	0.13	7.04E-48
AITD	PBC	0.19	0.11	0.26	0.03	1.41E-06
AITD	UC	-0.19	-0.26	-0.13	0.04	6.86E-09
SS	UC	0.40	0.28	0.53	0.16	4.00E-09
RA	PBC	0.25	0.19	0.32	0.06	5.66E-14
MS	PBC	-0.32	-0.38	-0.25	0.10	3.31E-21
UC	PBC	-0.27	-0.36	-0.18	0.07	8.57E-09

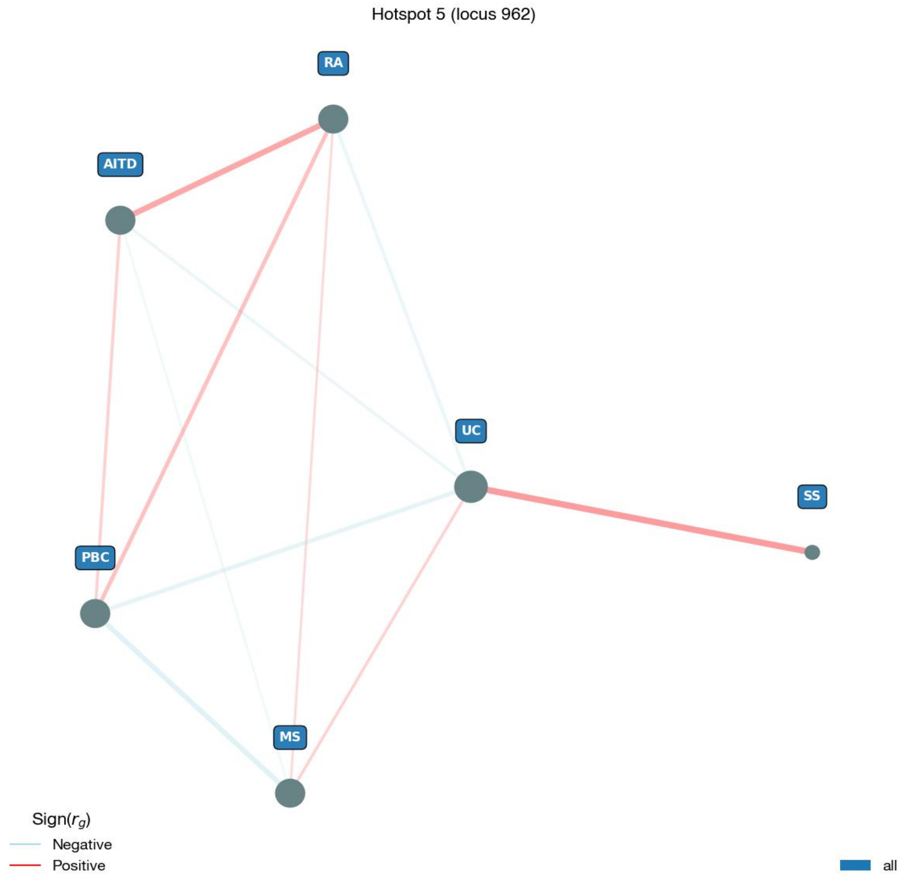

**Top 6. Locus 1207**<br>chr7: 127903739 - 128779173<br>Genes: *RBM28; PRRT4; IMPDH1; HILPDA; METTL2B; LOC101928451; FAM71F2; FAM71F1; CALU; OPN1SW; CCDC136; FLNC; ATP6V1F; LOC100130705; KCP; IRF5; TNPO3*						
**Phenotype1**	**Phenotype2**	**Rho**	**CI.lower**	**CI.upper**	**R2**	**P**
PS	PBC	0.46	0.28	0.65	0.21	7.10E-06
SS	PBC	0.85	0.73	0.97	0.72	2.03E-16
SjS	PBC	0.91	0.83	0.99	0.83	2.81E-32
SS	SjS	0.68	0.54	0.81	0.46	1.96E-14
SS	RA	0.70	0.50	0.90	0.49	4.74E-08
AITD	PBC	0.63	0.36	1.00	0.40	2.56E-05
AITD	SjS	0.80	0.51	1.00	0.63	1.03E-06
RA	PBC	0.80	0.65	0.97	0.65	6.26E-15
RA	SjS	0.95	0.80	1.00	0.90	2.88E-18

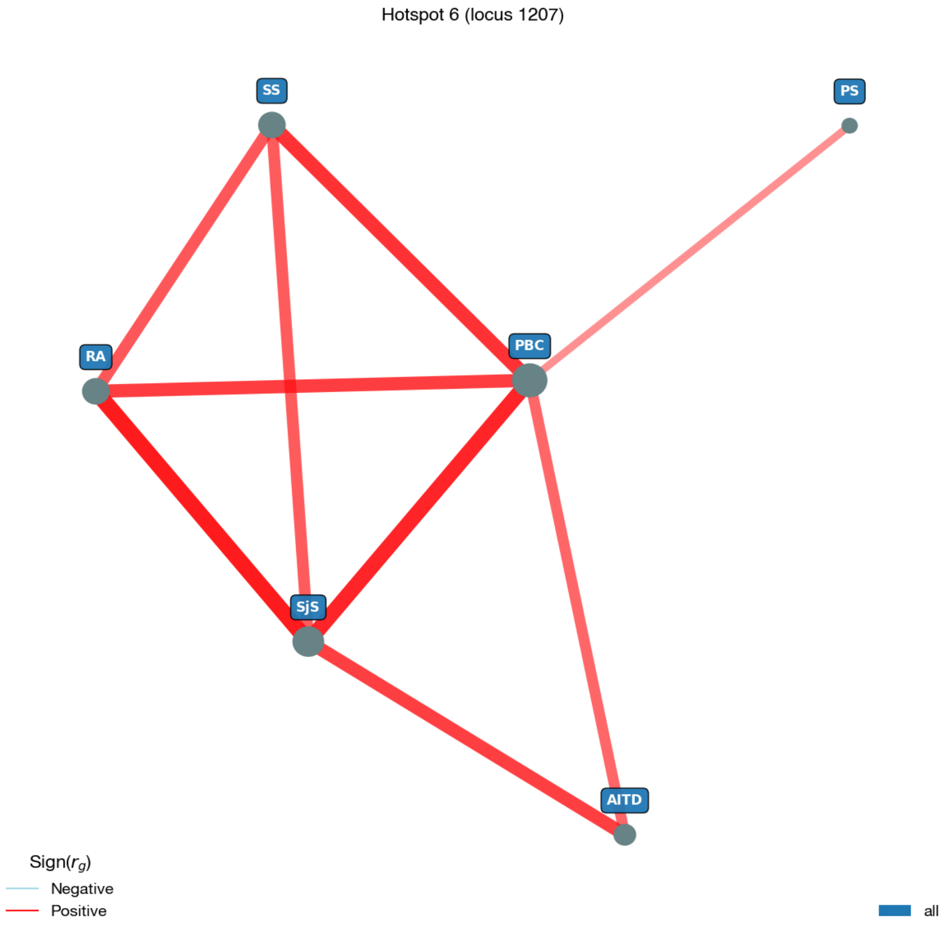

**Top 7. Locus 1841**<br>chr12: 111592382 - 113947983<br>Genes: *CUX2; FAM109A; SH2B3; ATXN2; BRAP; ACAD10; ALDH2; MAPKAPK5; TMEM116; ERP29; NAA25; TRAFD1; HECTD4; RPL6; PTPN11; RPH3A; OAS1; OAS3; OAS2; DTX1; RASAL1; CCDC42B; DDX54; RITA1; IQCD; TPCN1; SLC8B1; PLBD2; SDS; SDSL; LHX5*						
**Phenotype1**	**Phenotype2**	**Rho**	**CI.lower**	**CI.upper**	**R2**	**P**
T1D	PBC	0.81	0.62	1.00	0.65	1.96E-10
T1D	RA	0.89	0.59	1.00	0.79	6.52E-09
AITD	CD	0.54	0.30	1.00	0.29	1.57E-05
MS	T1D	0.90	0.51	1.00	0.81	3.56E-06
AITD	RA	0.83	0.53	1.00	0.69	2.80E-08
AITD	PBC	0.79	0.60	1.00	0.62	1.69E-10
AITD	T1D	1.00	0.94	1.00	1.00	4.91E-100
RA	PBC	0.97	0.63	1.00	0.93	3.30E-06
AITD	PSC	0.40	0.23	0.60	0.16	2.14E-05

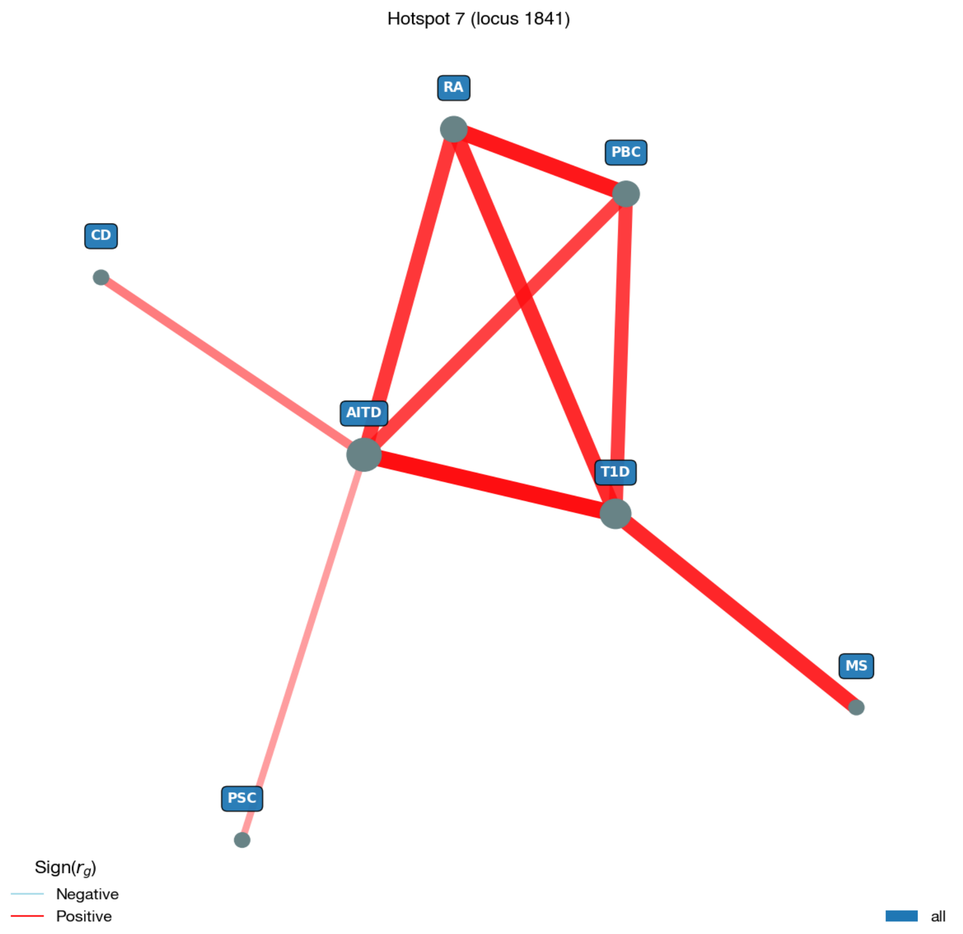

**Top 8. Locus 969**<br>chr6: 33194976 - 33864262<br>Genes: *VPS52; RPS18; B3GALT4; WDR46; PFDN6; RGL2; TAPBP; ZBTB22; DAXX; KIFC1; PHF1; CUTA; SYNGAP1; ZBTB9; BAK1; ITPR3; UQCC2; IP6K3; LEMD2; MLN*						
**Phenotype1**	**Phenotype2**	**Rho**	**CI.lower**	**CI.upper**	**R2**	**P**
PS	SjS	-0.23	-0.33	-0.13	0.05	6.71E-06
MS	RA	-0.30	-0.39	-0.21	0.09	4.96E-11
SjS	PSC	0.64	0.54	0.73	0.41	2.14E-25
T1D	PSC	0.24	0.16	0.33	0.06	6.66E-08
AITD	RA	0.37	0.30	0.44	0.14	2.61E-23
AITD	PBC	-0.29	-0.42	-0.17	0.09	7.65E-06
RA	PBC	-0.23	-0.32	-0.13	0.05	1.45E-06
RA	SjS	-0.29	-0.38	-0.20	0.08	2.18E-10
UC	PBC	-0.45	-0.63	-0.28	0.20	3.29E-07

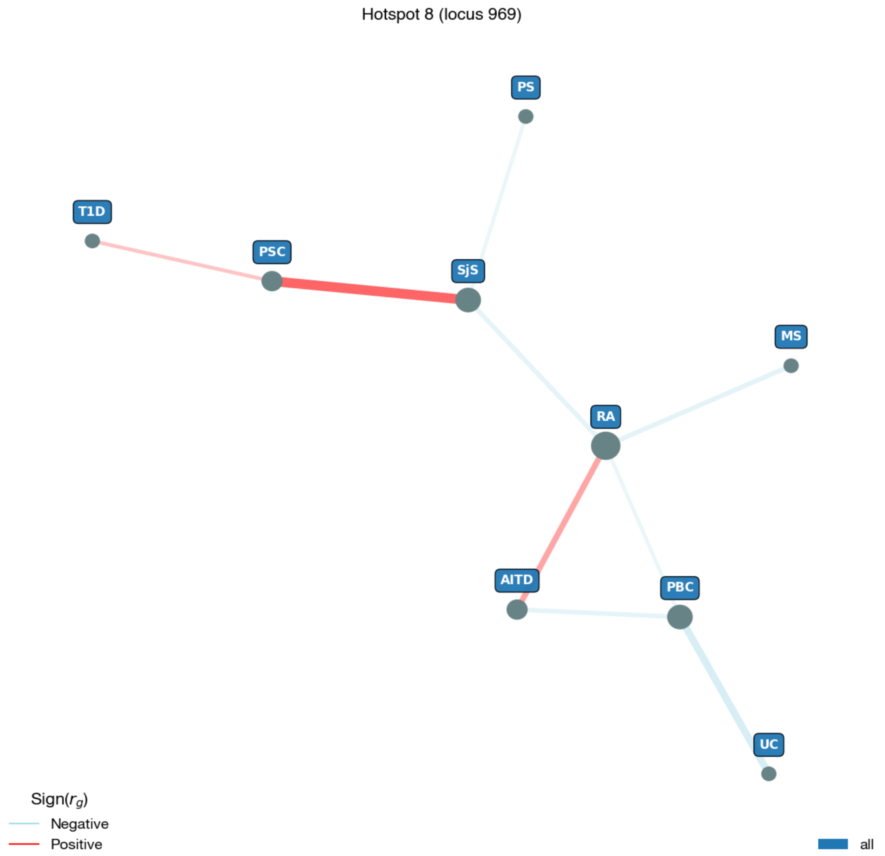

Top 9. Locus 2318
chr19: 10028841 - 11681978
Genes: *OLFM2; COL5A3; RDH8; C19orf66; ANGPTL6; PPAN; PPAN-P2RY11; P2RY11; EIF3G; DNMT1; S1PR2; MRPL4; ICAM1; ICAM4; ICAM5; ZGLP1; FDX1L; RAVER1; ICAM3; TYK2; CDC37; PDE4A; KEAP1; S1PR5; ATG4D; KRI1; CDKN2D; AP1M2; SLC44A2; ILF3; QTRT1; DNM2; TMED1; C19orf38; CARM1; YIPF2; C19orf52; SMARCA4; LDLR; SPC24; KANK2; DOCK6; C19orf80; TSPAN16; RAB3D; TMEM205; CCDC159; LPPR2; SWSAP1; EPOR; RGL3; CCDC151; PRKCSH; ELAVL3; ZNF653; ECSIT; CNN1; ELOF1*

Phenotype1	Phenotype2	Rho	CI.lower	CI.upper	R2	P
PS	SjS	0.54	0.39	0.70	0.29	3.72E-10
PS	PBC	0.42	0.25	0.58	0.17	2.98E-06
MS	UC	0.73	0.44	1.00	0.54	3.09E-06
T1D	PBC	0.64	0.40	0.91	0.42	3.83E-06
SjS	PBC	0.80	0.61	1.00	0.65	2.24E-10
T1D	RA	0.78	0.58	1.00	0.61	4.39E-11
MS	CD	0.50	0.28	0.76	0.25	2.16E-05
RA	PBC	0.61	0.41	0.81	0.37	2.28E-08
RA	SjS	0.66	0.49	0.83	0.43	1.09E-11

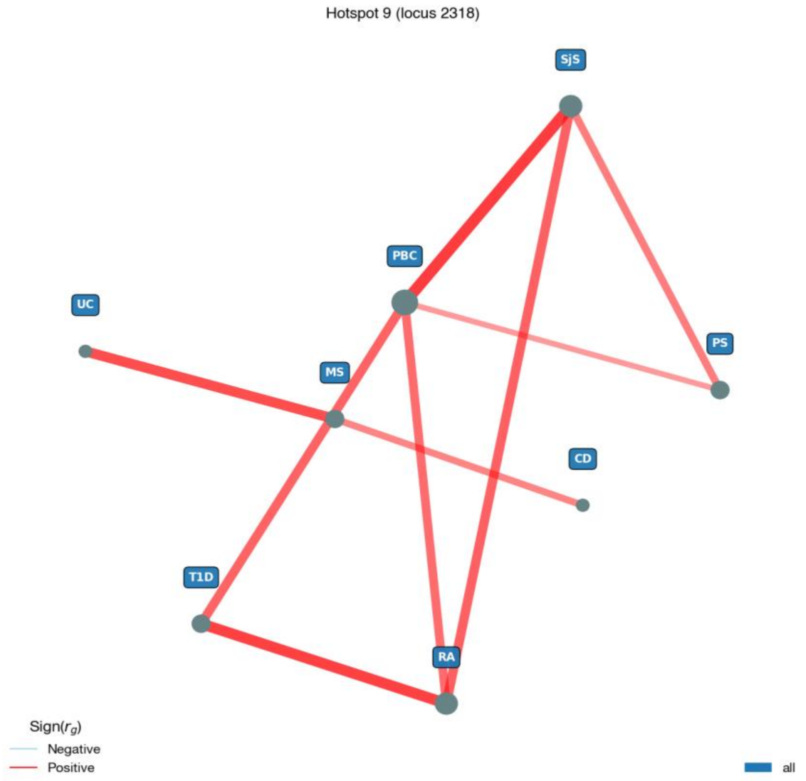

**Top 10. Locus 1492**<br>chr10: 5396243 - 6234718<br>Genes: *UCN3; TUBAL3; NET1; CALML5; CALML3; ASB13; FAM208B; GDI2; ANKRD16; FBXO18; IL15RA; IL2RA; RBM17; PFKFB3*						
**Phenotype1**	**Phenotype2**	**Rho**	**CI.lower**	**CI.upper**	**R2**	**P**
MS	UC	0.78	0.46	1.00	0.60	5.77E-06
T1D	RA	0.48	0.31	0.67	0.23	1.28E-07
T1D	PSC	0.56	0.44	0.68	0.31	6.14E-16
RA	PSC	0.66	0.48	0.85	0.43	2.09E-10
AITD	RA	0.49	0.32	0.68	0.24	9.58E-08
AITD	T1D	0.48	0.36	0.61	0.23	9.35E-14
AITD	PSC	0.53	0.39	0.67	0.28	7.85E-12
CD	PBC	0.67	0.43	0.92	0.45	3.27E-06
CD	SjS	0.71	0.42	1.00	0.50	8.05E-06

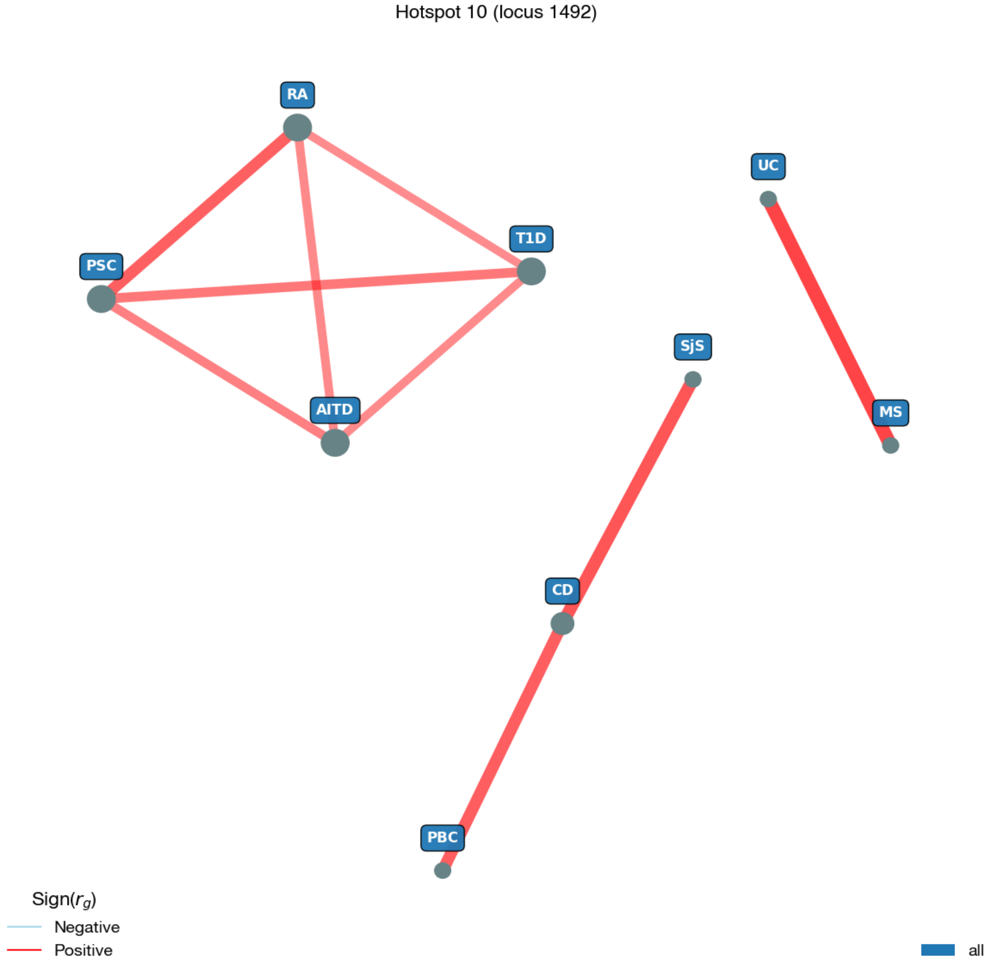

**Top 11. Locus 2203**<br>chr17: 37361179 - 38880481<br>Genes: *STAC2; LOC101929578; FBXL20; MED1; CDK12; NEUROD2; PPP1R1B; STARD3; TCAP; PNMT; PGAP3; ERBB2; MIEN1; GRB7; IKZF3; ZPBP2; GSDMB; ORMDL3; LOC101928947; LRRC3C; GSDMA; PSMD3; CSF3; MED24; THRA; NR1D1; MSL1; CASC3; RAPGEFL1; WIPF2; CDC6; RARA; GJD3; TOP2A; IGFBP4; TNS4; CCR7; SMARCE1; KRT222; KRT24*						
**Phenotype1**	**Phenotype2**	**Rho**	**CI.lower**	**CI.upper**	**R2**	**P**
MS	UC	0.79	0.49	1.00	0.62	1.84E-06
CD	RA	0.61	0.37	0.89	0.37	2.34E-06
T1D	PBC	0.76	0.52	1.00	0.58	6.27E-07
SS	PBC	0.77	0.52	1.00	0.59	1.84E-06
MS	CD	0.68	0.38	1.00	0.46	2.41E-05
UC	CD	0.69	0.53	0.84	0.47	2.55E-10
UC	PBC	0.75	0.57	0.92	0.56	2.20E-09
CD	PBC	0.68	0.50	0.86	0.47	3.32E-08

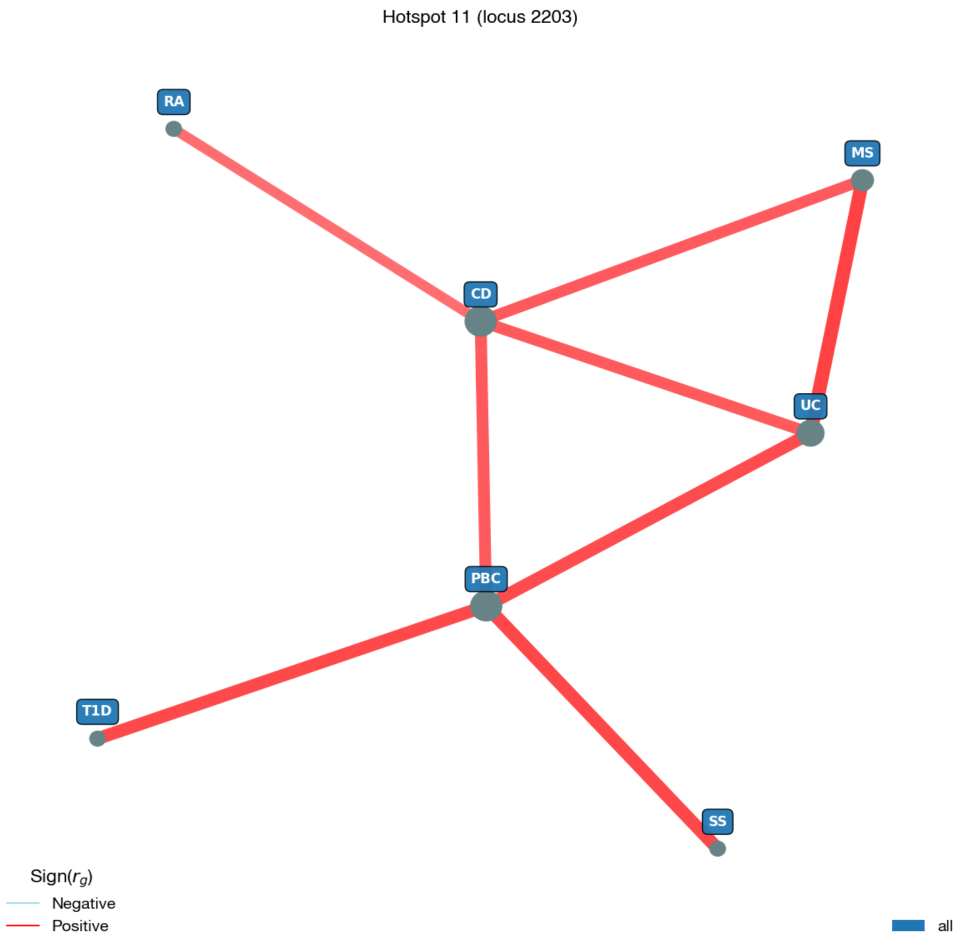

**Top 12. Locus 963**<br>chr6: 32454578 - 32539567<br>Genes: *HLA-DRB5*						
**Phenotype1**	**Phenotype2**	**Rho**	**CI.lower**	**CI.upper**	**R2**	**P**
MS	UC	0.84	0.77	0.91	0.71	8.12E-44
T1D	UC	-0.31	-0.41	-0.20	0.09	7.84E-09
PS	PSC	-0.74	-0.95	-0.51	0.54	2.42E-06
MS	RA	0.42	0.38	0.45	0.17	8.39E-108
T1D	PSC	0.52	0.40	0.64	0.27	5.64E-12
AITD	RA	0.34	0.28	0.40	0.11	1.30E-24
MS	PSC	-0.34	-0.44	-0.24	0.12	2.25E-10
AITD	UC	-0.57	-0.66	-0.47	0.32	4.21E-22

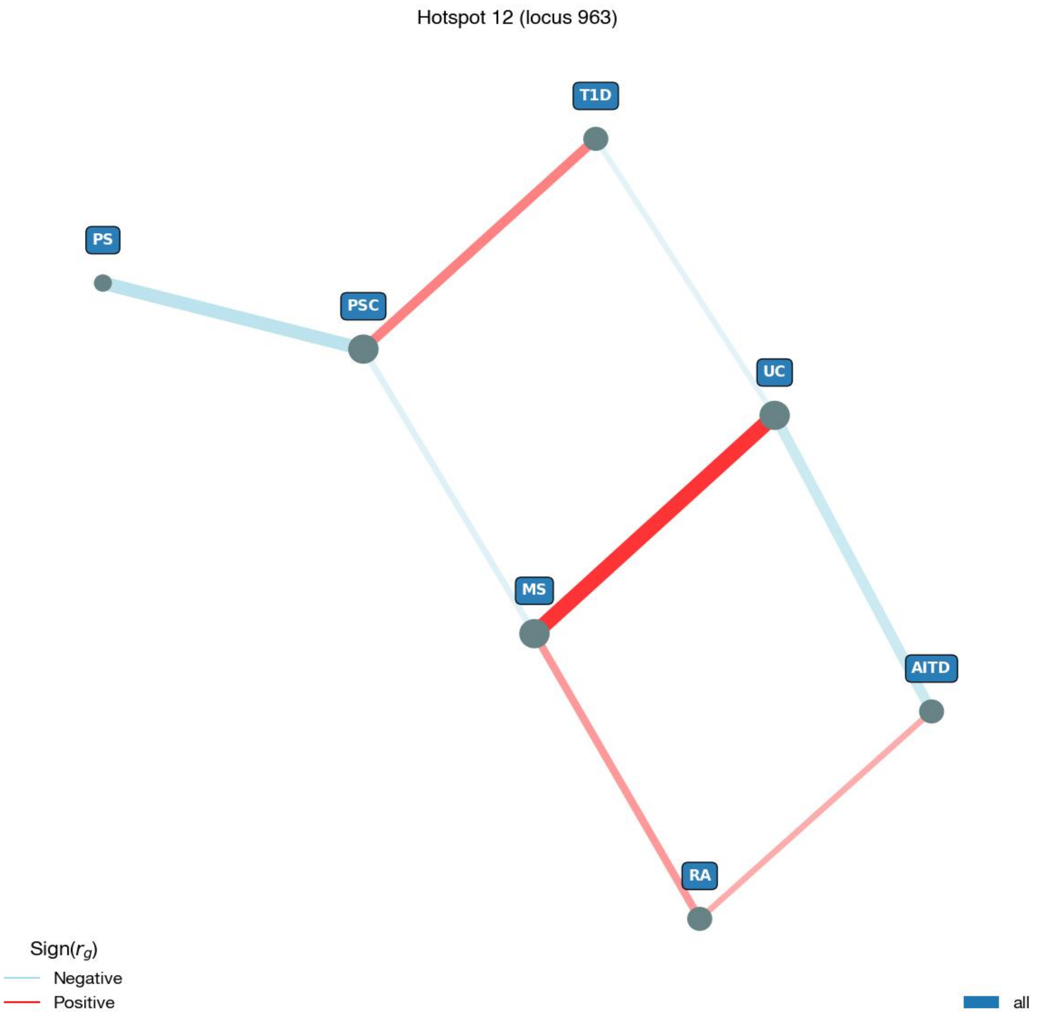

**Top 13. Locus 2251**<br>chr18: 12735559 - 13641479<br>Genes: *PTPN2; SEH1L; CEP192; LDLRAD4*						
**Phenotype1**	**Phenotype2**	**Rho**	**CI.lower**	**CI.upper**	**R2**	**P**
PS	PBC	0.91	0.55	1.00	0.83	5.27E-06
CD	RA	0.72	0.55	0.90	0.51	3.16E-13
UC	RA	0.96	0.72	1.00	0.92	5.79E-12
T1D	RA	0.64	0.49	0.82	0.41	5.87E-15
RA	PS	0.41	0.26	0.58	0.17	3.25E-07
CD	PS	0.44	0.33	0.55	0.19	1.12E-13
T1D	CD	0.45	0.33	0.58	0.21	1.79E-11
UC	CD	0.90	0.74	1.00	0.82	3.45E-12

Hotspot 13 (locus 2251)

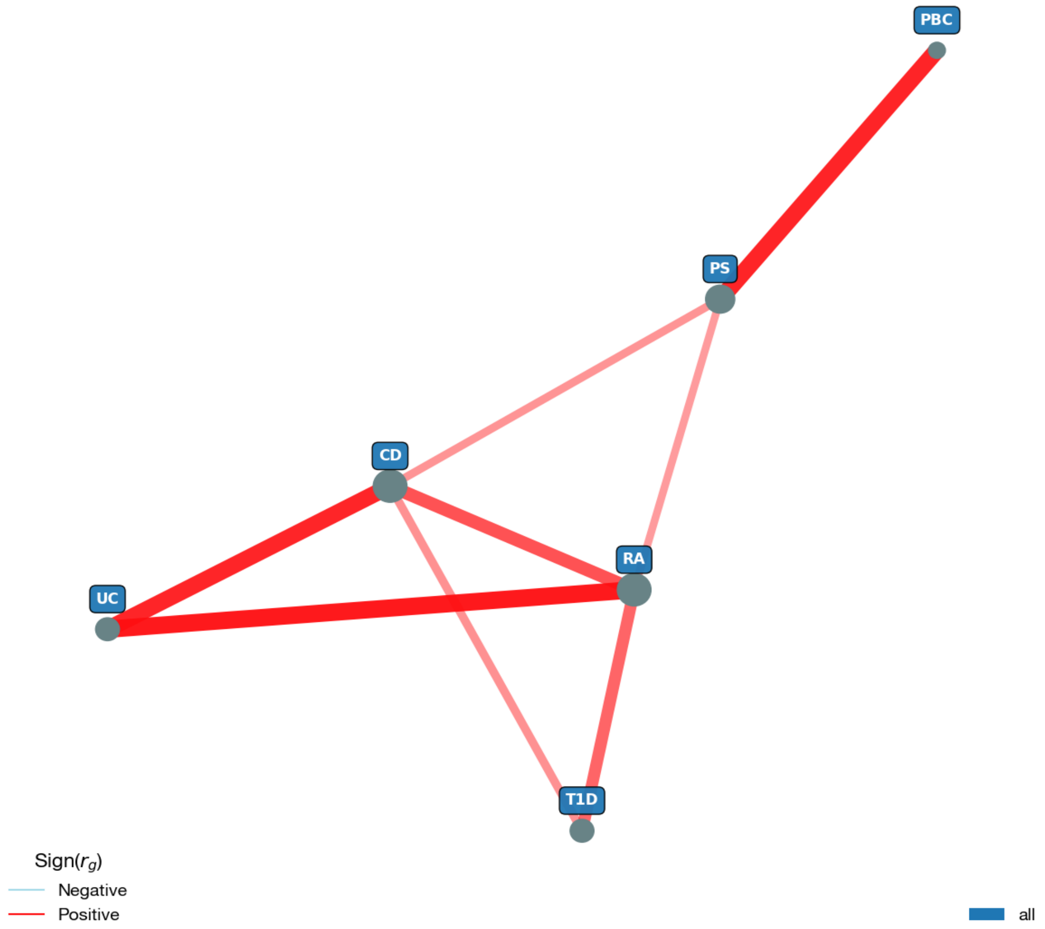

**Top 14. Locus 1063**<br>chr6: 137553213 - 138825721<br>Genes: *OLIG3; TNFAIP3; PERP; ARFGEF3; PBOV1; HEBP2; NHSL1*						
**Phenotype1**	**Phenotype2**	**Rho**	**CI.lower**	**CI.upper**	**R2**	**P**
SjS	PBC	0.98	0.67	1.00	0.96	4.80E-07
UC	RA	0.59	0.41	0.79	0.35	2.68E-09
AITD	RA	0.68	0.47	0.95	0.47	4.12E-09
AITD	PBC	1.00	0.66	1.00	1.00	3.27E-07
AITD	UC	0.75	0.45	1.00	0.56	3.60E-06
RA	PBC	0.91	0.69	1.00	0.84	7.94E-12
RA	SjS	0.78	0.62	0.96	0.61	2.29E-15
UC	PBC	0.81	0.58	1.00	0.66	2.01E-07

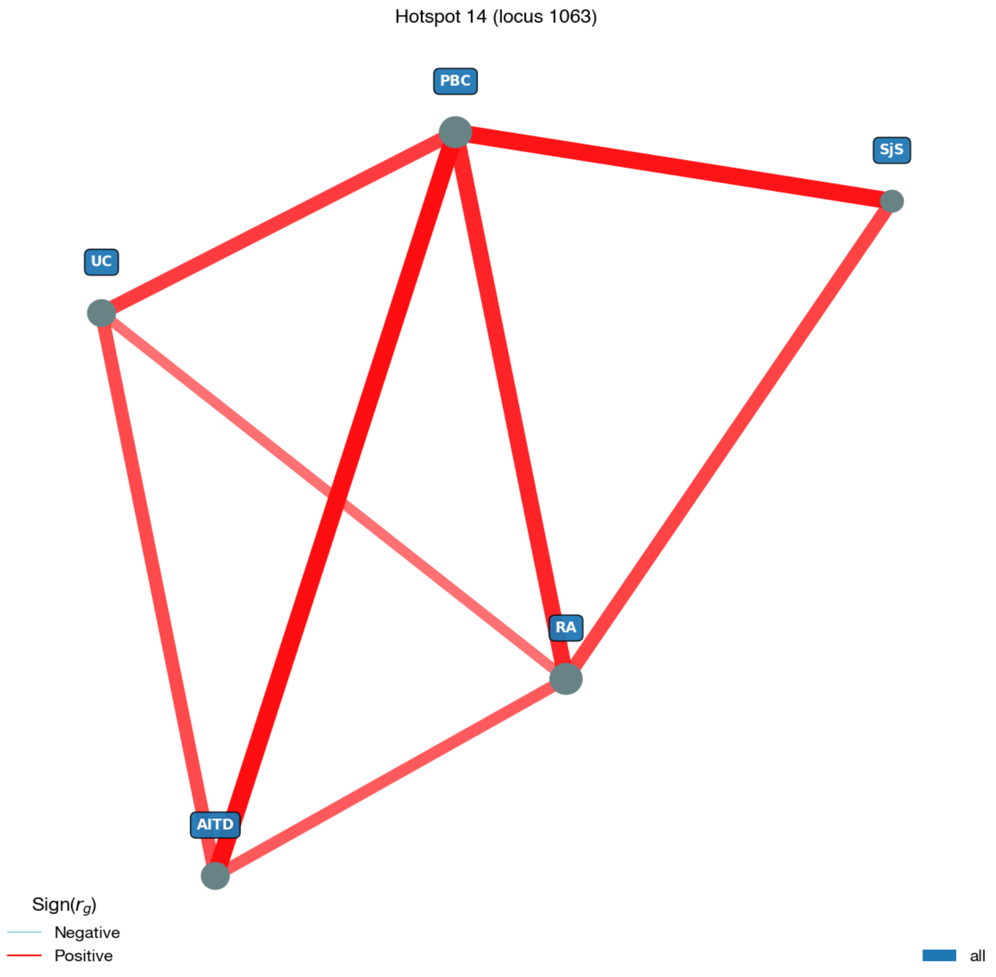

**Top 15. Locus 967**<br>chr6: 32682214 - 32897998<br>Genes: *HLA-DQA2; HLA-DQB2; HLA-DOB; TAP2; PSMB8; TAP1; PSMB9*						
**Phenotype1**	**Phenotype2**	**Rho**	**CI.lower**	**CI.upper**	**R2**	**P**
MS	UC	0.35	0.28	0.41	0.12	7.06E-21
UC	RA	-0.29	-0.36	-0.22	0.08	6.01E-15
MS	RA	-0.20	-0.23	-0.17	0.04	1.95E-44
MS	AITD	-0.31	-0.36	-0.26	0.10	1.16E-26
AITD	RA	0.23	0.18	0.28	0.05	3.16E-21
RA	PBC	0.20	0.12	0.27	0.04	1.06E-06
MS	PBC	-0.28	-0.36	-0.19	0.08	3.33E-11

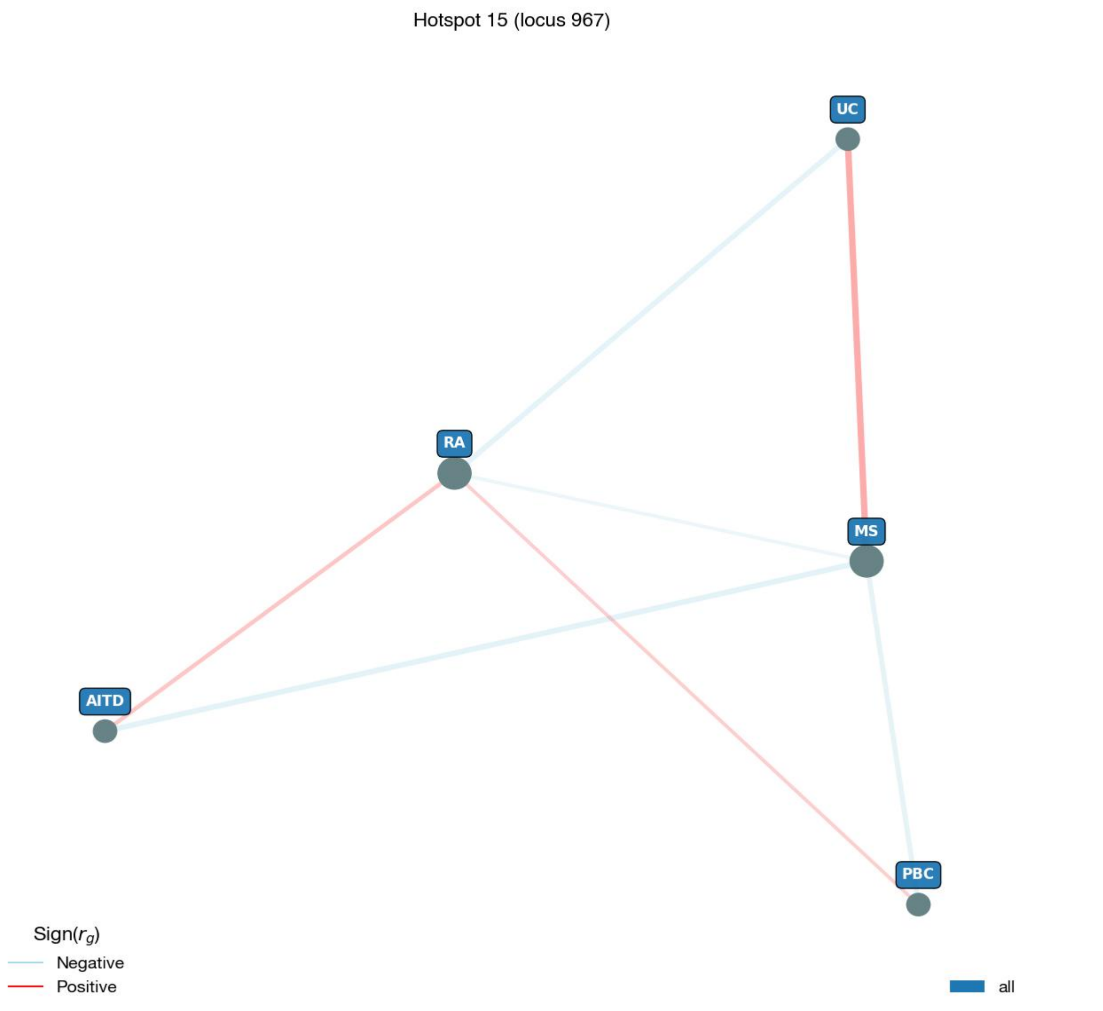

**Top 16. Locus 961**<br>chr6: 31427210 - 32208901<br>Genes: *MICB; MCCD1; DDX39B; ATP6V1G2; NFKBIL1; LTA; TNF; LTB; LST1; NCR3; AIF1; PRRC2A; BAG6; APOM; C6orf47; GPANK1; CSNK2B; LY6G5B; LY6G5C; ABHD16A; LY6G6F; LY6G6D; LY6G6C; C6orf25; DDAH2; CLIC1; MSH5; SAPCD1; VWA7; VARS; LSM2; HSPA1L; HSPA1A; HSPA1B; C6orf48; NEU1; SLC44A4; EHMT2; C2; ZBTB12; CFB; NELFE; SKIV2L; DXO; STK19; C4A; C4B; CYP21A2; TNXB; ATF6B; FKBPL; PRRT1; PPT2; EGFL8; AGPAT1; RNF5;AGER; PBX2; GPSM3; NOTCH4*						
**Phenotype1**	**Phenotype2**	**Rho**	**CI.lower**	**CI.upper**	**R2**	**P**
UC	RA	-0.19	-0.23	-0.15	0.04	2.88E-21
MS	RA	-0.18	-0.21	-0.16	0.03	2.44E-50
MS	AITD	0.08	0.05	0.11	0.01	4.29E-06
AITD	RA	0.11	0.08	0.14	0.01	6.11E-12
AITD	PBC	0.25	0.19	0.32	0.06	1.22E-14
RA	PBC	0.16	0.10	0.22	0.03	1.21E-07
MS	PBC	-0.14	-0.21	-0.08	0.02	7.04E-06

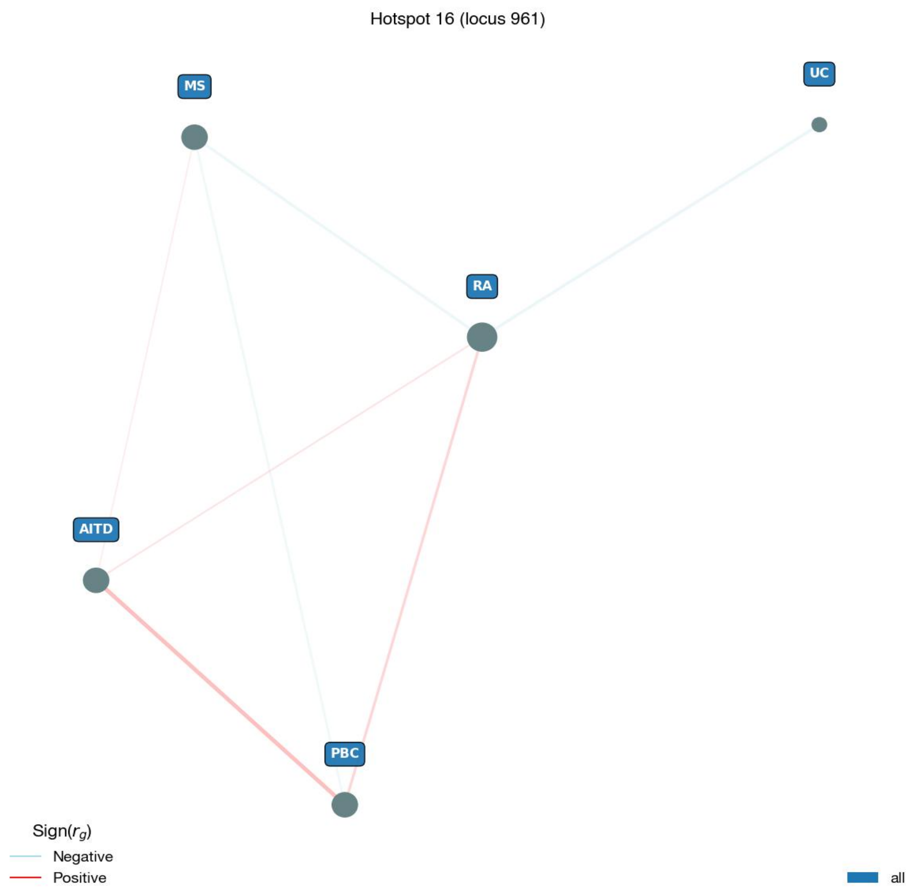

**Top 17. Locus 965**<br>chr6: 32586785 - 32629239<br>Genes: *HLA-DQA1; HLA-DQB1*						
**Phenotype1**	**Phenotype2**	**Rho**	**CI.lower**	**CI.upper**	**R2**	**P**
MS	UC	-0.21	-0.28	-0.15	0.05	5.52E-10
CeD	PBC	-1.00	-1.00	-0.97	1.00	5.82E-11
T1D	PBC	-0.93	-1.00	-0.82	0.87	3.21E-11
UC	RA	-0.23	-0.29	-0.17	0.05	2.16E-14
MS	PBC	-0.97	-1.00	-0.87	0.94	4.79E-09
UC	PBC	0.97	0.88	1.00	0.94	2.86E-08

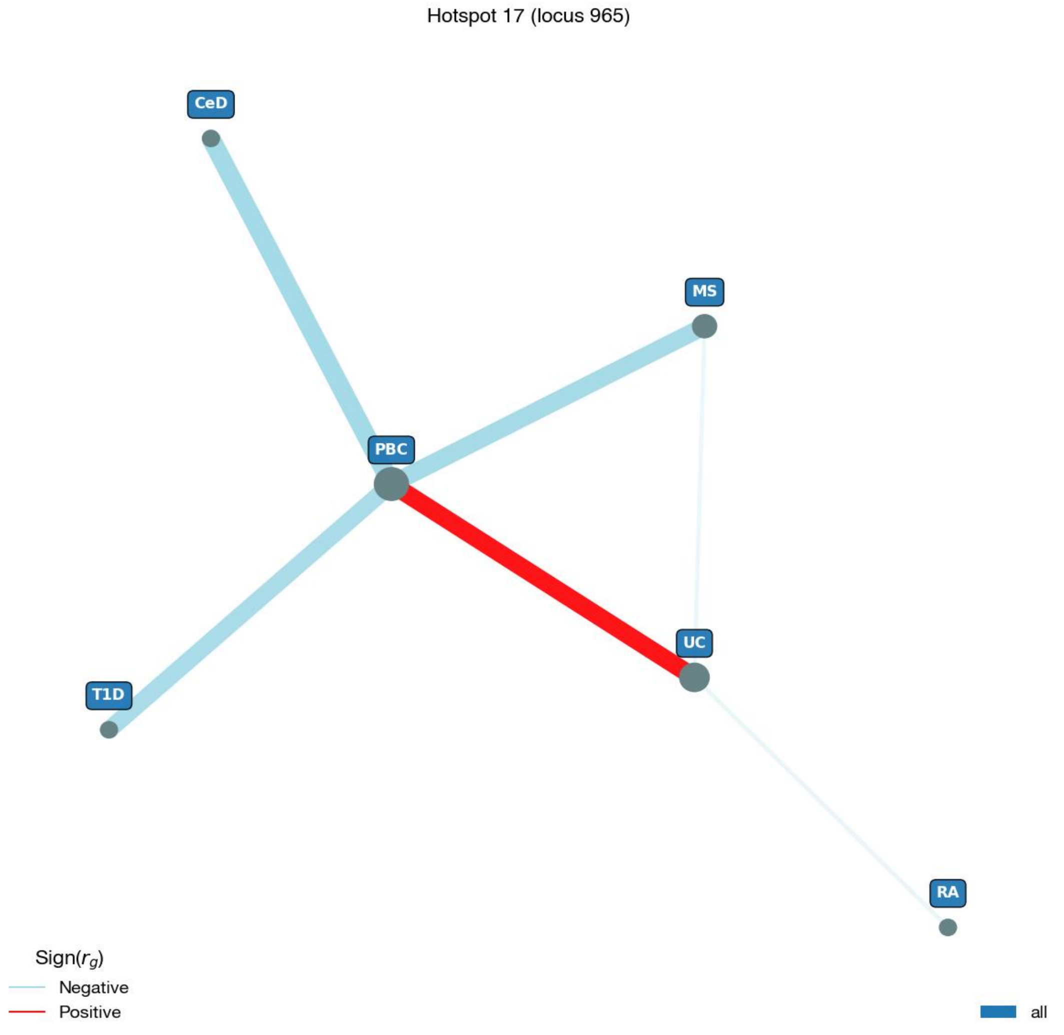

**Top 18. Locus 957**<br>chr6: 30715007 - 31106493<br>Genes: *DDR1; GTF2H4; VARS2; SFTA2; DPCR1; MUC21; MUC22; C6orf15; PSORS1C1; CDSN; PSORS1C2*						
**Phenotype1**	**Phenotype2**	**Rho**	**CI.lower**	**CI.upper**	**R2**	**P**
MS	UC	0.24	0.16	0.33	0.06	2.09E-07
SS	AITD	0.28	0.17	0.40	0.08	7.72E-06
UC	RA	-0.20	-0.29	-0.12	0.04	7.45E-06
MS	RA	-0.20	-0.26	-0.14	0.04	1.18E-10
AITD	PBC	0.33	0.24	0.41	0.11	1.79E-12
RA	PBC	0.24	0.16	0.32	0.06	3.66E-08

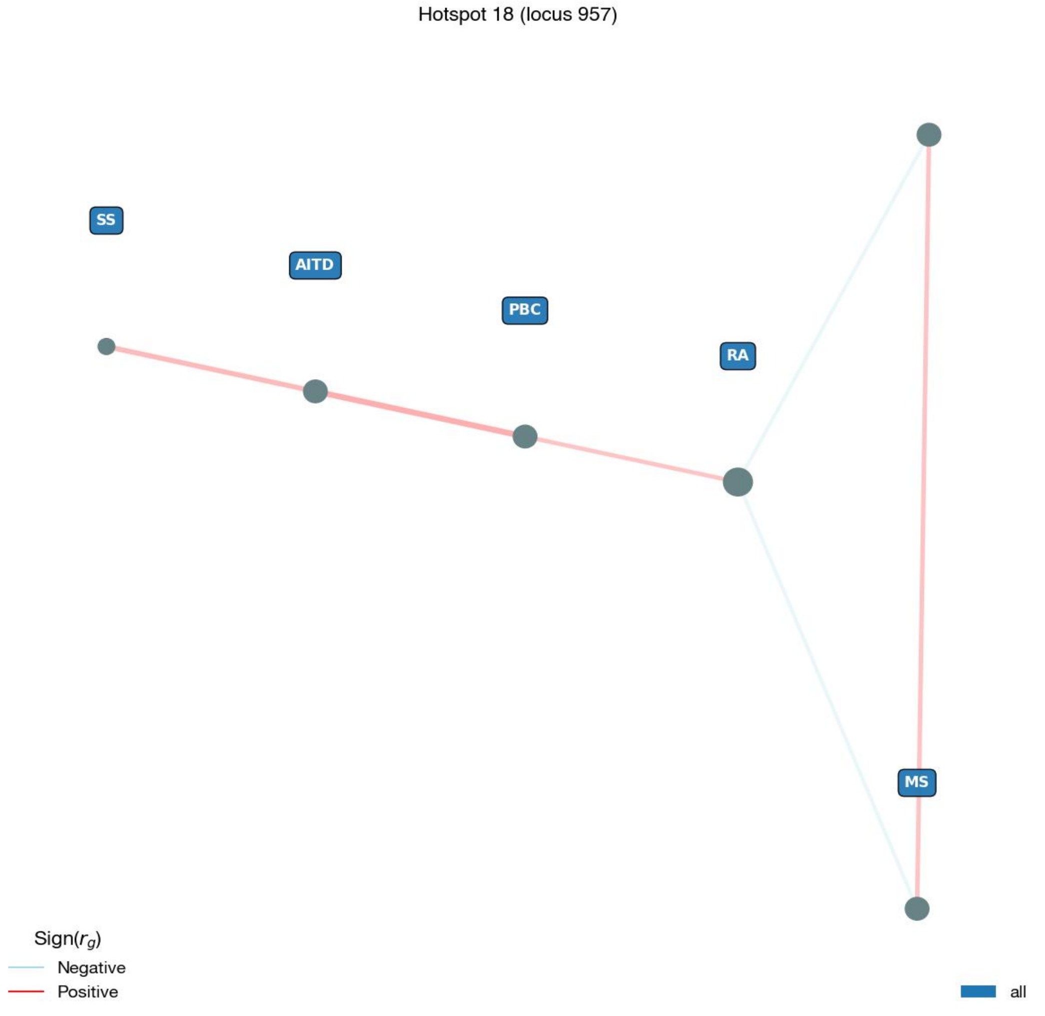

**Top 19. Locus 58**<br>chr1: 67761891 - 68633860<br>Genes: *IL12RB2; SERBP1; GADD45A; GNG12; DIRAS3; WLS*						
**Phenotype1**	**Phenotype2**	**Rho**	**CI.lower**	**CI.upper**	**R2**	**P**
SS	PBC	0.68	0.46	0.95	0.46	6.26E-07
AITD	RA	0.74	0.41	1.00	0.55	2.44E-05
AITD	PBC	0.79	0.54	1.00	0.63	8.78E-08
CD	PS	0.27	0.19	0.35	0.07	2.46E-10
UC	CD	0.74	0.65	0.83	0.55	7.59E-25
RA	PBC	0.90	0.67	1.00	0.80	2.09E-11

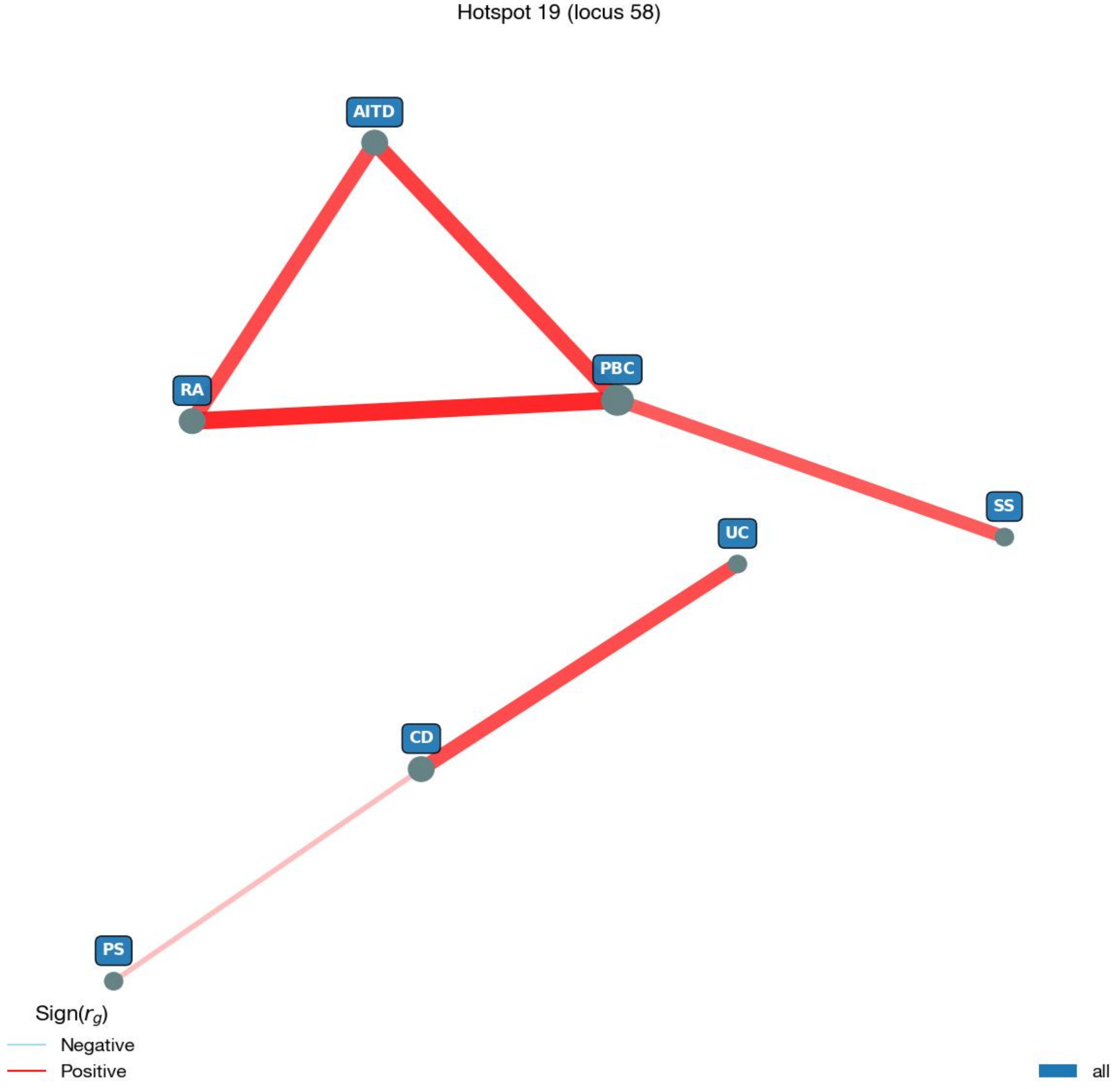

**Top 20. Locus 2483**<br>chr22: 38718590 - 40378783<br>Genes: *KCNJ4; KDELR3; DDX17; DMC1; FAM227A; CBY1; TOMM22; JOSD1; GTPBP1; SUN2; DNAL4; NPTXR; CBX6; APOBEC3A; APOBEC3B; APOBEC3C; APOBEC3D; APOBEC3F; APOBEC3G; APOBEC3H; CBX7; PDGFB; RPL3; SYNGR1; TAB1; MGAT3; MIEF1; ATF4; RPS19BP1; CACNA1I; ENTHD1; GRAP2*						
**Phenotype1**	**Phenotype2**	**Rho**	**CI.lower**	**CI.upper**	**R2**	**P**
SS	PBC	0.79	0.50	1.00	0.62	3.25E-05
SjS	PBC	1.00	0.65	1.00	1.00	2.51E-06
AITD	PBC	0.91	0.59	1.00	0.83	1.57E-06
UC	CD	0.88	0.60	1.00	0.78	4.07E-06
RA	PBC	0.74	0.49	1.00	0.55	6.20E-07

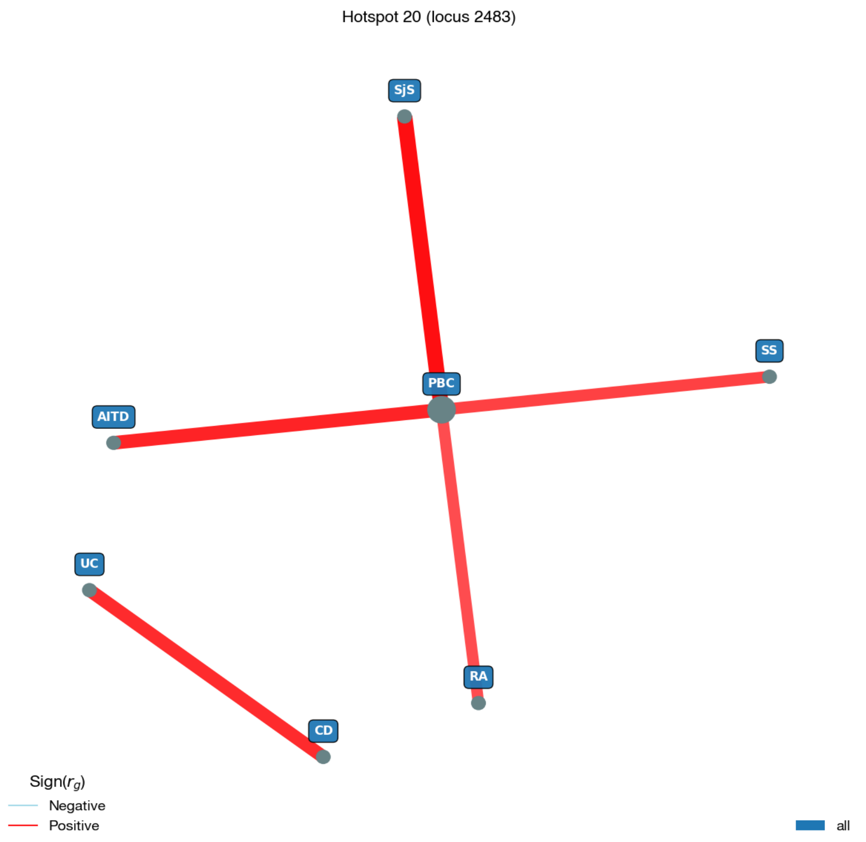

Supplement: Supplementary file 1 [file SupplementaryFile1.pdf]
